# Supplementary material for: Three-Dimensional Human Alveolar Stem Cell Culture Models Reveal Infection Response to SARS-CoV-2
Source: Cell Stem Cell. 2020 Dec 3;27(6):905–919.e10. doi: 10.1016/j.stem.2020.10.004 (PMC7577700; doi:10.1016/j.stem.2020.10.004)
Supplement: Document S1. Figures S1–S6 and Tables S6 and S7 [file mmc1.pdf]

Cell Stem Cell, Volume *27*

Supplemental Information

Three-Dimensional Human Alveolar Stem Cell Culture Models Reveal Infection Response to SARS-CoV-2

Jeonghwan Youk, Taewoo Kim, Kelly V. Evans, Young-Il Jeong, Yongsuk Hur, Seon Pyo Hong, Je Hyoung Kim, Kijong Yi, Su Yeon Kim, Kwon Joong Na, Thomas Bleazard, Ho Min Kim, Mick Fellows, Krishnaa T. Mahbubani, Kourosh Saeb-Parsy, Seon Young Kim, Young Tae Kim, Gou Young Koh, Byeong-Sun Choi, Young Seok Ju, and Joo-Hyeon Lee

Figure S1, related to Figure 1.

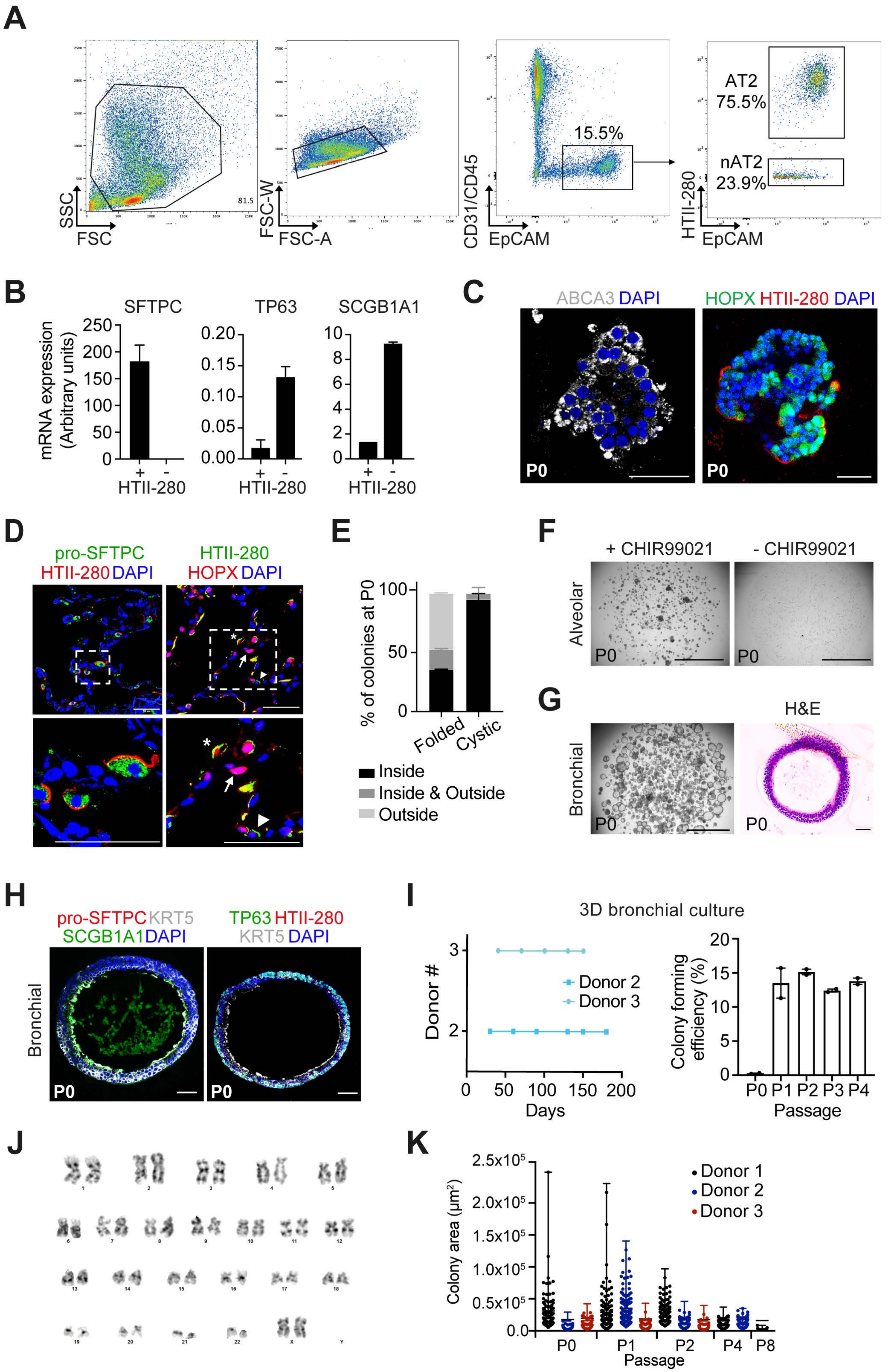

Figure S1. Establishment and characterization of three-dimensional culture of human airway and alveolar type 2 cells. Related to Figure 1.

(A) Representative flow cytometry analysis plot for isolation of hAT2 cells (CD31-CD45-EPCAM+HTII-280+) and non-hAT2 cells (nAT2, CD31-CD45-EPCAM+HTII-280-) from human distal lung parenchymal tissues.

(B) Quantitative PCR (qPCR) analysis of isolated primary human lung epithelial cells. HTII-280+ cells express much higher levels of the AT2 cell marker SFTPC, while HTII-280- cells express virtually no SFTPC and instead express higher levels of the airway markers TP63 (basal) and SCGB1A1 (secretory). Data is the mean ± SEM of arbitrary mRNA expression (n=2, technical replicates).

(C) Representative immunofluorescence (IF) images of primary hAT2 cultures expressing alveolar lineage markers. Left; ABCA3 (for hAT2, white). Right; HOPX (for hAT1-like, green), HTII-280 (for hAT2, red), and DAPI (blue). Scale bar, 50 μm.

(D) IF staining of human distal parenchymal regions of healthy human donor lungs for pro-SFTPC (green, left) and HTII-280 (red, left; green, right), and HOPX (red, right). DAPI (blue). Images are representative of individual donors (n = 3). Scale bar, 50 μm.

(E) Quantification of the folded and cystic 3D structures expressing HTII-280 in inside or/and outside of hAT2 cells in h3ACs (P0). Data is presented as mean ± SEM for 2 individual donor samples (n = 67 for donor 1, n = 50 for donor 2). n=total number of colonies scored.

(F) Representative brightfield images of primary hAT2-derived 3D alveolar cultures (h3ACs) at day 14 established in complete medium (left) or complete medium without CHIR99021 (right). Scale bar, 2000 μm. Images are representative of individual donors (n = 3). P0; passage 0.

(G) Representative brightfield and hematoxylin and eosin (H&E) images of primary HTII-280- derived 3D bronchial (airway) cultures (h3BCs) following 14 days of culture in human airway medium (Sachs et al., 2019). Scale bar, 2000 μm. P0; passage 0.

(H) IF images of h3BCs following 21 days of primary culture in (F). Cultures express airway lineage markers SCGB1A1 (for secretory, green; left), KRT5 (basal, white; left and right), and TP63 (basal, green; right), but not alveolar markers; pro-SFTPC (for hAT2, red, left), HTII-280 (for hAT2, red, right), and DAPI (blue). Scale bar, 50 μm. P0; passage 0.

(I) Serial passage of h3BCs via single cell dissociation at various time points depending on growth from 2 different donors (left). Each point represents a single passage for two separate donor samples (n= at least 3 technical replicates per donor). Quantification of colony forming efficiency for h3BCs at day 14 of culture up to 5 total passages (right). Data are presented as mean ± SEM for n = 2 biological samples. Each point represents the average of 3 technical replicates calculated for each biological sample.

(J) A representative karyotype image of h3ACs with 6 months in vitro culture (P5). No chromosomal aberrations were detected (n = 6, the number of examined hAT2 cells which are arrested in G2/M phase).

(K) Quantification of colony surface area during long-term serial culture of h3ACs. Areas are presented as the mean ± SEM of 300 colonies per passage across 3 technical replicates for 3 donor samples, with colonies consisting of a surface area of more than 1600 μm^2 being included in the analysis. Due to the low number of colonies at P8, only 100 colonies from 2 technical replicates were analyzed. (For each passage, n = 300 for donor 1, n = 300 for donor 2, n = 300 for donor 3 [P0-P4], n = 100 for donor 3 [P8]). n = total number of colonies scored.

Figure S2, related to Figure 2.

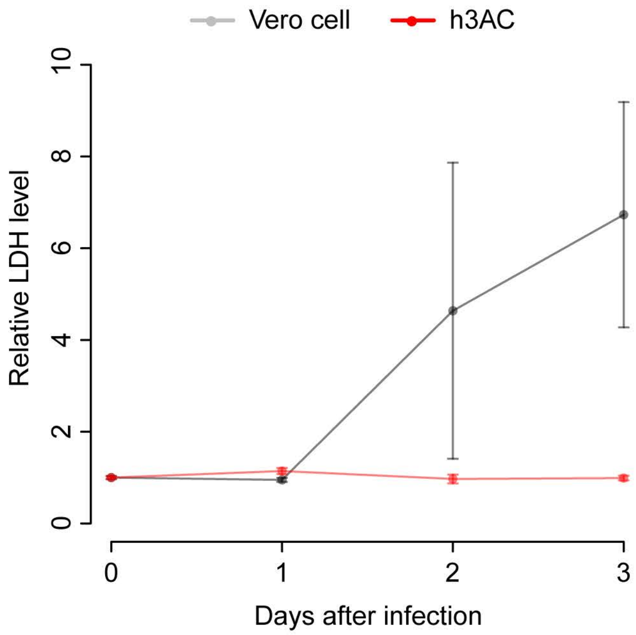

Figure S2. Lactate dehydrogenase cytotoxicity assay of human 3D alveolar cultures and Vero cells. Related to Figure 2.
Lactate dehydrogenase (LDH) is released into culture media when plasma membrane of cells is damaged. The cytotoxicity of SARS-CoV-2 infected Vero cells remarkably increases after 2 dpi, whereas that of SARS-CoV-2 infected h3ACs does not change significantly. Data is presented as mean ± SEM. n = 3 for biological replicates at each time point. h3ACs at passage 0 from 1 donor are used in the experiment.

Figure S3, related to Figure 3.

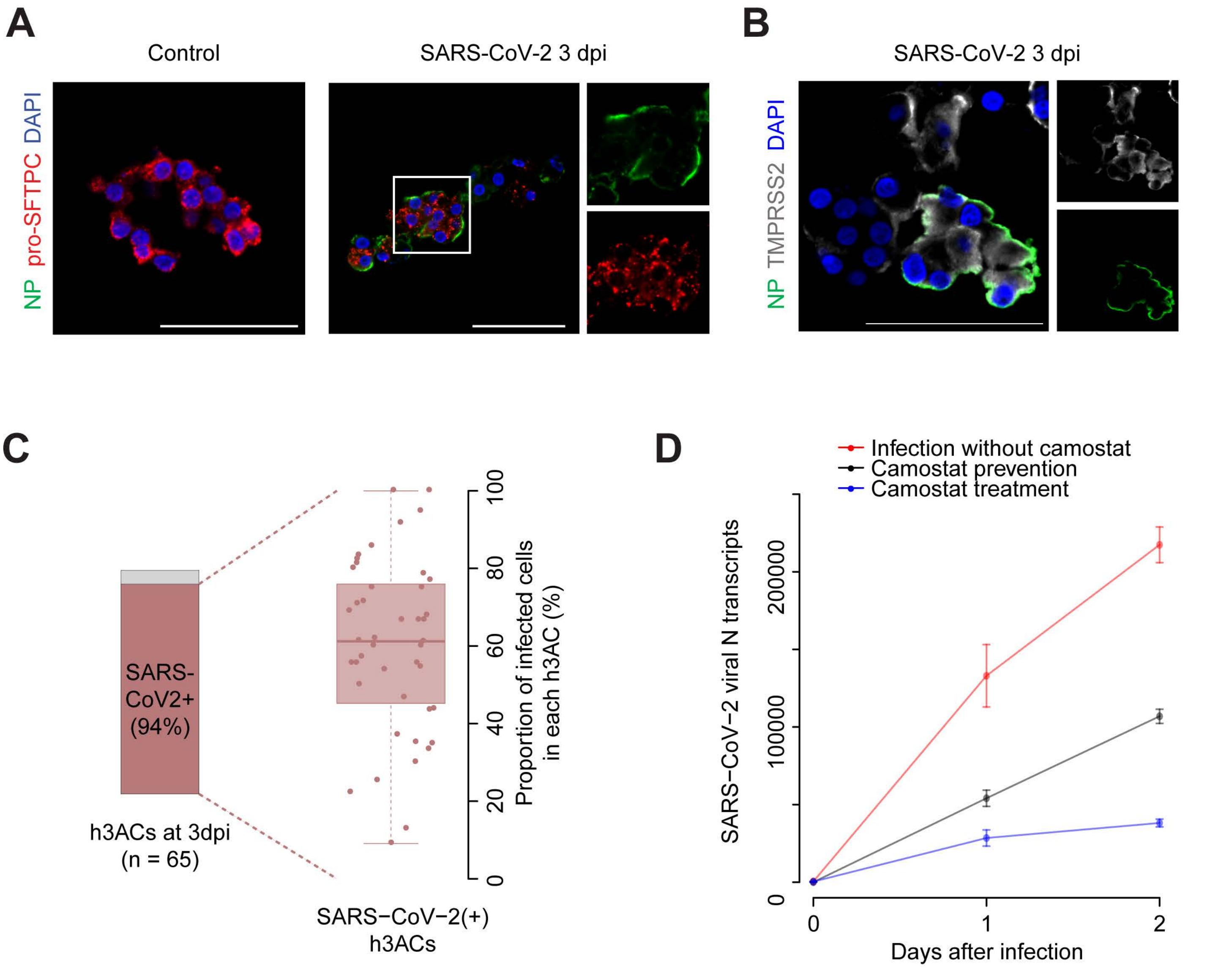

Figure S3. Confocal imaging analysis of infected h3ACs. Related to Figure 3.
(A) Representative IF images of infected h3ACs co-stained with SARS-CoV-2 nucleoprotein (NP, green) and pro-SFTPC (red) at 3 dpi (right). A representative IF image of uninfected control h3ACs is shown (left). Punctated patterns of pro-SFTPC are shown in magnified images (bottom right). Images are representatives of n ≥ 5 replicates of infected h3ACs (P2-3 from 3 donors). Scale bar, 50 μm.
(B) A representative IF image of infected h3ACs co-stained with SARS-CoV-2 nucleoprotein (NP, green) and TMPRSS2 (white) at 3 dpi. Images are representatives of 3 replicates of infected h3ACs (P2 from 2 donors). Scale bar, 50 μm.
(C) Proportion of h3ACs harboring hAT2 cells with viral components at 3 dpi (n = 65 h3ACs from P2-3 of 3 donors, left). The proportion of infected cells in the individual h3AC is depicted in the box plots (right).
(D) Camostat mesylate, a TMPRSS2 inhibitor, partially decreases the infectivity of SARS-CoV-2 in h3ACs. Both prevention (2 hours incubation with camostat mesylate before SARS-CoV-2 infection) and treatment (camostat mesylate treatment 2 hours after SARS-CoV-2 infection) are effective to reduce the burden of SARS-CoV-2 N gene transcripts. n = 3 for technical replicates at each time point. h3ACs with passage 2 from 1 donor are used.

Figure S4, related to Figure 4.

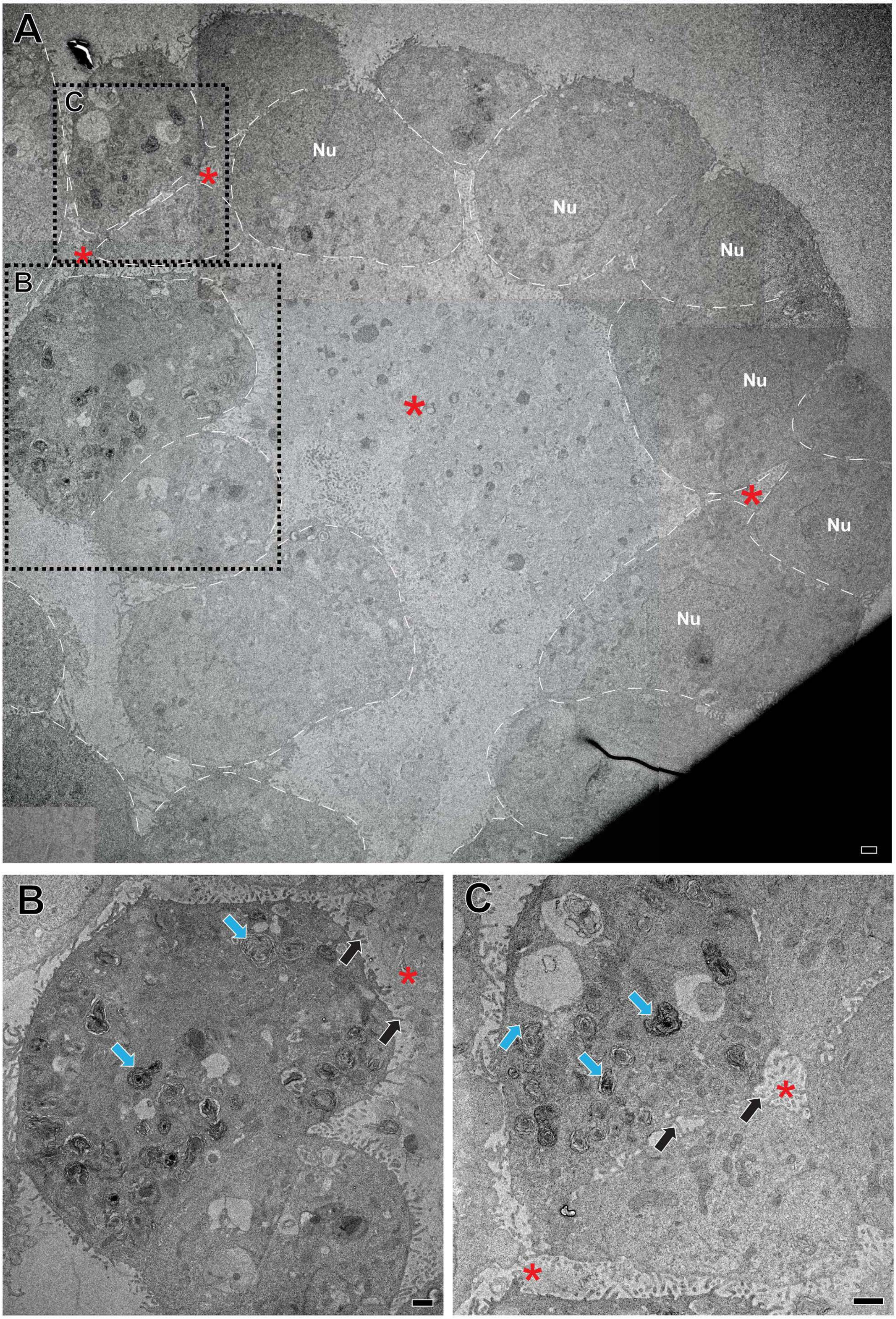

Figure S4. Transmission electron microscopy imaging of uninfected h3ACs. Related to Figure 4.
(A) A representative image of an uninfected h3AC (n = 10 h3ACs from 1 donor; P2). hAT2 cell membrane is delineated with white dashed line. Lumen is shown with a red asterisk. Nu: Nucleus. Data were manually stitched.
(B) A normal hAT2 cell showing multiple lamellar bodies (skyblue arrows). Microvilli in the luminal space of the h3AC are indicated by black arrow. No viral particle or pathologic large vacuoles are observed in the normal hAT2 cell.
(C) Another normal hAT2 cell. Multiple lamellar bodies are also shown in the hAT2 cell (skyblue arrow). Black arrow, microvilli in the lumen; red asterisk, lumen of the h3AC. All scale bars, 1 μm.

Figure S5, related to Figure 5.

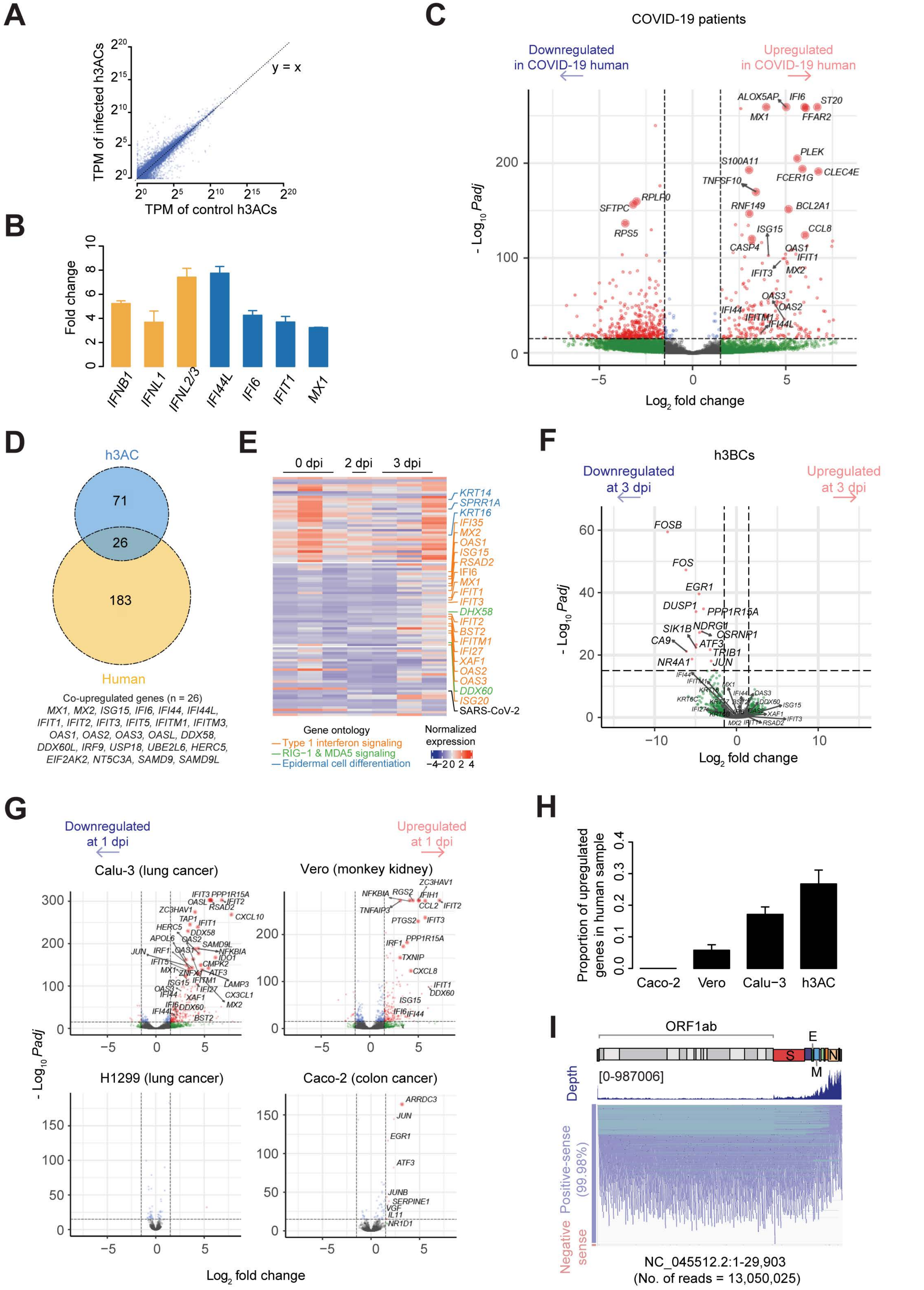

Figure S5. Transcriptome changes of the infected h3ACs. Related to Figure 5.
(A) Correlation of TPM level of each gene in h3ACs at 0 and 3 dpi. Each gene is depicted as a blue dot.
(B) qPCR validation for seven upregulated genes at 3 dpi identified by RNA sequencing. The seven genes include interferon genes (*IFNB1, IFNL1, and INFL2/3*) and ISGs (*IFI44L, IFI6, IFIT1, and MX1*). n = 3 technical replicates for each gene.
(C) Gene expression difference between healthy and SARS-CoV-2 infected human lung tissues (n = 8 from two donors for each group).
(D) A Venn diagram of upregulated DEGs in COVID-19 patients and SARS-CoV-2 infected h3ACs. Among the 97 DEGs, 26.7% of the DEGs, including 15 ISGs, are also increased in human COVID-19 patients.
(E) Heatmap of the most variable 100 genes derived from h3ACs (**Figure 5A, Table S1**) in SARS-CoV-2 infected h3BCs. Normalization of gene expression was calculated using all transcriptome data from 14 samples of h3ACs and h3BCs.
(F) Volcano plot for showing DEGs in h3BCs at 0 and 3 dpi. Expression levels of ISGs were not changed in h3BCs.
(G) Volcano plots for showing DEGs in 2D cell lines such as Calu-3, Vero, NCI-H1299, and Caco-2 cell lines. All DEGs are calculated between 0 and 1 dpi. Expression changes of ISGs are observed only in Calu-3.
(H) The proportion of upregulated DEGs found in COVID-19 patients in the upregulated DEGs of each model.
(I) Positive-sense viral RNAs are dominant (99.98%) over negative-sense viral RNAs in infected h3ACs at 1 dpi.

Figure S6, related to Figures 6 and 7.

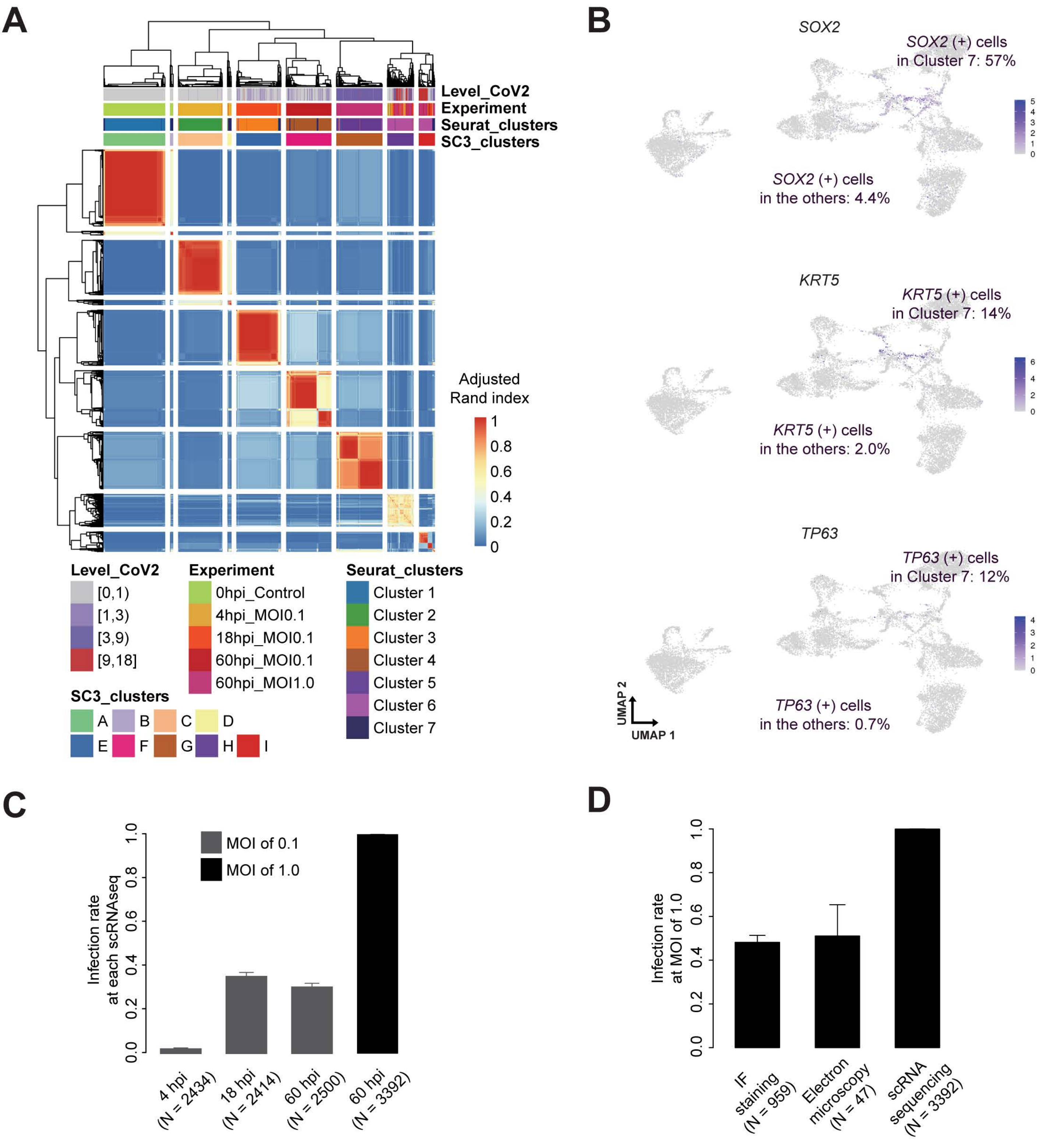

E

Significantly enriched GO biological process

	Gene Ontology Biological Process	Adj. *p-value*
1	Type I interferon signaling pathway (GO:0060337)	1.1×10^{-31}
2	Cellular response to type I interferon (GO:0071357)	2.3×10^{-31}
3	Cytokine-mediated signaling pathway (GO:0019221)	1.4×10^{-17}
4	Negative regulation of viral genome replication (GO:0045071)	5.0×10^{-14}
5	Negative regulation of viral life cycle (GO:1903901)	6.9×10^{-13}

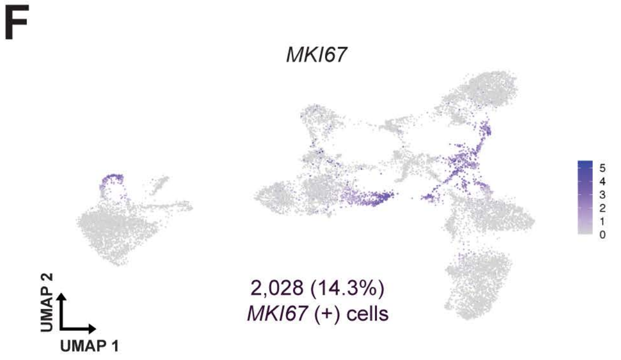

Figure S6. Single-cell transcriptome profiling of infected h3ACs. Related to Figures 6 and 7.
(A) Heatmap of unsupervised clustering of the single-cell RNA sequencing datasets using SC3 packages. The resulting SC3 clusters are in concordance with the Seurat clusters. Level_CoV2: log_2 values of SARS-CoV-2 UMI counts per 10k human UMI counts.
(B) In Cluster 7, the expression level of some airway markers, such as *SOX2, KRT5, TP63,* and *KRT5* are more expressed than other clusters, whereas the expression level of *SFTPB* and *NKX2-1*, hAT2 markers (**Figure 6E**), are reduced.
(C) The proportion of infected cells (≥1 viral UMI counts per 10k human UMI counts) in each experimental condition of single cell transcriptome. In experimental conditions with MOI of 0.1, more than 30% of cells are infected in 18 hpi and 60 hpi, while only 1.9% of cells are infected in 4 hpi. In contrast to MOI of 0.1, most cells (99.9%) at 60 hpi with MOI of 1.0 are infected. Error bars represent 95% of confidence intervals estimated by binomial distribution.
(D) The proportion of infected cells measured by NP+ cells (48.1%) in IF images at 3 dpi (N = 959, total counted number of cells), cells harboring viral particles (51.1%) in electron microscopic images at 2 dpi (N = 47), and cells with ≥1 viral UMI counts per 10k human UMI counts (99.9%) in infected h3ACS with MOI of 1.0 at 3 dpi (N = 3,392). Given the difference of detection methods (RNA or protein), the sensitivity of SARS-CoV-2 infected cells are different among techniques. Error bars represent 95% of confidence intervals estimated by binomial distribution.
(E) Top five significantly enriched gene ontology (GO) biological processes using top 150 variable genes (**Table S5**) selected from single cell transcriptome data. Pathways related to innate cellular responses to viral infection are significantly enriched.
(F) MKI67, a proliferation marker, is expressed not only in uninfected h3ACs, but also in infected h3ACs, which indicates that infected hAT2 cells still have the ability to proliferate.

Supplementary Tables

Table S6. Clinical characteristics of tissue donors. Related to Figures 1 and 2.

Patient ID	Hospital	Sex	Age	Diagnosis	Smoking status	Pack-year	Smoking_quit
1	Seoul National University Hospital	F	64	Lung adenocarcinoma	Never smoker	NA	NA
2	Seoul National University Hospital	F	41	Pulmonary sequestration	Never smoker	NA	NA
3	Seoul National University Hospital	M	57	Lung adenocarcinoma	Ex smoker	8	20 years ago
4	Seoul National University Hospital	M	61	Lung adenocarcinoma	Current smoker	25	0
5	Seoul National University Hospital	M	78	Lung adenocarcinoma	Never smoker	NA	NA
6	Seoul National University Hospital	F	54	Lung adenocarcinoma	Never smoker	NA	NA
7	Seoul National University Hospital	F	48	Lung adenocarcinoma	Never smoker	NA	NA
8	Seoul National University Hospital	F	68	Lung adenocarcinoma	Never smoker	NA	NA
9	Seoul National University Hospital	M	53	Lung adenocarcinoma	ex-smoker	10	14 years ago
10	Seoul National University Hospital	M	83	Lung squamous cell carcinoma	ex-smoker	10	45 years ago
11	Seoul National University Hospital	M	60	Lung adenocarcinoma	ex-smoker	10	25 years ago
12	Addenbrooks Hospital	F	52	Donation after brainstem death	Never smoker	NA	NA
13	Papworth Hospital Research Tissue Bank	F	74	Lung adenocarcinoma	Never smoker	NA	NA
14	Addenbrooks Hospital	F	61	Donation after brainstem death	Never smoker	NA	NA

NA: Not Applicable

Table S7. List of quantitative PCR primers. Related to STAR Methods.

REAGENTS	SOURCE	IDENTIFIER
2019-nCoV_N3 Forward: GGGAGCCTTGAATACACCAAAA	CDC	N/A
2019-nCoV_N3 Reverse: TGTAGCACGATTGCAGCATTG	CDC	N/A
IFI6 Forward: TGATGAGCTGGTCTGCGATCCT	This paper	N/A
IFI6 Reverse: GTAGCCCATCAGGGCACCAATA	This paper	N/A
IFIT1 Forward: GCCTTGCTGAAGTGTGGAGGAA	This paper	N/A
IFIT1 Reverse: ATCCAGGCGATAGGCAGAGATC	This paper	N/A
MX1 Forward: GGCTGTTTACCAGACTCCGACA	This paper	N/A
MX1 Reverse: CACAAAGCCTGGCAGCTCTCTA	This paper	N/A
IFI44L Forward: TGCACTGAGGCAGATGCTGCG	This paper	N/A
IFI44L Reverse: TCATTGCGGCACACCAGTACAG	This paper	N/A
IFNB1 Forward: CTTGGATTCCTACAAAGAAGCAGC	This paper	N/A
IFNB1 Reverse: TCCTCCTTCTGGAACTGCTGCA	This paper	N/A
IFNL1 Forward: GTTCAAATCTCTGTCACCAC	This paper	N/A
IFNL1 Reverse: TTCAGCTTGAGTGACTCTTC	This paper	N/A
IFNL2/3 Forward: GCCAAAGATGCCTTAGAAGAG	This paper	N/A
IFNL2/3 Reverse: CAGAACCTTCAGCGTCAGG	This paper	N/A
TaqMan probe : *SFTPC* (Hs00951326_g1)	Thermo Fischer Scientific	Cat#4331182
TaqMan probe : *TP63* (Hs01114115_m1)	Thermo Fischer Scientific	Cat#4331182
TaqMan probe : *SCGB1A1* (Hs00171092_m1)	Thermo Fischer Scientific	Cat#4331182
