# Supplementary material for: Three-Dimensional Human Alveolar Stem Cell Culture Models Reveal Infection Response to SARS-CoV-2
Source: Cell Stem Cell. 2020 Dec 3;27(6):905–919.e10. doi: 10.1016/j.stem.2020.10.004 (PMC7577700; doi:10.1016/j.stem.2020.10.004)
Supplement: Document S2. Article plus Supplemental Information [file mmc7.pdf]

Cell Stem Cell

Article

Three-Dimensional Human Alveolar Stem Cell Culture Models Reveal Infection Response to SARS-CoV-2

Graphical Abstract

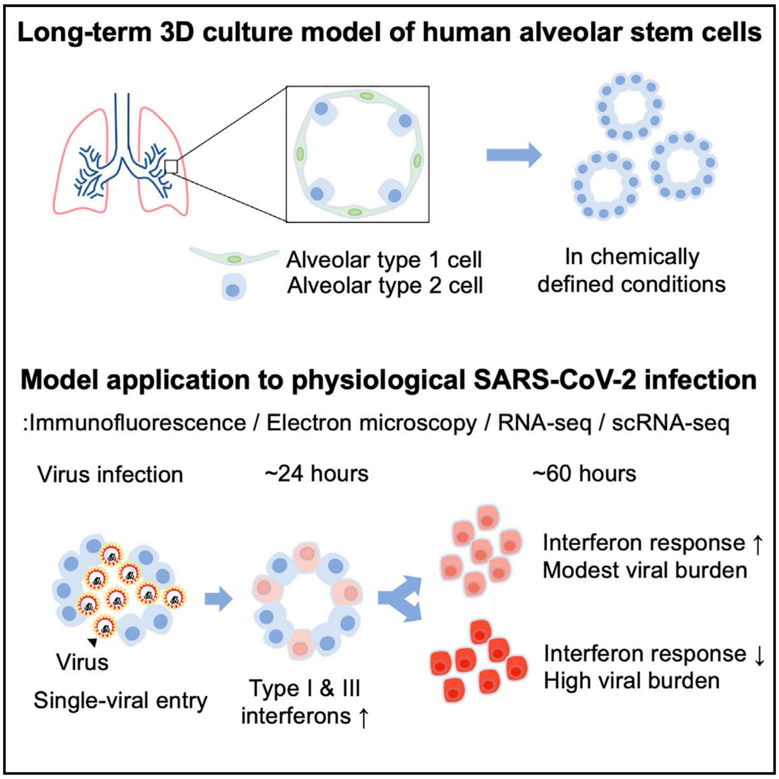

Highlights

- Long-term 3D culture of primary hAT2 cells in chemically defined conditions
- SARS-CoV-2 infects *in vitro* hAT2 models with substantial ultrastructural changes
- Single-cell RNA-seq finds two cellular states of distinct innate immune phenotypes
- Mutation analysis reveals single SARS-CoV-2 entry to a hAT2 cell for full infection

Authors

Jeonghwan Youk, Taewoo Kim, Kelly V. Evans, ..., Byeong-Sun Choi, Young Seok Ju, Joo-Hyeon Lee

Correspondence

ytkim@snu.ac.kr (Y.T.K.),
gykoh@kaist.ac.kr (G.Y.K.),
byeongsun@korea.kr (B.-S.C.),
ysju@kaist.ac.kr (Y.S.J.),
jhl62@cam.ac.uk (J.-H.L.)

In Brief

To study infection response to SARS-CoV-2, Youk et al. developed a 3D culture model of human alveolar stem cells in chemically defined conditions. They reveal that SARS-CoV-2 productively infects alveolar type 2 cells, resulting in an innate immune response. Full infection of individual cells was exhibited from a single viral entry.

Youk et al., 2020, Cell Stem Cell *27*, 905–919
December 3, 2020 © 2020 The Author(s). Published by Elsevier Inc.
https://doi.org/10.1016/j.stem.2020.10.004

Cell Stem Cell

CellPress
OPEN ACCESS

Article

Three-Dimensional Human Alveolar Stem Cell Culture Models Reveal Infection Response to SARS-CoV-2

Jeonghwan Youk,[1,2,14] Taewoo Kim,[1,14] Kelly V. Evans,[3,4,14] Young-Il Jeong,[5,14] Yongsuk Hur,[6,14] Seon Pyo Hong,[7,14] Je Hyoung Kim,[5] Kijong Yi,[1] Su Yeon Kim,[1] Kwon Joong Na,[8] Thomas Bleazard,[9] Ho Min Kim,[1,10] Mick Fellows,[11] Krishnaa T. Mahbubani,[12] Kourosh Saeb-Parsy,[12] Seon Young Kim,[13] Young Tae Kim,[8,*] Gou Young Koh,[7,*] Byeong-Sun Choi,[5,*] Young Seok Ju,[1,2,15,*] and Joo-Hyeon Lee[3,4,*]

[1]Graduate School of Medical Science and Engineering, Korea Advanced Institute of Science and Technology, Daejeon 34141, Republic of Korea

[2]GENOME INSIGHT, Inc., Daejeon 34051, Republic of Korea

[3]Wellcome-MRC Cambridge Stem Cell Institute, Jeffrey Cheah Biomedical Centre, University of Cambridge, Cambridge CB2 A0W, UK

[4]Department of Physiology, Development and Neuroscience, University of Cambridge, Cambridge CB2 3EL, UK

[5]Division of Viral Disease Research, Center for Infectious Diseases Research, Korea National Institute of Health, Korea Centers for Disease Control and Prevention, Cheongju 28159, Republic of Korea

[6]BioMedical Research Center, Korea Advanced institute of Science and Technology, Daejeon 34141, Republic of Korea

[7]Center for Vascular Research, Institute for Basic Science, Daejeon 34126, Republic of Korea

[8]Department of Thoracic and Cardiovascular Surgery, Seoul National University Hospital, Seoul National University Cancer Research Institute, Seoul 03080, Republic of Korea

[9]The National Institute for Biological Standards and Control, Blanche Lane, South Mimms, Potters Bar, Hertfordshire EN6 3QG, UK

[10]Center for Biomolecular and Cellular Structure, Institute for Basic Science, Daejeon 34126, Republic of Korea

[11]Clinical Pharmacology and Safety Sciences, R&D, AstraZeneca, Cambridge, UK

[12]Department of Surgery and Cambridge NIHR Biomedical Research Centre, Biomedical Campus, University of Cambridge, Cambridge CB2 2QQ, UK

[13]Department of Laboratory Medicine, Chungnam National University College of Medicine, Daejeon 35015, Republic of Korea

[14]These authors contributed equally

[15]Lead Contact

*Correspondence: ytkim@snu.ac.kr (Y.T.K.), gykoh@kaist.ac.kr (G.Y.K.), byeongsun@korea.kr (B.-S.C.), ysju@kaist.ac.kr (Y.S.J.), jhl62@cam.ac.uk (J.-H.L.)

https://doi.org/10.1016/j.stem.2020.10.004

SUMMARY

Severe acute respiratory syndrome coronavirus 2 (SARS-CoV-2), which is the cause of a present pandemic, infects human lung alveolar type 2 (hAT2) cells. Characterizing pathogenesis is crucial for developing vaccines and therapeutics. However, the lack of models mirroring the cellular physiology and pathology of hAT2 cells limits the study. Here, we develop a feeder-free, long-term, three-dimensional (3D) culture technique for hAT2 cells derived from primary human lung tissue and investigate infection response to SARS-CoV-2. By imaging-based analysis and single-cell transcriptome profiling, we reveal rapid viral replication and the increased expression of interferon-associated genes and proinflammatory genes in infected hAT2 cells, indicating a robust endogenous innate immune response. Further tracing of viral mutations acquired during transmission identifies full infection of individual cells effectively from a single viral entry. Our study provides deep insights into the pathogenesis of SARS-CoV-2 and the application of defined 3D hAT2 cultures as models for respiratory diseases.

INTRODUCTION

Currently, coronavirus disease 2019 (COVID-19), caused by severe acute respiratory syndrome coronavirus 2 (SARS-CoV-2), is spreading globally (Wu et al., 2020), and more than 35.8 million confirmed cases and 1.05 million deaths have been reported worldwide as of October 7, 2020. The alveoli in lung tissues are the main target tissues for these emerging viruses, especially in patients with SARS-CoV-2-associated pneumonia (Muus et al., 2020).

To develop strategies for efficient prevention, diagnosis, and treatment, the characteristics of new viruses, including mechanisms of cell entry and transmission, kinetics in replication and transcription, host reactions, and genome evolution, should be accurately understood ideally in its target cell types. Essential features of SARS-CoV-2 virus, including ACE2 dependency for cellular entry, patterns of viral transcription, and structure of the spike protein, have been identified (Hoffmann et al., 2020; Kim et al., 2020a; Walls et al., 2020; Wang et al., 2020). However, most findings have been obtained from

This is an open access article under the CC BY license (http://creativecommons.org/licenses/by/4.0/).

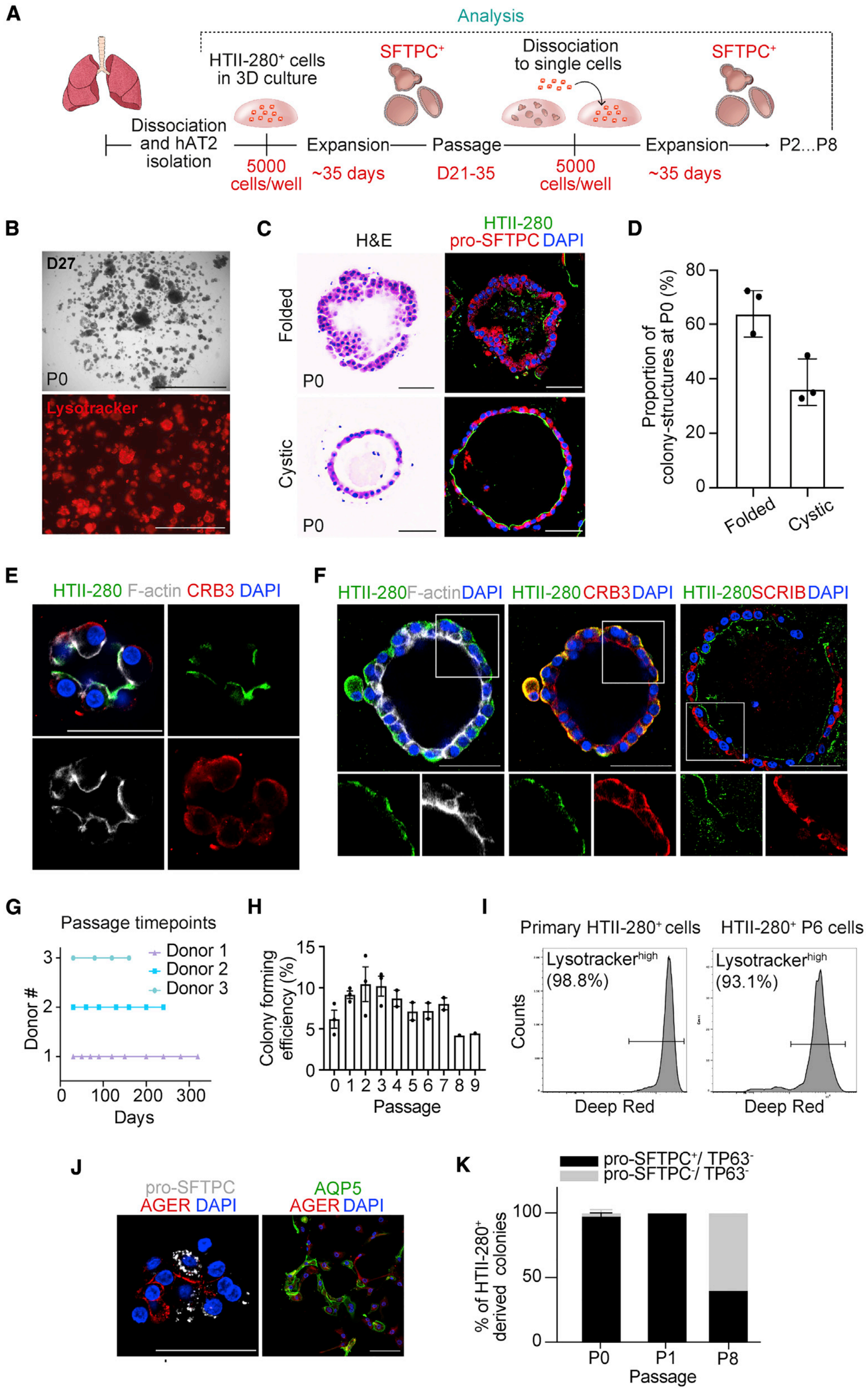

(legend on next page)

CellPress
OPEN ACCESS

experiments using nonphysiological cell lines (Chu et al., 2020); model animals such as transgenic mice expressing human angiotensin-converting enzyme 2 (Bao et al., 2020), ferrets (Kim et al., 2020c), and golden hamsters (Sia et al., 2020); or simple observation in clinical cohorts (Wöllfel et al., 2020) and/or inference from *in silico* computational methods (Andersen et al., 2020; Forster et al., 2020; Shang et al., 2020). A number of studies utilizing stem-cell-based models have been recently established for various tissues (Huang et al., 2020; Jacob et al., 2020; Lamers et al., 2020; Ramani et al., 2020; Yang et al., 2020). However, without competent human alveolar model systems derived from primary tissues, controlled experiments designed to understand virus-host interactions or subsequent immune reactions or detect personal genome variants causing susceptibility to viral infection are challenging. Studies of COVID-19, and respiratory infectious diseases more generally, have been limited by the lack of physiological models that recapitulate normal alveolar physiology and pathology.

Development of organotypic mini-organ models, or organoids, has enabled various physiologic and pathological studies using human-derived tissues *in vitro* (Bartfeld et al., 2015; Fatehullah et al., 2016; Heo et al., 2018). Organoid models established from the human kidney, intestine, and airway have been used to investigate SARS-CoV-2 viral pathogenesis (Elbadawi and Efferth, 2020; Lamers et al., 2020; Monteil et al., 2020). However, the cellular response of human alveolar type 2 (hAT2) cells to SARS-CoV-2 remains elusive due to difficulty in the long-term expansion of pure hAT2 cells. A recent study utilized a model of hAT2 cells derived from human induced pluripotent stem cells to show aspects of SARS-CoV-2 infection (Huang et al., 2020). However, the inability to differentiate into alveolar type 1 (AT1) cells and assess potential age- and/or disease-related viral effects limits the understanding of infection response in primary alveolar lung tissues. In this study, we develop a technique for long-term, feeder-free human three-dimensional (3D) alveolar type 2 cell cultures (hereafter referred to as h3ACs) established from single primary hAT2 cells that serve as stem cells in adult alveolar tissues (Barkauskas et al., 2013). Using our h3AC models, we demonstrate phenotypic changes of hAT2 cells induced by SARS-CoV-2 infection by multi-dimensional methods.

RESULTS

Establishing the 3D Cultures of hAT2 Cells with Chemically Defined Conditions

We developed chemically defined culture conditions for growing hAT2 cells, which were significantly improved from previous feeder-based systems (Barkauskas et al., 2013; Kathiriya et al., 2020). This allowed for the self-organization of single hAT2 cells into alveolar-like 3D structures with defined factors that support the molecular and functional identity of primary-tissue-derived hAT2 cells over multiple passages. Briefly, single-cell dissociated hAT2 cells derived from distal parenchymal regions of healthy donor lungs were isolated by fluorescence-activated cell sorting (FACS) for the hAT2 cell surface marker HTII-280 ($CD31^{-}CD45^{-}EpCAM^{+}HTII\text{-}280^{+}$) (Figures 1A and S1A) (Barkauskas et al., 2013; Gonzalez et al., 2010). Approximately, HTII-280^{+} cells represented 75% of the total EPCAM^{+} cell population (Figure S1A). Quantitative PCR (qPCR) analysis confirmed that the hAT2 cell marker *SFTPC* was highly expressed in isolated HTII-280^{+} cells, while the basal cell marker *TP63* and the secretory cell marker *SCGB1A1* were highly expressed in HTII-280^{-} cells (Figure S1B). Isolated HTII-280^{+} cells were then embedded in Matrigel for 3D culture supplemented with CHIR99021, RSPO1 (R-spondin 1), FGF7, FGF10, epidermal growth factor (EGF), NOG (NOGGIN), and SB431542, factors that have been implicated in lung development and growth of human embryonic lung tip cells (Nikolić et al., 2017). Under this condition, hAT2 single cells created 3D cellular structures with heterogeneous size and morphology, including both folded and cystic-like structures (Figures 1B–1D). They consisted of mature hAT2 cells expressing pro-SFTPC (pro-surfactant protein C), HTII-280, and ABCA3 and exhibiting uptake of Lysotracker, a fluorescent dye that stains acidic

Figure 1. Long-Term, 3D Cultures of hAT2 Cells in Chemically Defined Conditions

(A) Schematic diagram outlining our h3AC method.

(B) A representative image of primary human alveolar type 2 (hAT2)-derived three-dimensional (3D) structures (h3ACs) from freshly isolated HTII-280^{+} cells at day 27 in culture (top) and with Lysotracker (bottom; red). P0, passage 0. Scale bars represent 2,000 μm (top) and 1000 μm (bottom).

(C) Morphological heterotypic colony formation from isolated hAT2 cells in primary h3ACs (P0). Hematoxylin and eosin (H&E; left) and IF staining (right) for HTII-280 (green), pro-SFTPC (red), and DAPI (blue). Scale bar, 50 μm.

(D) Quantification of the folded and cystic 3D structures in primary h3ACs (P0). Data presented are the mean ± SEM for three individual donor samples (n = 67 for donor 1, n = 50 for donor 2, and n = 50 for donor 3; n = total number of colonies scored).

(E) IF images of primary h3ACs expressing HTII-280 (green), F-actin (white), CRB3 (red), and DAPI (blue). Scale bar, 50 μm.

(F) IF images of primary h3ACs expressing HTII-280 (green), F-actin (white), CRB3 (red), SCRIB (red), and DAPI (blue). Scale bar, 50 μm.

(G) Serial passage of h3ACs via single-cell dissociation at various time points depending on growth from three individual donors. Each point represents a single passage for n = 3 individual donor samples with more than three technical replicates.

(H) Quantification of colony-forming efficiency for h3ACs at day 14 of culture up to nine total passages (colony-forming efficiency is defined as the number of colonies formed/number of cells plated per well as a percentage). Data are presented as mean ± SEM for three individual donor samples (n = 3 biological samples). Each point represents the average of three technical replicates calculated for each biological sample (except passage 8 [P8], where n = 2).

(I) Flow cytometry analysis of Lysotracker (deep red) uptake in freshly isolated HTII-280^{+} cells and dissociated h3ACs at passage 6 (P6; 6 months in culture).

(J) IF images of hAT1 cells, induced by 2D-plating of cultured h3ACs at passage 2. Pro-SFTPC (hAT2, white), AGER (hAT1, red), AQP5 (hAT1, green), and DAPI (blue). Scale bar, 50 μm.

(K) Quantification of h3ACs expressing pro-SFTPC and TP63 over multiple passages (P0, passage 1 [P1], and P8). h3ACs were classified based on IF staining for pro-SFTPC (hAT2), TP63 (basal), or lack of both markers (pro-SFTPC^{-} TP63^{-}). The mean percentage of total colonies per well represented by each class of colony is shown. Data are presented as mean ± SEM at each passage (n = 131 for P0, n = 55 for P1, and n = 25 for P8). Three donor samples were used, except for P8 (where one donor sample was used). n = total number of colonies scored across passages.

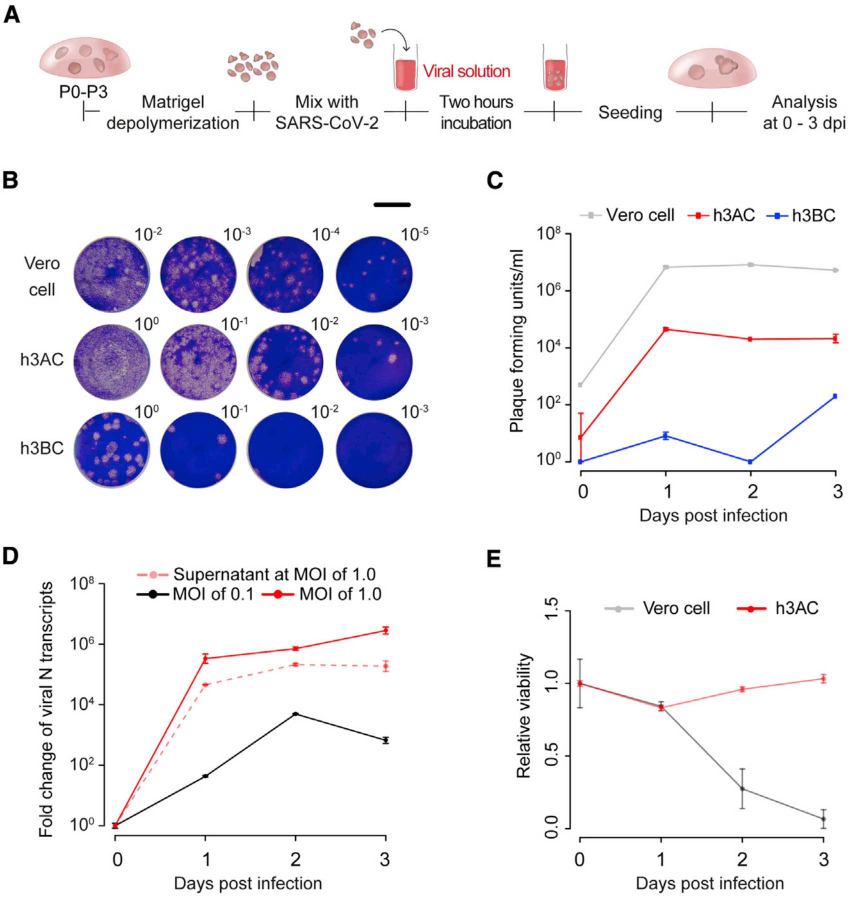

Figure 2. SARS-CoV-2 Infectivity in Human 3D Alveolar and Bronchial Cultures

(A) Schematic diagram outlining the method for SARS-CoV-2 infection in h3ACs and human 3D bronchial cultures (h3BCs). To infect these models, h3ACs and h3BCs were collected and broken to expose the luminal space. Then, the pieces of h3ACs and h3BCs were incubated with SARS-CoV-2 at multiplicity of infection (MOI) of 0.1 or 1.0 for 2 h.

(B) Representative images for plaque assay using SARS-CoV-2 infected cells at 3 dpi. Dilution factors are shown in the right upper corner. Scale bar, 1 cm.

(C) Plaque assay showing that SARS-CoV-2 actively replicates in h3ACs at 1 dpi. Data are presented as mean ± SEM (n = 2, two plaque assays at each time point). h3ACs at passages 2–3 from two donors and h3BCs at passage 2 from two donors were used in the plaque assays.

(D) qPCR analysis measuring the viral N gene transcripts of SARS-CoV-2 in lysed h3ACs with MOI of 0.1 and 1.0 and in supernatant of h3ACs with MOI of 1.0. Data are presented as mean ± SEM (n = 3, three qPCR assays at each time point). h3ACs at passages 1–3 from three donors and h3BCs at passage 2 from two donors were used in the experiment.

(E) The viability of Vero cells remarkably decreases at 2 days after SARS-CoV-2 infection, whereas that of h3ACs does not significantly change. Data are presented as mean ± SEM (n = 2, three measurements of luminescence at each time point). h3ACs at P0 from one donor were used in the experiment.

organelles such as lamellar bodies, a secretory vesicle containing surfactant proteins (Figures 1B–1D and S1C) (Barkauskas et al., 2013; Van der Velden et al., 2013). Further, HTII-280^{+} hAT2 cells expressing HOPX were also observed in our h3ACs, as well as in alveolar tissue sections (Travaglini et al., 2020) (Figures S1C and S1D). Notably, hAT2 cells started to form a small cluster revealing enriched expression of HTII-280 and F-actin on the luminal side of h3ACs (Figure 1E). During the growth of h3ACs, although the location of HTII-280 expression became varied (Figure S1E), the distribution of F-actin was maintained specifically on the luminal and lateral surfaces of hAT2 cells, consistent with a previous observation in distal embryonic lung epithelium (El-Hashash and Warburton, 2011) (Figure 1F). In contrast, SCRIB (Scribble), which establishes apical-basal polarity (Rodriguez-Boulan and Macara, 2014), was restricted to the basolateral surface of hAT2 cells (Figure 1F). We found that CRB3, which was previously reported to mark an epithelial apical domain, including proximal airway epithelium in lung development (Pocha and Knust, 2013; Szymaniak et al., 2015), was expressed broadly within hAT2 cells (Figures 1E and 1F).

We found that WNT activation was an essential factor for clonal expansion of hAT2 cells, as evidenced by the lack of colony formation in the absence of the WNT activator CHIR99021 in primary culture (Figure S1F). HTII-280^{-} cells were also cultured under conditions supporting 3D cultures of human bronchial (airway) cells (hereafter referred to as h3BCs) that have previously been reported (Sachs et al., 2019). They grew quickly by day 14, with cystic-like structures consisting of a number of airway cell types, including KRT5^{+}TP63^{+} basal cells and SCGB1A1^{+} secretory cells, as previously reported (Figures S1G–S1I) (Sachs et al., 2019). Significantly, our culture condition allowed for long-term expansion of hAT2 cells (up to 10 months), although colony-forming efficiency varied between individual donor samples (Figures 1G and 1H). Importantly, over serial passaging via single-cell dissociation, hAT2s revealed clonal expansion, leading to 3D structures consisting of mature hAT2 cells expressing pro-SFTPC and uptake of Lysotracker following 6 months of continuous culture (Figure 1I). In addition, hAT2 cells cultured for 6 months maintained normal karyotypes (Figure S1J), suggesting chromosome-level genomic stability for this period. Importantly, upon culture in 2D, which promotes the AT1 cell phenotype (Dobbs, 1990), clonally expanded hAT2 cells differentiated into hAT1 cells expressing AGER and AQP5, indicating retention of AT1 cell differentiation capacity in hAT2 cells in our culture condition (Figure 1J). Over long-term culture, we found that colony-forming efficiency was reduced at later passages, with colony size decreasing (Figures 1H and S1K). In 8-month-old 3D cultures, some cells lost the expression of pro-SFTPC, although no expression of airway markers such as TP63 was detected (Figure 1K).

SARS-CoV-2 Infection in h3ACs

We next infected our established h3ACs (and h3BCs) with SARS-CoV-2 (Figure 2A). To this end, we physically and chemically broke h3ACs and h3BCs into pieces to enhance the access of

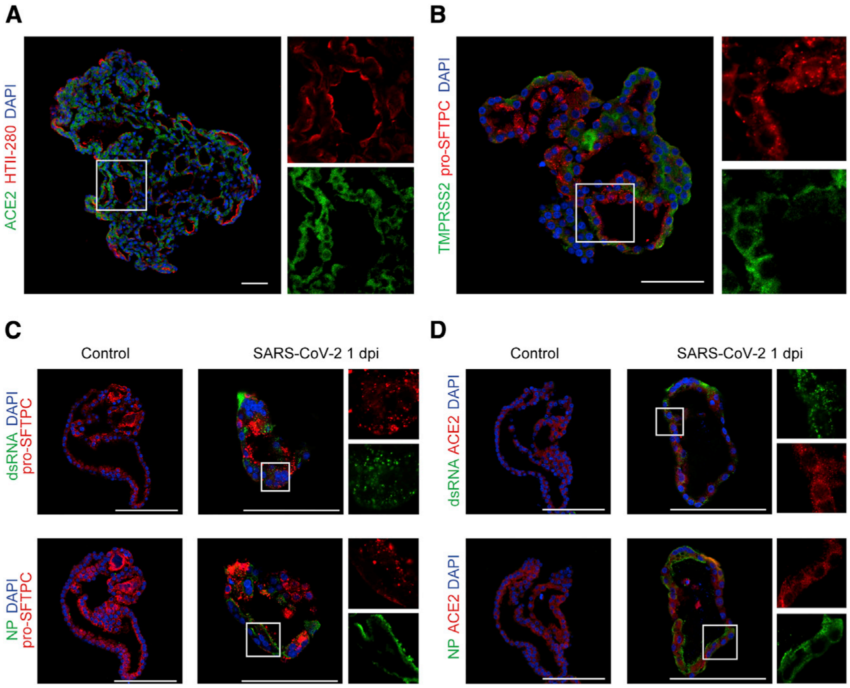

Figure 3. Confocal Imaging of SARS-CoV-2-Infected h3ACs

(A) Representative image of IF staining of ACE2 (green) and HTII-280 (red) in h3ACs. All replicates (n = 5 at passage 1–2 from three donors) of control h3ACs express ACE2 and HTII-280. Scale bar, 50 μm.

(B) Representative image of IF staining of TMPRSS2 (green) and pro-SFTPC (red) in h3ACs. All replicates (n = 5 at passage 1–2 from three donors) of control h3ACs express TMPRSS2 and pro-SFTPC. Scale bar, 50 μm.

(C) Representative images of an infected h3AC at 1 dpi. Viral components (NP or dsRNA; green) are co-stained with pro-SFTPC (red). At 1 dpi, SARS-CoV-2 highly infects h3ACs, which show a punctated pattern of pro-SFTPC. n ≥ 5 replicates of infected h3ACs; P2–P3 from three donors. Scale bar, 50 μm.

(D) Representative images of an infected h3AC expressing ACE2 (red). SARS-CoV-2 are identified by viral dsRNA (green) or NP (green). Viral dsRNA appears punctated. n ≥ 5 replicates of infected h3ACs; P2–P3 from three donors. Scale bar, 50 μm.

SARS-CoV-2 to the apical cell surfaces, followed by viral incubation at a multiplicity of infection (MOI) of 1.0 and 0.1. Viral particles were collected and prepared from a Korean patient (known as KCDC03) who was diagnosed with COVID-19 on January 26, 2020, after traveling to Wuhan, China (Kim et al., 2020b). Vero cells, an interferon-deficient 2D cell line conventionally used as viral host, were also experimentally infected as a positive control, although this was not directly comparable to our 3D infection models due to different technical procedures.

In the plaque-forming assay with MOI of 1.0, infectious viral particles increased to significant titers in h3ACs within the first day post-infection (dpi), suggesting that exponential viral replication occurs <24 h after viral entry in hAT2 cells (Figures 2B and 2C). The increment of viral particles was observed in h3BCs, as reported previously (Suzuki et al., 2020), but their titers were ~100 times lower than h3ACs (Figures 2B and 2C). Infected Vero cells exhibited an even higher SARS-CoV-2 viral burden than h3ACs (Figures 2B and 2C). In line with viral particles, viral RNA levels in the infected h3ACs also highly increased at 1 dpi (Figure 2D). Further, viral transcripts were also detected in the supernatant of h3ACs (Figure 2D), suggesting active secretion of viral transcripts (or particles) from the infected hAT2 cells. In the lower infection experiments with MOI of 0.1, a substantially lower viral burden was observed over the 3 days, suggesting that a fraction of hAT2 cells were likely left uninfected by 3 dpi (Figure 2D). Notably, infected h3ACs did not show substantial cytopathies, such as cell degeneration and syncytium formation (Chu et al., 2020), and maintained cell viability, unlike Vero cells (Figures 2E and S2).

Visualization of SARS-CoV-2 Infection in h3ACs

Immunofluorescence (IF) staining of infected h3ACs revealed widespread expression of ACE2 and TMPRSS2 proteins, which are necessary for SARS-CoV-2 infection (Figures 3A and 3B) (Hoffmann et al., 2020). Despite conventional wisdom, these proteins were not present exclusively in membranes and were also found in the cytoplasm, as reported previously (Hamming et al., 2004; Manna and Caradonna, 2020; Zhou et al., 2020). In infected h3ACs (MOI of 1.0 at 1 dpi), viral components were clearly observed by IF staining of nucleocapsid protein (NP) and double-stranded viral RNA (dsRNA) of SARS-CoV-2. These two viral components were robustly detected in the cytoplasm of hAT2 cells with expression of pro-SFTPC, ACE2, and TMPRSS2 (Figures 3C, 3D, S3A, and S3B). IF staining of infected h3ACs revealed that ~94% of h3ACs (n = 61 out of 65 h3ACs) harbored cells with detectable levels of viral components (Figure S3C). At the level of individual cells, ~61% of cells within individual h3ACs showed evidence of viral components (Figure S3C). However, a larger number of cells may have been infected, as cells with minimal viral components may have fallen below our detection threshold (Discussion). When h3ACs were treated with camostat mesylate, a serine protease inhibitor active against TMPRSS2, SARS-CoV-2 entry and progression were partially blocked, corroborating that the activity of TMPRSS2 is required for viral infection of hAT2 cells (Figure S3D).

To further determine the subcellular consequences of SARS-CoV-2 infection at higher resolution, we performed transmission electron microscopy (TEM) analysis. Compared with uninfected h3ACs (Figure S4), infected h3ACs at 2 dpi with MOI of 1.0 showed viral particles and aggregated proteins that appeared as electron-dense objects (Knoops et al., 2008) in ~51% of cells within four h3ACs explored (Figures 4A–4J; Discussion). Infected hAT2 cells exhibited pathogenic subcellular alterations, such as massive vacuoles, as seen in Zika-virus-infected epithelial cells (Figures 4A and 4F) (Arbour et al., 1999; Monel et al., 2017). Viral particles were either dispersed or encapsulated in a vesicular structure in cytoplasm (Figures 4C, 4D, and 4G). Double-membrane vesicles (DMVs), subcellular structures known as sites of

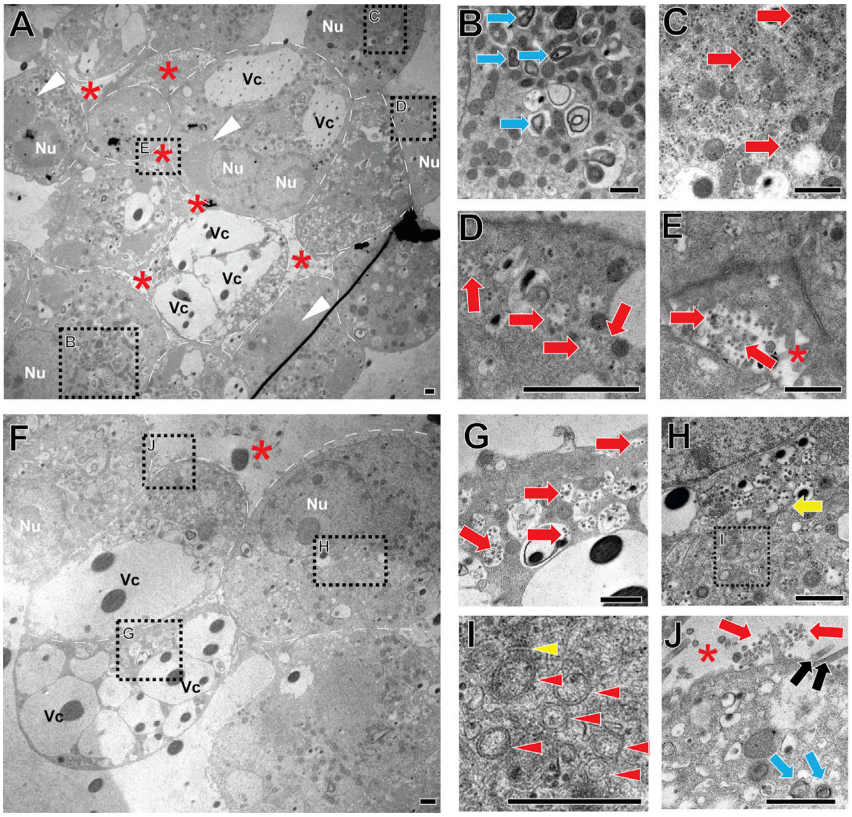

Figure 4. Transmission Electron Microscopic Imaging Analysis of SARS-CoV-2-Infected h3ACs at 2 Days after Infection

(A) Low-magnification representative image of infected h3ACs (n = 10 h3ACs from three donors; P2–P3). Red asterisks, alveolar space; white arrowheads, aggregated viral particles; white dashed line, hAT2 cell membrane. Vc, large pathologic vacuoles; Nu, nucleus.
(B) Lamellar bodies (blue arrow) are observed.
(C) hAT2 cells with a high density of SARS-CoV-2 particles. Viral particles are dispersed in a cytoplasm of the cell (red arrow).
(D) Multiple viral particles included in the vesicular structures (red arrow).
(E) Virus particles secreted to the lumen of the h3AC.
(F) Partial image of another infected h3AC.
(G) Virus particles encapsulated in vesicular structures (red arrow).
(H) Virus-containing vesicles aggregated in the vicinity of ER (yellow arrow).
(I) Double-membrane vesicles (red arrowhead) located near zippered ER (yellow arrowhead).
(J) Viral particles secreted into the lumen of a h3AC (red arrows). Microvilli (black arrow) are shown at the apical side of a hAT2 cell.
Scale bars, 1 μm.

early viral replication, were rarely observed in the vicinity of zippered endoplasmic reticulum (ER) in an infected cell (Figure 4I) (Maier et al., 2013; Ogando et al., 2020). Virus secretion was also observed in the luminal spaces of h3ACs (Figures 4E and 4J).

Intriguingly, a fraction of cells in h3ACs (~19% of cells within the four h3ACs explored) showed much higher viral burdens than other cells (Figure 4C), with as many as 500 copies in the 100-nm section, implying >10,000 SARS-CoV-2 particles in the cell under an assumption of uniform intracellular viral distribution. It remains to be studied whether these viral particles are to be secreted or are competent for infection of other cells. However, if 10% of them are actively released, then the burst size of SARS-CoV-2 from hAT2 cells would be larger than previously suggested for murine hepatitis virus, a viral species of beta coronavirus (Bar-On et al., 2020).

Transcriptome Changes in h3ACs after SARS-CoV-2 Infection

We next explored gene expression changes after SARS-CoV-2 infection in h3ACs. To this end, strand-specific Illumina mRNA libraries were constructed from infected h3ACs with MOI of 1.0 and sequenced (seven samples overall at 0, 1, and 3 dpi with two, three, and two biological replicates, respectively). For comparison, the transcriptome of infected Vero cells was also sequenced.

A set of human genes was differentially expressed among the three time points (Figure 5A), although most genes showed good correlations in gene expression levels (Table S1; Figure S5A). Cytokeratin genes (including KRT16, KRT6A, KRT6B, and KRT6C), genes involved in keratinization (including SPRR1A), and cytoskeleton (including S100A2) and cell-cell adhesion genes (including DSG3) were significantly reduced to ~2%–3% in h3ACs at 3 dpi compared to the levels at 0 dpi (Figures 5A and 5B). Many more genes were upregulated in the infected h3ACs specifically at 3 dpi. In particular, transcription of a broad range of interferon-stimulated genes (ISGs), known to be typically activated by type I and III interferons (Iwasaki et al., 2017), was remarkably increased. These genes include interferon-induced protein genes (such as *IFI6*, *IFI27*, *IFI44*, and *IFI44L*), interferon-induced transmembrane protein genes (such as *IFITM1*), interferon-induced transmembrane proteins with tetratricopeptide repeats genes (*IFIT1*, *IFIT2*, and *IFIT3)*, 2′-5′-oligoadenylate synthetase genes (*OAS1* and *OAS2*), and miscellaneous genes known to be involved in innate cellular immunity (*MX1*, *MX2*, *RSAD2*, and *ISG15*). Expression levels of these genes increased 20 times or higher in h3ACs at 3 dpi compared to at 0 dpi. Many other known ISGs also showed moderate inductions (2–20 times), including *BTS2*, *OAS3*, *HERC5*, *HERC6*, and *USP18*. These ISGs are known to have antiviral functions (Schneider et al., 2014), including (1) inhibition of virus entry (*MX* and *IFITM* genes), (2) inhibition of viral replication and translation (IFIT genes, OAS genes, and *ISG15*, *HERC5*, *HERC6*, and *USP18*), and (3) inhibition of viral egress (*RSAD2* and *BST2*). Of the 20 interferon genes in the human genome, an interferon beta gene (*IFNB1*) and three interferon lambda genes (*IFNL1*, *IFNL2*, and *IFNL3*) showed significant transcriptional induction, although their absolute changes were not substantial (Figure 5C). The surface receptors of interferons were stably expressed in h3AC cells without reference to viral infection (Figure 5C). Downstream signaling genes of the receptors were appreciably upregulated (2–7 times) (e.g., *STAT1*,

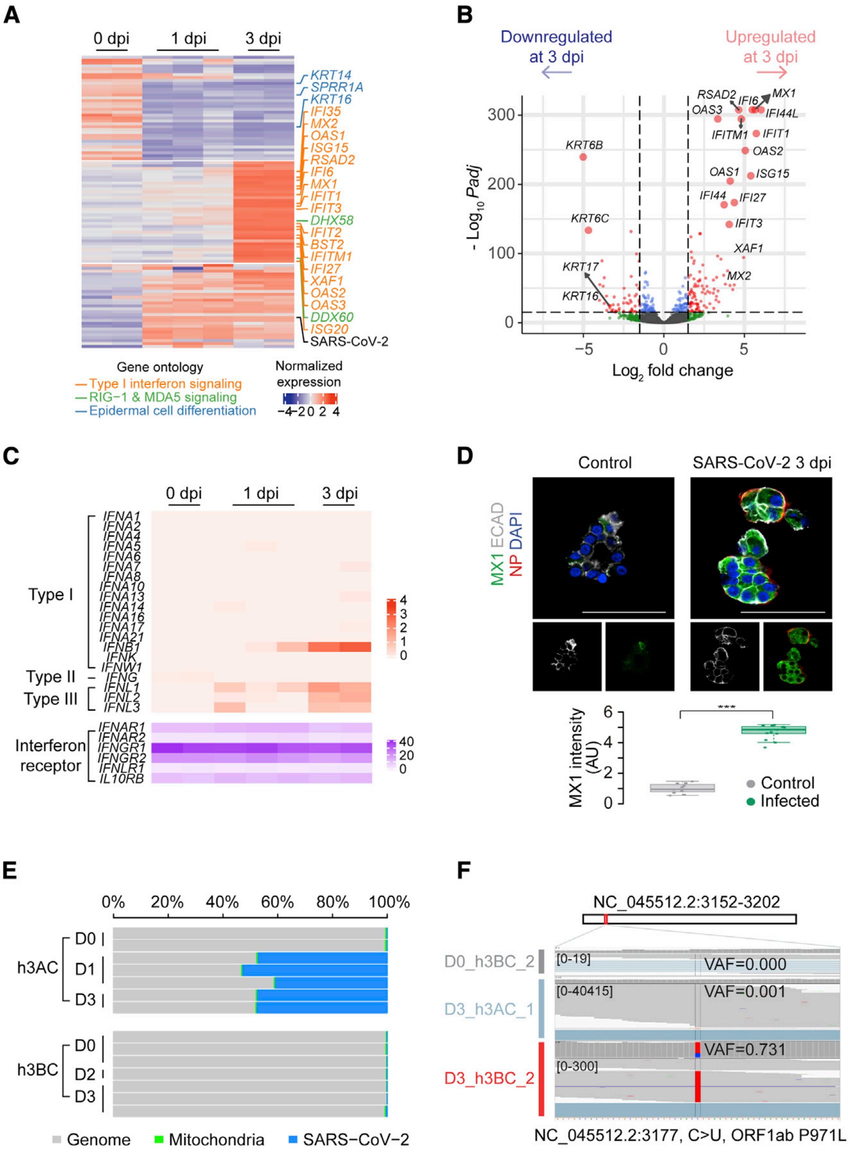

Figure 5. RNA-Sequencing Analyses of Infected h3ACs and h3BCs

(A) Heatmap of the most variable 100 genes among three groups of h3ACs at 0, 1, and 3 dpi.

(B) Volcano plot showing differentially expressed genes between h3ACs at 0 and 3 dpi.

(C) Transcriptional changes of interferon genes in infected h3ACs by transcripts per million (TPM) values.

(D) IF imaging for upregulated MX1 (green). The intensity of MX1 significantly increases in infected h3ACs (p value < 0.001). n = 11 for control and n = 13 for infected h3ACs.

(E) Proportion of viral RNA reads in h3AC and h3BC transcriptomes.

(F) Example of a missense mutation (NC_045512.2: 3,177C > U) detected from a h3BC transcriptome at 3 dpi.

STAT2, and their associated genes, such as *IRF1* and *IRF9*). Of note, *IRF1* is known to be specific to type I interferon responses (Forero et al., 2019), while type I and type III ISGs are generally overlapping (Park and Iwasaki, 2020).

In addition to ISGs, genes in the viral sensing pathway in the cytosol showed increased expression in infected h3ACs at 3 dpi, including *DDX58* (official gene name of RIG-1 [~13 times]), *IFIH1* (also known as MDA5 [~5 times]), *TLR3* (Toll-like receptor 3 [~3 times]), *IRF7* (interferon regulatory factor 7 [~7 times]), and *IL6* (a proinflammatory factor [~4 times at 1 dpi]). Of note, the innate immune response observed was completely autologous, because immune cells are absent in our h3AC system, thus mimicking the very early phase of SARS-CoV-2 alveolar infection. As a validation study, we performed qPCR and IF staining in infected h3ACs (Figures 5D and S5B), which supported the transcriptome changes.

To understand the biological reliability of these upregulated genes, we compared the gene list with transcriptomes produced from the lung tissue of COVID-19 patients (obtained from eight tissues from two COVID-19 pneumonia patients) (Blanco-Melo et al., 2020). Unlike our models, tissue transcriptome showed a substantially higher level of variance presumably caused by the diverse cell-type composition in the tissue (i.e., airway epithelial, immune, mesenchymal, and endothelial cells; Figure S5C). Of the 97 DEGs in our h3AC models, 26.8% were confirmed in infected human tissues (Figure S5D; Table S2).

We then further explored the utility of our h3AC models by comparison with other cellular models. In h3BCs, for example, most of the DEGs observed in h3ACs were not substantially altered (Figures S5E; Table S3). Indeed, only a few genes were transcriptionally altered in h3BCs at 3 dpi (Figure S5F), presumably due to insufficient SARS-CoV-2 infection as mentioned above (Figure 2C). Similarly, 2D cell lines (Emanuel et al., 2020a) established from lung and colon cancers (NCI-H1299 and Caco-2, respectively) showed no evidence of sufficient SARS-CoV-2 infection (Figure S5G). Vero cells (sequenced in this study) showed expression changes in many genes, but transcription of ISGs was not altered, presumably due to the absence of interferon genes in the genome (Figure S5G). Calu-3 cells, a cell line established from lung cancer, showed similar gene expression changes with our h3ACs (Figure S5G). Among these models, our h3ACs showed the highest concordance with human lung tissues (Figure S5H). In addition, prolonged observation (>1 dpi) of viral infection is not feasible with these cell lines, because infected 2D cells are easily suspended from the culture plate.

Expression of Viral Genes

We further analyzed viral RNA from the transcriptomes of infected h3AC and h3BC models. In agreement with the plaque assay (Figures 2B and 2C), the proportion of viral reads in

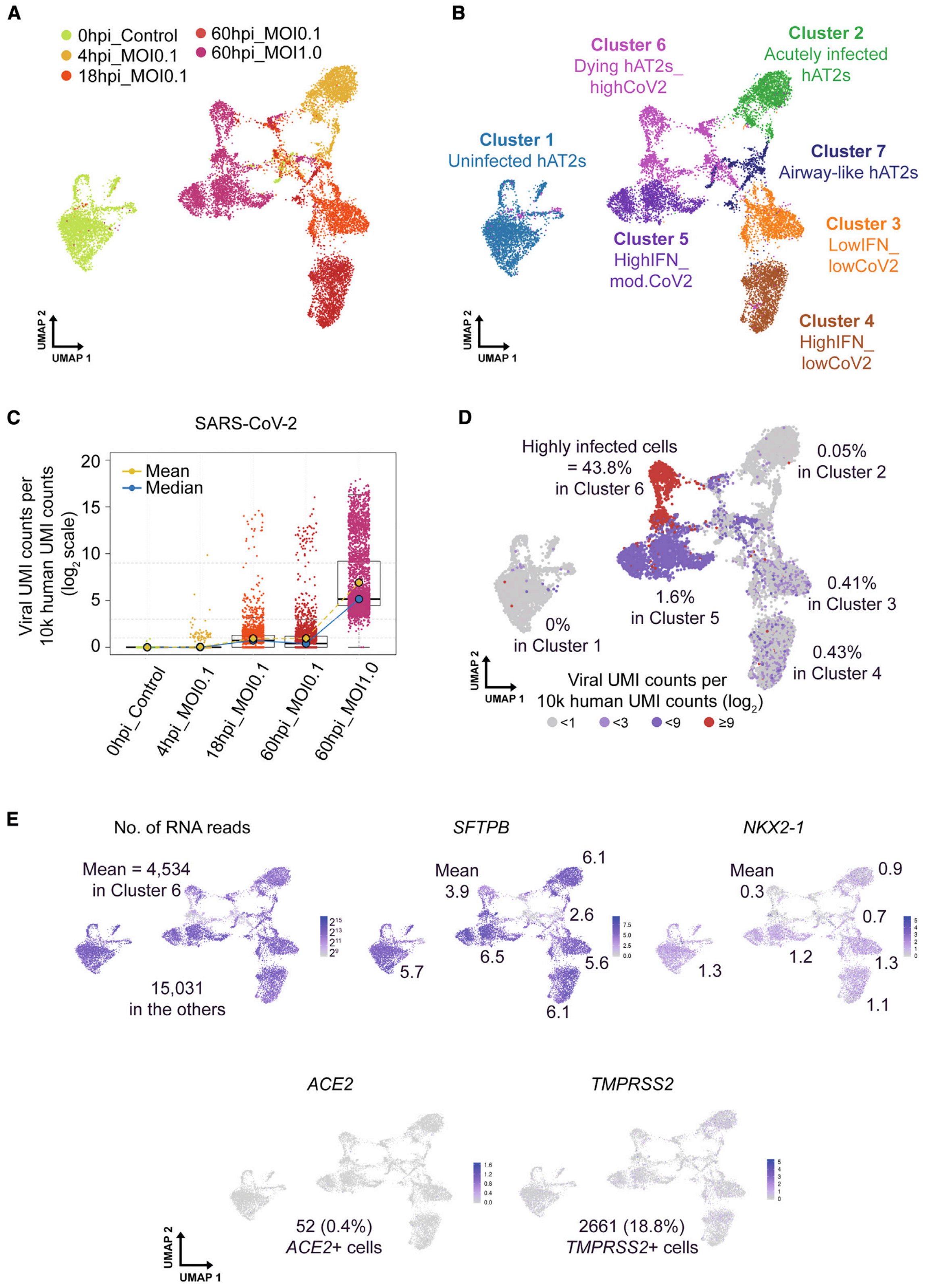

Figure 6. Single-Cell Transcriptome Analysis of Uninfected and Infected h3ACs

(A) Five experimental conditions of the dataset in the UMAP plot. hpi, hours post infection.

(B) Unsupervised UMAP clustering of uninfected and infected h3ACs.

(legend continued on next page)

whole-transcriptome sequences plateaued by 1 dpi in h3ACs (Figure 5E). Approximately 50% of the sequencing reads were mappable to the SARS-CoV-2 genome in h3ACs from 1 dpi, indicating prevailing viral gene expression in infected AT2 cells, as observed in Vero cells (Kim et al., 2020a). In contrast, the proportion of viral reads was less than 0.3% in infected h3BCs until 3 dpi (Figure 5E).

Viral transcripts were not mapped uniformly to the viral genome sequence, but 3′ genomic regions of canonical subgenomic RNA (sgRNA) showed much higher read depths in all samples, consistent with a previous report (Figure S5I) (Kim et al., 2020a). The vast majority of viral RNA sequences were in the orientation of positive-sense RNA strands (Figure S5I; for example, 99.98% versus 0.02% for positive- and negative-sense RNAs, respectively, from h3ACs at 1 dpi). This is in good agreement with the nature of SARS-CoV-2, which is an enveloped, non-segmented, and positive-sense RNA virus. Therefore, its genomic RNA in viral particles and sgRNA transcripts being translated in the cytoplasm should be positive-sense RNAs. Negative-sense RNAs are intermediate templates for replication and/or transcription of positive-sense RNAs.

Cross-comparison of viral RNA sequences obtained from the 11 infected h3ACs (n = 5) and h3BCs (n = 6) revealed 20 viral base substitutions (Table S4). No mutation was at 100% variant allele fraction (VAF) and exclusive to an infection experiment. Instead, sequence alterations showed a broad range of quasispecies heterogeneity in each culture (VAF ranges from 0.1% to 73.1%; Figure 5F), and a large proportion of the mutations (n = 16; 80%) were shared by two or more infected models (by the cut-off threshold of 0.1%). Therefore, we speculate that most of the base changes were originally present in the pool of viral particles before their inoculation to our models. Given the fact that these viral particles were prepared from one of the earliest COVID-19 patients, our finding suggests that mutations can accumulate in the pool of viral genomes in a small number of rounds of viral transmission and appear with dramatic changes in quasispecies abundance. A substantially higher proportion of specific mutations in a sample may suggest a bottleneck in viral entry or stochasticity in viral replication.

Transcriptome Changes at Single-Cell Resolution

To understand transcriptional changes in infected h3ACs at single-cell resolution, we employed a 10X Genomics single-cell RNA sequencing (RNA-seq) platform for h3ACs in five different conditions (uninfected; MOI of 0.1 at 4, 16, and 60 hpi [hours post-infection]; and MOI of 1.0 at 60 hpi). From the five conditions, we captured a total of 14,174 single cells, with 3,266 detectable genes per cell by 13,500 unique molecular identifiers (UMIs) on average. Using UMIs from the host transcripts, we conducted integration (count normalization, feature selection, dimension reduction, and visualization through Uniform Manifold Approximation and Projection [UMAP]) (Figure 6A) and unsupervised clustering of single cells (Figures 6B and S6A) through Seurat and SC3 packages (Kiselev et al., 2019; Stuart et al., 2019). With the exception of cluster 7 (from Seurat), which showed airway-like cells in h3ACs (778 cells from the five conditions; Figure S6B; Discussion), the other six clusters exhibited hAT2 cell features during the course of viral infection. The Seurat clusters consisted of cluster 1 (mostly uninfected cells [3,146 cells]), clusters 2–4 (mostly cells from lower infection experiments at different time points [2,048, 1,932, and 2,092 cells for clusters 2, 3, and 4, respectively], cluster 5 (mostly cells with moderate to high levels of viral transcripts [2,111 cells], and cluster 6 (cells disintegrating and likely close to cell death [2,067 cells]). Generally, cells in MOI of 1.0 and/or the later stage (60 hpi) showed a higher proportion of single cells harboring viral UMIs (Figure 6C). In particular, 99.9% of cells in h3ACs with MOI of 1.0 at 60 hpi showed viral UMIs, confirming our previous imaging analyses that a substantial number of cells in infected h3ACs were infected at 1–3 dpi (Figures 3C, 3D, 4, S6C, and S6D; Discussion). The number of viral transcripts, however, was not uniformly distributed but was enriched in a fraction of cells in each condition (Figure 6C). For example, infected cells in cluster 6 exhibited on average 10.7 times more viral UMI counts than cells in cluster 5 (1,280 versus 119 UMIs, respectively), despite cells in cluster 6 containing relatively lower total UMI counts than cells in other clusters (4,534 versus 17,923 UMIs, respectively; Figures 6D and 6E). When normalized with cellular UMI counts, the proportion of highly infected cells ($\geq 2^9$ viral reads per cell) in cluster 6 was 28 times higher than that in cluster 5 (Figure 6D). Interestingly, the highly infected cells in cluster 6 showed reduced expression of canonical hAT2 marker genes, including *SFTPB* (surfactant protein B) and *NKX2-1* (NK2 homeobox 1) (Figure 6E). Further, we found 8.2% of actively proliferating hAT2 cells (*MKi67*$^+$ h3ACs) in uninfected h3ACs (cluster 1), which was more or less maintained across infection progression in infected h3ACs (cluster 2, 10.6%; cluster 3, 12.8%; cluster 4, 3.6%; cluster 5, 30.3%; and cluster 6, 5.1%) (Figure S6F).

Of note, 52 cells (0.4%) showed *ACE2* transcripts, 2,661 cells (18.8%) expressed *TMPRSS2* transcripts, and 17 cells (0.1%) expressed both in single cells (Figure 6E), despite widespread protein expression (Figures 3A and 3B). These proportions are low at face value but are consistent with a previous observation (Ziegler et al., 2020). Although the previous report also suggested that *ACE2* RNA expression can be stimulated as an infection-mediated response, particularly in human airway cells, such a trend was not observed in our dataset.

Our single-cell clusters illustrated the characteristic transcriptional changes in the course of SARS-CoV-2 infection (Figures 7A and 7B; Table S5). For example, *AQP5* transcription, highly expressed in a subset of hAT2 cells that selectively express Wnt pathway genes and were proposed to be alveolar stem cells (Travaglini et al., 2020), was robust in uninfected h3AC cells (cluster 1) yet downregulated in the other clusters of infected cells. In the acute phase of viral infection (4 hpi; cluster 2), genes responsive to ER stress, such as *HSPA1A*, *HSPA5*, and *HERPUD1*, were specifically upregulated, suggesting that viral infection affects cellular phenotypes from the early hours after cellular

(C) Normalized levels of SARS-CoV-2 viral UMI counts in each experimental condition.

(D) Normalized levels of SARS-CoV-2 viral UMI counts in the UMAP plot. Highly infected cells (viral UMI counts per 10,000 human UMI counts $\geq 2^9$) are mostly enriched in cluster 6.

(E) Expression of genes showing general features of h3ACs in the dataset.

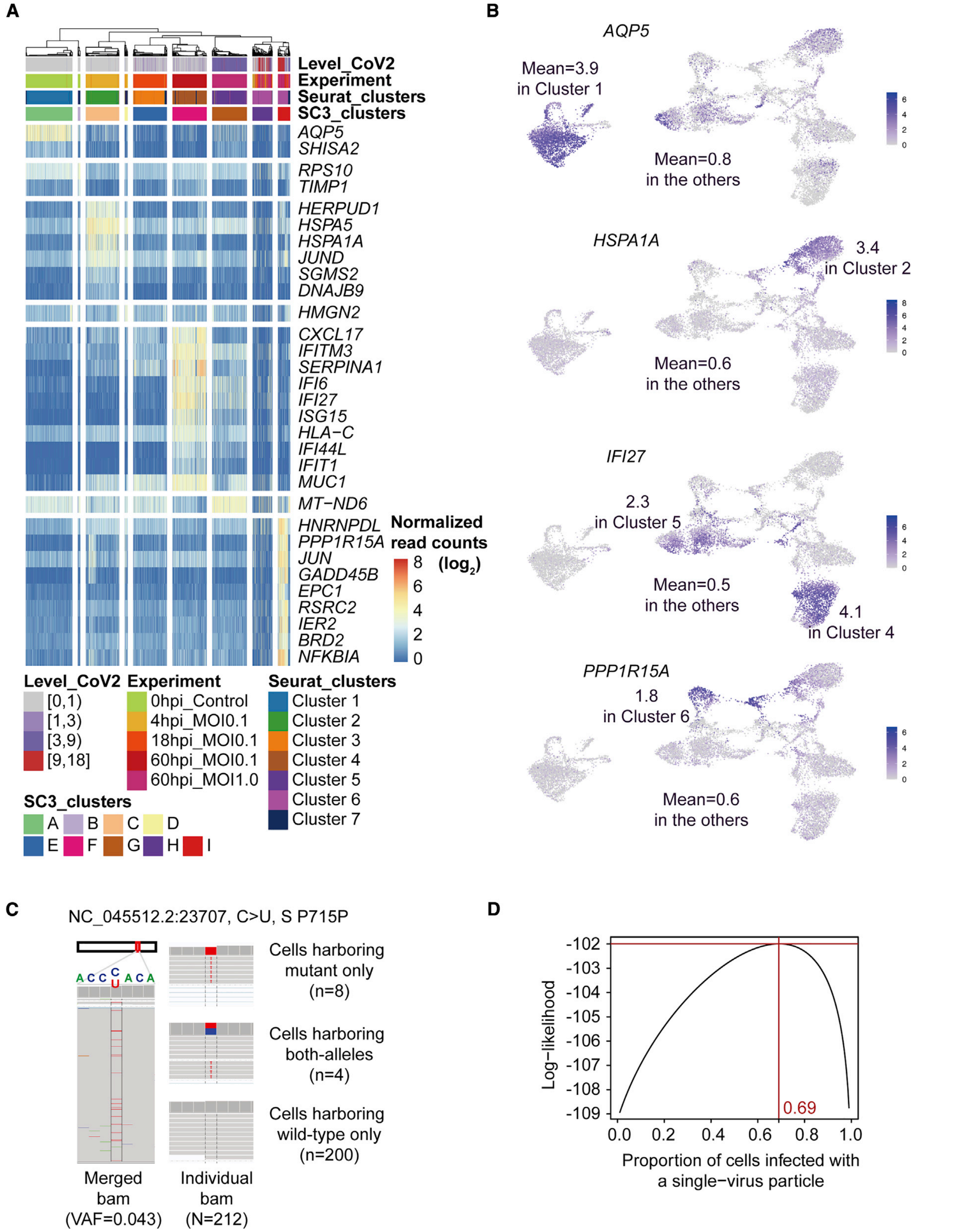

Figure 7. Gene Markers for Each of the Cluster and Estimation of the Number of Viruses Entering a Host Cell

(A) Heatmap showing highly expressed variable genes in each cluster. The most significantly variable genes (≤10) in each cluster are shown. Level_CoV2, log_2 level of SARS-CoV-2 UMI counts per 10,000 human UMI counts; normalized read counts ((log_2)), UMI counts of a certain gene per 10,000 human UMI counts.

(legend continued on next page)

entry. In clusters 4 and 5, hAT2 cells at 60 hpi upregulated ISGs, such as *IFI27* and *IFI6*. In the cluster of disintegrating cells (cluster 6), expression levels of ISGs were mostly reduced. Instead, these cells showed transcriptional induction of apoptosis mediators, such as *PPP1R15A* and *GADD45B*, suggesting an active catastrophic cellular pathway due to extreme viral burdens. Gene Ontology analyses with the variable genes in single-cell transcriptome sequencing mostly highlighted infection-related pathways (Figure S6E).

Effective Entry Number of Viral Particles for Infection of an Alveolar Cell

Finally, we statistically inferred the number of viral particles effectively entering each alveolar cell for infection. Although thousands of viral copies were often observed in infected cells (Figure 4), it is generally not known how many viral particles are necessary for effective infection of an alveolar cell. In an extreme scenario, one viral particle is sufficient. Alternatively, infection may be initiated with the entry of multiple viruses. We tracked the effective viral number of cellular entries using a mutation (NC_045512.2: 23,707C > U; a silent mutation in spike protein) as a viral barcode in the viral quasispecies (Figure 7C). From our sequencing, the mutation was estimated to be present at 4.3% VAF in the initial viral pool for inoculation. If the first scenario dominantly applies, then the infected alveolar cells will be dichotomized (i.e., 95.7% cells with solely wild-type virus and the rest of the cells with solely mutant virus, exclusively). Alternatively, if multiple viruses are effectively entering a cell, then a large proportion of alveolar cells harboring detectable mutant virus will have intermediate VAFs for the mutation. In our single-cell transcriptomes of h3ACs with MOI of 1 infection, 212 cells had at least two independent transcripts for the mutant locus. The majority of cells harboring the mutant allele did not have the wild-type allele present (n = 8 versus 4 for cells showing solely mutant allele versus mixture of the quasispecies, respectively; Figure 7C). In a more sophisticated statistical analysis, infection by single viral entry is estimated to be >2 times more frequent than infection by multiple viral entry (69% versus 31%, respectively; Figure 7D). Our calculation indicates that a single viral particle is mainly responsible for SARS-CoV-2 infection in most hAT2 cells, although multiple viral entry is also possible. It may also reflect viral interference in SARS-CoV-2 alveolar infection.

DISCUSSION

In this study, we have established an experimental condition for feeder-free, long-term, 3D cultures of adult hAT2 cells. We demonstrated that WNT activity is crucial for hAT2 maintenance, consistent with previous studies of murine AT2 cells (Frank et al., 2016; Nabhan et al., 2018; Zacharias et al., 2018). Although our h3ACs may not produce the full cellular spectrum present in the adult alveoli, h3ACs established cellular polarity and maintained functional mature and genomic stable hAT2 cells over multiple passages.

SARS-CoV-2-infected h3ACs showed remarkable cellular and transcriptional changes far more clearly than other models, including h3BCs and 2D Vero cell lines, showing cellular tropism in viral replication and transcription as well as the resultant reaction from the host cell.

SARS-CoV-2 cell entry depends on ACE2 and TMPRSS2 (Hoffmann et al., 2020). Although their protein levels are widespread in hAT2 cells (Hamming et al., 2004; Ren et al., 2006), their transcription is not very active in our single-cell transcriptome analysis of h3ACs, in agreement with recent reports (Zhao et al., 2020; Ziegler et al., 2020). Estimating the proportion of cells harboring low-abundance transcripts with single-cell RNA-seq is generally challenging and frequently results in underestimation.

On the contrary, we observed that single-cell transcriptome sequencing enables more sensitive and scalable detection of infected cells compared to immunostaining and TEM analyses (Figure S6D). We believe that newly infected cells, which include abundant viral transcripts prior to the production of viral protein components, are more likely to be detected by single-cell transcriptome techniques. However, the detection sensitivity is dependent on the burden of viral transcripts in cells as well as the depth of single-cell sequencing.

Through multidimensional analyses of infected h3ACs, we found that (1) viral replication is active during the first day after cellular infection, (2) expression of type I and III interferons begins at 1 dpi, and (3) expression of ISGs follows at 3 dpi. The timing of ISG induction in alveolar cells may be more rapid *in vivo* in concert with exogenous interferons from immune cells. For more physiological understanding, co-culturing SARS-CoV-2-infected h3AC models with immune cells obtained from the same donor would be helpful.

Notably, we identified five states of infected cells across the infection progression with MOI of 0.1 and 1.0 (clusters 2–6). Given that hAT2 cells are more or less homogeneous before infection, this indicates that a distinct switch between cell states occurs during infection. If *in vivo* SARS-CoV-2 infection transforms hAT2 cells into cluster-6-like cells showing a loss of hAT2 identity, the integrity and function of alveoli will be highly compromised. The route toward quantum cellular change is of significant interest to be addressed in future studies.

Although we observe in this study that physiological and molecular changes of alveolar cells appear within 3 dpi, clinical symptoms of COVID-19 sometimes occur after >10 days of viral exposure (Lauer et al., 2020). Several possible explanations could underpin this temporal difference. For example, viral infiltration from the upper respiratory tract to terminal alveoli may take several days, and a substantial proportion of alveolar cells may need to be infected before symptoms develop. In addition, further interactions with immune cells resulting in inflammation may be necessary for symptoms to develop.

From single-cell transcriptome sequences, we observed a subset of cells with distinct transcriptional features (cluster 7), which express lower levels of canonical hAT2 marker genes,

(B) Expression of genes that are represented in the uninfected hAT2 cluster (*AQP5*), the acutely infected hAT2 cluster (*HSPA1A*), lowIFN_lowCoV2 and lowIFN_mod.CoV2 clusters (*IFI27*), and dying hAT2s_highCoV2 (*PPP1R15A*).

(C) Distribution of single-base substitution (variant allele fraction [VAF] = 4.3% in original viral particles) in each cell.

(D) Maximum likelihood estimation for the proportion of cells infected with a single virus.

including *SFTPB* (Figure 6E), but detectable levels of airway marker genes, including *SOX2*, *KRT5*, and *TP63* (Figure S6B). Some of these cells harbored SARS-CoV-2 transcripts, but expression of marker genes was not highly affected. We speculate that these cells are hAT2 cells with pathologic phenotypes of alveolar bronchiolization and/or basal-like hAT2 cells, which are stochastically observed in chronic lung diseases (Jensen-Taubman et al., 1998; Xu et al., 2016). Given that the h3ACs used for SARS-CoV-2 infection were established from hAT2 cells isolated from adjacent normal counterparts of lung cancer and/or idiopathic pulmonary fibrosis (IPF), it is likely that this transcriptional feature reflects the cellular status of original tissues rather than a virus-associated phenotype.

In summary, our study highlights the advantages of h3AC models to elucidate the pathogenesis of SARS-CoV-2 infection in alveolar stem cells. Our data will be a great resource for the biomedical community for deeper characterization of viral disease. We believe that our models will enable more accurate and sophisticated analyses in the near future, especially for studying the response to viral infection within vulnerable groups with aged or diseased lungs, providing an opportunity to elucidate individual patient responses to viral infection. Furthermore, our models can be combined with other techniques, such as co-culture experiments with immune cells and *in vitro* screening of antiviral agents. We believe that our models are also applicable for the study of basic biology and other diseases in hAT2 cells.

Limitations of Study

Our h3AC models were established from lung tissue specimens collected from patients with heterogeneous clinical histories and genetic backgrounds. These confounding factors were not thoroughly controlled in this study. Due to the lack of immune cells in our system, molecular interactions between hAT2 cells and immune cells could not be investigated in this study. Using h3AC models from additional donors and introducing immune cells into the culture system will likely advance our understanding of the pathogenesis of SARS-CoV-2 infection.

STAR★METHODS

Detailed methods are provided in the online version of this paper and include the following:

- KEY RESOURCES TABLE
- RESOURCE AVAILABILITY
  - Lead Contact
  - Materials Availability
  - Data and Code Availability
- EXPERIMENT MODEL AND SUBJECT DETAILS
  - Human Tissues
  - Virus particle preparation for infection
- METHOD DETAILS
  - Human lung tissue dissociation and flow cytometry
  - *In vitro* 3D culture and passage
  - Karyotype of *in vitro* 6 months grown h3ACs
  - Assessment of lung lineage transcripts by qRT-PCR
  - Differentiation of hAT2 cells into hAT1 cells in 2D culture
  - SARS-CoV-2 infection of 3D culture cells and Vero cells
  - Viral RNA extraction
  - Cellular total RNA extraction
  - Viral RNA copy number calculation with qRT-PCR
  - Validation of upregulated genes with qRT-PCR
  - Live virus titer calculation with plaque assay
  - Viability and cytotoxicity test
  - Immunofluorescence staining of paraffin-embedded h3ACs and Lysotracker
  - Immunofluorescence staining of infected h3ACs with cryo-section
  - Immunofluorescence staining of whole-mount h3ACs
  - Immunofluorescence staining of hAT1-like cells
  - Transmission electron microscopy
  - Bulk RNA sequencing and data processing
  - Single-cell RNA sequencing and data processing
  - Statistical inference on the effective number of viral entry
- QUANTIFICATION AND STATISTICAL ANALYSIS

SUPPLEMENTAL INFORMATION

Supplemental Information can be found online at https://doi.org/10.1016/j.stem.2020.10.004.

ACKNOWLEDGMENTS

We thank Jinwook Choi (Wellcome-MRC Cambridge Stem Cell Institute), Sam M. Janes, Rob Hynds, Sarah Clarke (University College London), Yong Man Han, Dae-Sik Lim, Eui-Cheol Shin, Heung Kyu Lee, Su-Hyung Park, Jeong Seok Lee, Jaeoh Park, Myungsuk Choi (KAIST), Jung-Ki Yoon (Seoul National University Hospital), Yoon Ho Kim (Seoul National University Cancer Research Institute), Junghwan Park (Ministry of Health and Welfare), and Jong-Yeon Shin (Macrogen) for valuable discussion, productive comments, and technical help. We thank Irina Pshenichnaya (Histology), Peter Humphreys (Imaging), Simon McCallum (Flow Cytometry, Cambridge NIHR BRC Cell Phenotyping Hub), and Cambridge Stem Cell Institute core facilities for technical assistance. This work was supported by the Suh Kyungbae Foundation (SUHF-18010082 to Y.S.J.); the National Research Foundation of Korea (for Brain Pool Program NRF-2019H1D3A2A02061168 to Y.S.J. and S.Y.K. and Leading Researcher Program NRF-2020R1A3B2078973 to Y.S.J.); the Research of Korea Centers for Disease Control and Prevention (2019-NI069-00 to Y.-I.J.); the Institute for Basic Science (IBS-R025-D1 to G.Y.K.) funded by Ministry of Science and ICT of Korea; the Korea Health Technology R&D Project through the Korea Health Industry Development Institute (KHIDI), funded by the Ministry of Health & Welfare, Republic of Korea (HI14C1277 to Y.S.J. and Y.T.K.); the Research Program 2020 funded by Seoul National University College of Medicine Research Foundation (800-20200516 to Y.T.K.); and European Research Council starting grant 679411. J.-H.L. is supported by Wellcome and the Royal Society (107633/Z/15/Z). K.V.E. is supported by Biotechnology and Biological Sciences Research Council Industrial CASE (BBSRC iCASE) studentships (BB/R505328/1). Y.S.J. is supported by Human Frontier Science Program research grant RGY0071/2018.

AUTHOR CONTRIBUTIONS

Y.S.J. and J.-H.L. conceptualized the study; K.V.E. and J.-H.L. performed hAT2 3D culture optimization; K.V.E., J.Y., T.K., and J.-H.L. established 3D cultures; K.J.N., M.F., K.T.M., K.S.-P., and Y.T.K. prepared specimens; J.H.K., J.Y., T.K., Y.-I.J., and B.-S.C. performed infections; K.V.E., S.P.H., and G.Y.K. performed confocal microscopy; Y.H., T.K., and H.M.K. performed electron microscopy; Seon Young Kim performed karyotyping; J.Y., T.K., K.Y., Su Yeon Kim, and Y.S.J. performed transcriptome analysis; Su Yeon Kim performed statistical analysis; J.Y., T.K., K.V.E., T.B., Su Yeon Kim., G.Y.K.,

Y.S.J., and J.-H.L. wrote the manuscript; all authors revised the manuscript; and Y.S.J. and J.-H.L. supervised the study.

DECLARATION OF INTERESTS

Y.S.J. is a founder and CEO of GENOME INSIGHT.

Received: June 10, 2020
Revised: August 27, 2020
Accepted: October 13, 2020
Published: October 21, 2020

REFERENCES

Adams, T.S., Schupp, J.C., Poli, S., Ayaub, E.A., Neumark, N., Ahangari, F., Chu, S.G., Raby, B.A., DeIuliis, G., Januszyk, M., et al. (2020). Single-cell RNA-seq reveals ectopic and aberrant lung-resident cell populations in idiopathic pulmonary fibrosis. Sci. Adv. *6*, eaba1983.

Andersen, K.G., Rambaut, A., Lipkin, W.I., Holmes, E.C., and Garry, R.F. (2020). The proximal origin of SARS-CoV-2. Nat. Med. *26*, 450–452.

Arbour, N., Côté, G., Lachance, C., Tardieu, M., Cashman, N.R., and Talbot, P.J. (1999). Acute and persistent infection of human neural cell lines by human coronavirus OC43. J. Virol. *73*, 3338–3350.

Bao, L., Deng, W., Huang, B., Gao, H., Liu, J., Ren, L., Wei, Q., Yu, P., Xu, Y., Qi, F., et al. (2020). The pathogenicity of SARS-CoV-2 in hACE2 transgenic mice. Nature *583*, 830–833.

Bar-On, Y.M., Flamholz, A., Phillips, R., and Milo, R. (2020). SARS-CoV-2 (COVID-19) by the numbers. eLife *9*, e57309.

Barkauskas, C.E., Cronce, M.J., Rackley, C.R., Bowie, E.J., Keene, D.R., Stripp, B.R., Randell, S.H., Noble, P.W., and Hogan, B.L.M. (2013). Type 2 alveolar cells are stem cells in adult lung. J. Clin. Invest. *123*, 3025–3036.

Bartfeld, S., Bayram, T., van de Wetering, M., Huch, M., Begthel, H., Kujala, P., Vries, R., Peters, P.J., and Clevers, H. (2015). In vitro expansion of human gastric epithelial stem cells and their responses to bacterial infection. Gastroenterology *148*, 126–136 e126.

Blanco-Melo, D., Nilsson-Payant, B.E., Liu, W.C., Uhl, S., Hoagland, D., Moller, R., Jordan, T.X., Oishi, K., Panis, M., Sachs, D., et al. (2020). Imbalanced host response to SARS-CoV-2 drives development of COVID-19. Cell *181*, 1036–1045 e1039.

Blighe, K., Rana, S., and Lewis, M. (2018). EnhancedVolcano: publication-ready volcano plots with enhanced colouring and labeling, https://github.com/kevinblighe/EnhancedVolcano.

Chu, H., Chan, J.F.-W., Yuen, T.T.-T., Shuai, H., Yuan, S., Wang, Y., Hu, B., Yip, C.C.-Y., Tsang, J.O.-L., Huang, X., et al. (2020). Comparative tropism, replication kinetics, and cell damage profiling of SARS-CoV-2 and SARS-CoV with implications for clinical manifestations, transmissibility, and laboratory studies of COVID-19: an observational study. Lancet Microbe *1*, e14–e23.

Dobbs, L.G. (1990). Isolation and culture of alveolar type II cells. Am. J. Physiol. *258*, L134–L147.

Dobin, A., Davis, C.A., Schlesinger, F., Drenkow, J., Zaleski, C., Jha, S., Batut, P., Chaisson, M., and Gingeras, T.R. (2013). STAR: ultrafast universal RNA-seq aligner. Bioinformatics *29*, 15–21.

El-Hashash, A.H., and Warburton, D. (2011). Cell polarity and spindle orientation in the distal epithelium of embryonic lung. Dev. Dyn. *240*, 441–445.

Elbadawi, M., and Efferth, T. (2020). Organoids of human airways to study infectivity and cytopathy of SARS-CoV-2. Lancet Respir. Med. *8*, E55–E56.

Emanuel, W., Kirstin, M., Vedran, F., Asija, D., Theresa, G.L., Roberto, A., Filippos, K., David, K., Salah, A., Christopher, B., et al. (2020a). Bulk and single-cell gene expression profiling of SARS-CoV-2 infected human cell lines identifies molecular targets for therapeutic intervention. bioRxiv. https://doi.org/10.1101/2020.05.05.079194.

Fatehullah, A., Tan, S.H., and Barker, N. (2016). Organoids as an in vitro model of human development and disease. Nat. Cell Biol. *18*, 246–254.

Forero, A., Ozarkar, S., Li, H., Lee, C.H., Hemann, E.A., Nadjsombati, M.S., Hendricks, M.R., So, L., Green, R., Roy, C.N., et al. (2019). Differential activation of the transcription factor IRF1 underlies the distinct immune responses elicited by type i and type III interferons. Immunity *51*, 451–464.e456.

Forster, P., Forster, L., Renfrew, C., and Forster, M. (2020). Phylogenetic network analysis of SARS-CoV-2 genomes. Proc. Natl. Acad. Sci. USA *117*, 9241–9243.

Frank, D.B., Peng, T., Zepp, J.A., Snitow, M., Vincent, T.L., Penkala, I.J., Cui, Z., Herriges, M.J., Morley, M.P., Zhou, S., et al. (2016). Emergence of a wave of Wnt signaling that regulates lung alveologenesis by controlling epithelial self-renewal and differentiation. Cell Rep. *17*, 2312–2325.

Fujii, M., Matano, M., Toshimitsu, K., Takano, A., Mikami, Y., Nishikori, S., Sugimoto, S., and Sato, T. (2018). Human intestinal organoids maintain self-renewal capacity and cellular diversity in niche-inspired culture condition. Cell Stem Cell *23*, 787–793.e786.

Gonzalez, R.F., Allen, L., Gonzales, L., Ballard, P.L., and Dobbs, L.G. (2010). HTII-280, a biomarker specific to the apical plasma membrane of human lung alveolar type II cells. J. Histochem. Cytochem. *58*, 891–901.

Gu, Z., Eils, R., and Schlesner, M. (2016). Complex heatmaps reveal patterns and correlations in multidimensional genomic data. Bioinformatics *32*, 2847–2849.

Habermann, A.C., Gutierrez, A.J., Bui, L.T., Yahn, S.L., Winters, N.I., Calvi, C.L., Peter, L., Chung, M.-I., Taylor, C.J., Jetter, C., et al. (2020). Single-cell RNA sequencing reveals profibrotic roles of distinct epithelial and mesenchymal lineages in pulmonary fibrosis. Sci. Adv. *6*, eaba1972.

Hamming, I., Timens, W., Bulthuis, M.L., Lely, A.T., Navis, G., and van Goor, H. (2004). Tissue distribution of ACE2 protein, the functional receptor for SARS coronavirus. A first step in understanding SARS pathogenesis. J. Pathol. *203*, 631–637.

Heo, I., Dutta, D., Schaefer, D.A., Iakobachvili, N., Artegiani, B., Sachs, N., Boonekamp, K.E., Bowden, G., Hendrickx, A.P.A., Willems, R.J.L., et al. (2018). Modelling Cryptosporidium infection in human small intestinal and lung organoids. Nat. Microbiol. *3*, 814–823.

Hoffmann, M., Kleine-Weber, H., Schroeder, S., Kruger, N., Herrler, T., Erichsen, S., Schiergens, T.S., Herrler, G., Wu, N.H., Nitsche, A., et al. (2020). SARS-CoV-2 cell entry depends on ACE2 and TMPRSS2 and is blocked by a clinically proven protease inhibitor. Cell *181*, 271–280.e278.

Huang, J., Hume, A.J., Abo, K.M., Werder, R.B., Villacorta-Martin, C., Alysandratos, K.D., Beermann, M.L., Simone-Roach, C., Lindstrom-Vautrin, J., Olejnik, J., et al. (2020). SARS-CoV-2 infection of pluripotent stem cell-derived human lung alveolar type 2 cells elicits a rapid epithelial-intrinsic inflammatory response. Cell Stem Cell. Published online September 18, 2020. https://doi.org/10.1101/2020.06.30.175695.

Iwasaki, A., Foxman, E.F., and Molony, R.D. (2017). Early local immune defences in the respiratory tract. Nat. Rev. Immunol. *17*, 7–20.

Jacob, F., Pather, S.R., Huang, W.K., Zhang, F., Wong, S.Z.H., Zhou, H., Cubitt, B., Fan, W., Chen, C.Z., Xu, M., et al. (2020). Human pluripotent stem cell-derived neural cells and brain organoids reveal SARS-CoV-2 neurotropism predominates in choroid plexus epithelium. Cell Stem Cell. Published online September 21, 2020. https://doi.org/10.1016/j.stem.2020.09.016.

Jensen-Taubman, S.M., Steinberg, S.M., and Linnoila, R.I. (1998). Bronchiolization of the alveoli in lung cancer: pathology, patterns of differentiation and oncogene expression. Int. J. Cancer *75*, 489–496.

Kathiriya, J.J., Wang, C., Brumwell, A., Cassandras, M., Le Saux, C., Wolters, P., Matthay, M., Chapman, H.A., and Peng, T. (2020). Human alveolar type 2 epithelium transdifferentiates into metaplastic KRT5+ basal cells during alveolar repair. bioRxiv. https://doi.org/10.1101/2020.06.06.136713.

Kim, D., Lee, J.Y., Yang, J.S., Kim, J.W., Kim, V.N., and Chang, H. (2020a). The architecture of SARS-CoV-2 transcriptome. Cell *181*, 914–921.e910.

Kim, J.M., Chung, Y.S., Jo, H.J., Lee, N.J., Kim, M.S., Woo, S.H., Park, S., Kim, J.W., Kim, H.M., and Han, M.G. (2020b). Identification of coronavirus isolated from a patient in Korea with COVID-19. Osong Public Health Res. Perspect. *11*, 3–7.

Kim, Y.I., Kim, S.G., Kim, S.M., Kim, E.H., Park, S.J., Yu, K.M., Chang, J.H., Kim, E.J., Lee, S., Casel, M.A.B., et al. (2020c). Infection and rapid transmission of SARS-CoV-2 in ferrets. Cell Host Microbe *27*, 704–709.e702.

Kiselev, V.Y., Andrews, T.S., and Hemberg, M. (2019). Challenges in unsupervised clustering of single-cell RNA-seq data. Nat. Rev. Genet. *20*, 273–282.

Knoops, K., Kikkert, M., van den Worm, S.H., Zevenhoven-Dobbe, J.C., van der Meer, Y., Koster, A.J., Mommaas, A.M., and Snijder, E.J. (2008). SARS-coronavirus replication is supported by a reticulovesicular network of modified endoplasmic reticulum. PLoS Biol. *6*, e226.

Koboldt, D.C., Zhang, Q., Larson, D.E., Shen, D., McLellan, M.D., Lin, L., Miller, C.A., Mardis, E.R., Ding, L., and Wilson, R.K. (2012). VarScan 2: somatic mutation and copy number alteration discovery in cancer by exome sequencing. Genome Res. *22*, 568–576.

Kuleshov, M.V., Jones, M.R., Rouillard, A.D., Fernandez, N.F., Duan, Q., Wang, Z., Koplev, S., Jenkins, S.L., Jagodnik, K.M., Lachmann, A., et al. (2016). Enrichr: a comprehensive gene set enrichment analysis web server 2016 update. Nucleic Acids Res. *44* (W1), W90-7.

Lamers, M.M., Beumer, J., van der Vaart, J., Knoops, K., Puschhof, J., Breugem, T.I., Ravelli, R.B.G., Paul van Schayck, J., Mykytyn, A.Z., Duimel, H.Q., et al. (2020). SARS-CoV-2 productively infects human gut enterocytes. Science *369*, 50–54.

Lauer, S.A., Grantz, K.H., Bi, Q., Jones, F.K., Zheng, Q., Meredith, H.R., Azman, A.S., Reich, N.G., and Lessler, J. (2020). The incubation period of coronavirus disease 2019 (COVID-19) from publicly reported confirmed cases: estimation and application. Ann. Intern. Med. *172*, 577–582.

Lee, J.H., Bhang, D.H., Beede, A., Huang, T.L., Stripp, B.R., Bloch, K.D., Wagers, A.J., Tseng, Y.H., Ryeom, S., and Kim, C.F. (2014). Lung stem cell differentiation in mice directed by endothelial cells via a BMP4-NFATc1-thrombospondin-1 axis. Cell *156*, 440–455.

Li, B., and Dewey, C.N. (2011). RSEM: accurate transcript quantification from RNA-seq data with or without a reference genome. BMC Bioinformatics *12*, 323.

Li, H., Handsaker, B., Wysoker, A., Fennell, T., Ruan, J., Homer, N., Marth, G., Abecasis, G., and Durbin, R.; 1000 Genome Project Data Processing Subgroup (2009). The Sequence Alignment/Map format and SAMtools. Bioinformatics *25*, 2078–2079.

Love, M.I., Huber, W., and Anders, S. (2014). Moderated estimation of fold change and dispersion for RNA-seq data with DESeq2. Genome Biol. *15*, 550.

Maier, H.J., Hawes, P.C., Cottam, E.M., Mantell, J., Verkade, P., Monaghan, P., Wileman, T., and Britton, P. (2013). Infectious bronchitis virus generates spherules from zippered endoplasmic reticulum membranes. MBio *4*, e00801–e00813.

Manna, V.J., and Caradonna, S.J. (2020). The heterogeneous nature of the Coronavirus receptor, angiotensin-converting enzyme 2 (ACE2) in differentiating airway epithelia. bioRxiv. https://doi.org/10.1101/2020.07.09.190074.

Monel, B., Compton, A.A., Bruel, T., Amraoui, S., Burlaud-Gaillard, J., Roy, N., Guivel-Benhassine, F., Porrot, F., Génin, P., Meertens, L., et al. (2017). Zika virus induces massive cytoplasmic vacuolization and paraptosis-like death in infected cells. EMBO J. *36*, 1653–1668.

Monteil, V., Kwon, H., Prado, P., Hagelkruys, A., Wimmer, R.A., Stahl, M., Leopoldi, A., Garreta, E., Hurtado Del Pozo, C., Prosper, F., et al. (2020). Inhibition of SARS-CoV-2 infections in engineered human tissues using clinical-grade soluble human ACE2. Cell *181*, 905–913.e907.

Muus, C., Luecken, M.D., Eraslan, G., Waghray, A., Heimberg, G., Sikkema, L., Kobayashi, Y., Vaishnav, E.D., Subramanian, A., Smilie, C., et al. (2020). Integrated analyses of single-cell atlases reveal age, gender, and smoking status associations with cell type-specific expression of mediators of SARS-CoV-2 viral entry and highlights inflammatory programs in putative target cells. bioRxiv. https://doi.org/10.1101/2020.04.19.049254.

Nabhan, A.N., Brownfield, D.G., Harbury, P.B., Krasnow, M.A., and Desai, T.J. (2018). Single-cell Wnt signaling niches maintain stemness of alveolar type 2 cells. Science *359*, 1118–1123.

Nikolić, M.Z., Caritg, O., Jeng, Q., Johnson, J.A., Sun, D., Howell, K.J., Brady, J.L., Laresgoiti, U., Allen, G., Butler, R., et al. (2017). Human embryonic lung epithelial tips are multipotent progenitors that can be expanded in vitro as long-term self-renewing organoids. eLife *6*, e26575.

Ogando, N.S., Dalebout, T.J., Zevenhoven-Dobbe, J.C., Limpens, R.W., van der Meer, Y., Caly, L., Druce, J., de Vries, J.J.C., Kikkert, M., Bárcena, M., et al. (2020). SARS-coronavirus-2 replication in Vero E6 cells: replication kinetics, rapid adaptation and cytopathology. bioRxiv. https://doi.org/10.1101/2020.04.20.049924.

Park, A., and Iwasaki, A. (2020). Type I and type III interferons: induction, signaling, evasion, and application to combat COVID-19. Cell Host Microbe *27*, 870–878.

Pfaffl, M.W. (2001). A new mathematical model for relative quantification in real-time RT-PCR. Nucleic Acids Res. *29*, e45.

Pocha, S.M., and Knust, E. (2013). Complexities of Crumbs function and regulation in tissue morphogenesis. Curr. Biol. *23*, R289–R293.

Ramani, A., Müller, L., Ostermann, P.N., Gabriel, E., Abida-Islam, P., Müller-Schiffmann, A., Mariappan, A., Goureau, O., Gruell, H., Walker, A., et al. (2020). SARS-CoV-2 targets neurons of 3D human brain organoids. EMBO J. *39*, e106230.

Ren, X., Glende, J., Al-Falah, M., de Vries, V., Schwegmann-Wessels, C., Qu, X., Tan, L., Tschernig, T., Deng, H., Naim, H.Y., and Herrler, G. (2006). Analysis of ACE2 in polarized epithelial cells: surface expression and function as receptor for severe acute respiratory syndrome-associated coronavirus. J. Gen. Virol. *87*, 1691–1695.

Robinson, J.T., Thorvaldsdóttir, H., Winckler, W., Guttman, M., Lander, E.S., Getz, G., and Mesirov, J.P. (2011). Integrative genomics viewer. Nat. Biotechnol. *29*, 24–26.

Rodriguez-Boulan, E., and Macara, I.G. (2014). Organization and execution of the epithelial polarity programme. Nat. Rev. Mol. Cell Biol. *15*, 225–242.

Sachs, N., Papaspyropoulos, A., Zomer-van Ommen, D.D., Heo, I., Böttinger, L., Klay, D., Weeber, F., Huelsz-Prince, G., Iakobachvili, N., Amatngalim, G.D., et al. (2019). Long-term expanding human airway organoids for disease modeling. EMBO J. *38*, 38.

Saunders, C.T., Wong, W.S., Swamy, S., Becq, J., Murray, L.J., and Cheetham, R.K. (2012). Strelka: accurate somatic small-variant calling from sequenced tumor-normal sample pairs. Bioinformatics *28*, 1811–1817.

Schneider, W.M., Chevillotte, M.D., and Rice, C.M. (2014). Interferon-stimulated genes: a complex web of host defenses. Annu. Rev. Immunol. *32*, 513–545.

Shang, J., Ye, G., Shi, K., Wan, Y., Luo, C., Aihara, H., Geng, Q., Auerbach, A., and Li, F. (2020). Structural basis of receptor recognition by SARS-CoV-2. Nature *581*, 221–224.

Sia, S.F., Yan, L.M., Chin, A.W.H., Fung, K., Choy, K.T., Wong, A.Y.L., Kaewpreedee, P., Perera, R.A.P.M., Poon, L.L.M., Nicholls, J.M., et al. (2020). Pathogenesis and transmission of SARS-CoV-2 in golden hamsters. Nature *583*, 834–838.

Stuart, T., Butler, A., Hoffman, P., Hafemeister, C., Papalexi, E., Mauck, W.M., 3rd, Hao, Y., Stoeckius, M., Smibert, P., and Satija, R. (2019). Comprehensive integration of single-cell data. Cell *177*, 1888–1902.e1821.

Suzuki, T., Itoh, Y., Sakai, Y., Saito, A., Okuzaki, D., Motooka, D., Minami, S., Kobayashi, T., Yamamoto, T., Okamoto, T., et al. (2020). Generation of human bronchial organoids for SARS-CoV-2 research. bioRxiv. https://doi.org/10.1101/2020.05.25.115600.

Szymaniak, A.D., Mahoney, J.E., Cardoso, W.V., and Varelas, X. (2015). Crumbs3-Mediated Polarity Directs Airway Epithelial Cell Fate through the Hippo Pathway Effector Yap. Dev. Cell *34*, 283–296.

Travaglini, K.J., Nabhan, A.N., Penland, L., Sinha, R., Gillich, A., Sit, R.V., Chang, S., Conley, S.D., Mori, Y., Seita, J., et al. (2020). A molecular cell atlas of the human lung from single cell RNA sequencing. bioRxiv. https://doi.org/10.1101/742320.

Van der Velden, J.L., Bertoncello, I., and McQualter, J.L. (2013). LysoTracker is a marker of differentiated alveolar type II cells. Respir. Res. *14*, 123.

Walls, A.C., Park, Y.J., Tortorici, M.A., Wall, A., McGuire, A.T., and Veesler, D. (2020). Structure, function, and antigenicity of the SARS-CoV-2 spike glycoprotein. Cell *181*, 281–292.e286.

Wang, Q., Zhang, Y., Wu, L., Niu, S., Song, C., Zhang, Z., Lu, G., Qiao, C., Hu, Y., Yuen, K.Y., et al. (2020). Structural and functional basis of SARS-CoV-2 entry by using human ACE2. Cell *181*, 894–904 e899.

Wölfel, R., Corman, V.M., Guggemos, W., Seilmaier, M., Zange, S., Müller, M.A., Niemeyer, D., Jones, T.C., Vollmar, P., Rothe, C., et al. (2020). Virological assessment of hospitalized patients with COVID-2019. Nature *581*, 465–469.

Wu, F., Zhao, S., Yu, B., Chen, Y.M., Wang, W., Song, Z.G., Hu, Y., Tao, Z.W., Tian, J.H., Pei, Y.Y., et al. (2020). A new coronavirus associated with human respiratory disease in China. Nature *579*, 265–269.

Xu, Y., Mizuno, T., Sridharan, A., Du, Y., Guo, M., Tang, J., Wikenheiser-Brokamp, K.A., Perl, A.T., Funari, V.A., Gokey, J.J., et al. (2016). Single-cell RNA sequencing identifies diverse roles of epithelial cells in idiopathic pulmonary fibrosis. JCI Insight *1*, e90558.

Yang, L., Han, Y., Nilsson-Payant, B.E., Gupta, V., Wang, P., Duan, X., Tang, X., Zhu, J., Zhao, Z., Jaffre, F., et al. (2020). A human pluripotent stem cell-based platform to study SARS-CoV-2 tropism and model virus infection in human cells and organoids. Cell Stem Cell *27*, 125–136.e127.

Zacharias, W.J., Frank, D.B., Zepp, J.A., Morley, M.P., Alkhaleel, F.A., Kong, J., Zhou, S., Cantu, E., and Morrisey, E.E. (2018). Regeneration of the lung alveolus by an evolutionarily conserved epithelial progenitor. Nature *555*, 251–255.

Zhao, Y., Zhao, Z., Wang, Y., Zhou, Y., Ma, Y., and Zuo, W. (2020). Single-cell RNA expression profiling of ACE2, the putative receptor of Wuhan 2019-nCov. bioRxiv. https://doi.org/10.1101/2020.01.26.919985.

Zhou, L., Niu, Z., Jiang, X., Zhang, Z., Zheng, Y., Wang, Z., Zhu, Y., Gao, L., Wang, X., and Sun, Q. (2020). Systemic analysis of tissue cells potentially vulnerable to SARS-CoV-2 infection by the protein-proofed single-cell RNA profiling of ACE2, TMPRSS2 and Furin proteases. bioRxiv.

Ziegler, C.G.K., Allon, S.J., Nyquist, S.K., Mbano, I.M., Miao, V.N., Tzouanas, C.N., Cao, Y., Yousif, A.S., Bals, J., Hauser, B.M., et al. (2020). SARS-CoV-2 receptor ACE2 is an interferon-stimulated gene in human airway epithelial cells and is detected in specific cell subsets across tissues. Cell *181*, 1016–1035.e1019.

STAR★METHODS

KEY RESOURCES TABLE

REAGENT or RESOURCE	SOURCE	IDENTIFIER
Antibodies		
Anti-human CD31 APC	Biolegend	Cat#303116; RRID:AB_187751
Anti-human CD45 APC	Biolegend	Cat#368512; RRID:AB_2566372
Anti-human EpCAM FITC	Biolegend	Cat#324204; RRID:AB_756078
Mouse anti-HTII-280 IgM	Terrace Biotech	Cat#TB-27AHT2-280; RRID:AB_2832931
PE goat anti-mouse IgM	Thermo Fisher Scientific	Cat#12-5790-81; RRID:AB_465939
Rabbit pro-SFTPC	Merck Millipore	Cat#Ab3786; RRID:AB_92588
DAPI	Sigma-Aldrich	Cat#D9543
Alexa Fluor 647 Phalloidin	Thermo Fisher Scientific	Cat#A22287; RRID:AB_2620155
CRB3 Antibody	NOVUS BIOLOGICALS	Cat#NBP1-81186; RRID:AB_11038157
Rabbit anti-SCRIB	GeneTex	Cat#GTX107692; RRID:AB_1241297
Goat RAGE/AGER antibody	R&D Systems	Cat#AF1145; RRID:AB_354628
Recombinant Anti-Aquaporin 5 antibody	abcam	Cat#ab92320; RRID:AB_2049171
Mouse anti-ABCA3	Seven Hills Bioreagents	Cat#WRAB-ABCA3; RRID:AB_577286
Rabbit anti-HOPX	Santa Cruz	Cat#sc-30216; RRID:AB_2120833
Rat anti-SCGB1A1	R&D SYSTEMS	Cat#MAB4218; RRID:AB_2183286
Rabbit anti-KRT5	Biolegend	Cat#905501; RRID:AB_2565050
Mouse anti-TP63	abcam	Cat#ab735; RRID:AB_305870
Anti-ACE2 antibody	abcam	Cat#ab15348; RRID:AB_301861
TMPRSS2 antibody (H-4)	Santa Cruz Biotechnology	Cat#515727
Anti-pro-surfactant Protein C	abcam	Cat#ab90716; RRID:AB_10674024
Anti-dsRNA IgM monoclonal antibody	SCICONS	Cat#10030005
SARS-CoV-2-Nucleocapsid antibody (NP)	Sino Biological	Cat#40143-MM05; RRID:AB_2827977
SARS-CoV Nucleoprotein antibody (NP)	Sino Biological	Cat#40143-T62
Human/Mouse E-Cadherin antibody	R&D SYSTEMS	Cat#AF748; RRID:AB_355568
MX1 antibody	GeneTex	Cat#GTX110256; RRID:AB_1950963
Alexa Fluor 488 AffiniPure Donkey Anti-Rabbit IgG (H+L)	Jackson ImmunoResearch Laboratories	Cat#711-545-152; RRID: AB_2313584
Alexa Fluor 594 AffiniPure Donkey Anti-Rabbit IgG (H+L)	Jackson ImmunoResearch Laboratories	Cat#711-585-152; RRID: AB_2340621
Alexa Fluor 647 AffiniPure Donkey Anti-Rabbit IgG (H+L)	Jackson ImmunoResearch Laboratories	Cat#711-605-152; RRID: AB_2492288
Alexa Fluor 488 AffiniPure Donkey Anti-Mouse IgG (H+L)	Jackson ImmunoResearch Laboratories	Cat#715-545-151; RRID: AB_2341099
Alexa Fluor 594 AffiniPure Donkey Anti-Mouse IgG (H+L)	Jackson ImmunoResearch Laboratories	Cat#715-585-151; RRID: AB_2340855
Alexa Fluor 647 AffiniPure Donkey Anti-Mouse IgG (H+L)	Jackson ImmunoResearch Laboratories	Cat#705-605-147; RRID: AB_2340437
Alexa Fluor 488 AffiniPure Donkey Anti-Goat IgG (H+L)	Jackson ImmunoResearch Laboratories	Cat#705-545-147; RRID: AB_2336933
Alexa Fluor 594 AffiniPure Donkey Anti-Goat IgG (H+L)	Jackson ImmunoResearch Laboratories	Cat#705-585-147; RRID: AB_2340433
Alexa Fluor 647 AffiniPure Donkey Anti-Goat IgG (H+L)	Jackson ImmunoResearch Laboratories	Cat#705-605-147; RRID: AB_2340437

(*Continued on next page*)

Continued

REAGENT or RESOURCE	SOURCE	IDENTIFIER
Goat anti-Mouse IgM (heavy chain), Alexa Fluor 555	Thermo Fisher Scientific	Cat#A-21426; RRID:AB_2535847
Donkey anti-Rabbit IgG (H+L) Alexa Fluor 488	Thermo Fisher Scientific	Cat#A-21206; RRID:AB_2535792
Donkey anti-Rabbit IgG (H+L), Alexa Fluor 555	Thermo Fisher Scientific	Cat#A-31572; RRID:AB_162543
Donkey anti-Rabbit IgG (H+L), Alexa Fluor 647	Thermo Fisher Scientific	Cat#A-31573; RRID:AB_2536183
Donkey anti-Mouse IgG (H+L), Alexa Fluor 488	Thermo Fisher Scientific	Cat#A-21202; RRID:AB_141607
Donkey anti-Mouse IgG (H+L) Alexa Fluor 647	Thermo Fisher Scientific	Cat#A-31571; RRID:AB_162542
Donkey anti-Rat IgG (H+L), Alexa Fluor 488	Thermo Fisher Scientific	Cat#A-21208; RRID:AB_2535794
Bacterial and Virus Strains		
SARS-CoV-2: BetaCov/Korea/KCDC03	Kim et al., 2020b	N/A
Biological Samples		
Human lung tissue samples	This paper	N/A
Chemicals, Peptides, and Recombinant Proteins		
Collagenase/Dispase	Sigma-Aldrich	Cat#10269638001
Dispase II	Sigma-Aldrich	Cat#4942078001
DNase I	Sigma-Aldrich	Cat#D4527-10KU
RBC lysis solution	Roche	Cat#11814389001
Accutase	STEMCELL Technologies	Cat#07920
Crystal violet solution	Sigma-Aldrich	Cat#V5265
Modified Eagle Medium	GIBCO	Cat#11935-046
HI FBS	GIBCO	Cat#10082-147
Serum-free DMEM	GIBCO	Cat#41966-029
FBS	GIBCO	Cat#16000-044
Human Serum	Sigma-Aldrich	Cat#H4522
Corning Matrigel Growth Factor Reduced (GFR) Basement Membrane Matrix, LDEV-free	CORNING	Cat#354230
Corning Matrigel Growth Factor Reduced (GFR) Basement Membrane Matrix, Phenol Red-free, LDEV-free	CORNING	Cat#356231
Y-27632	Sigma-Aldrich	Cat#Y0503
Advanced DMEM/F12	Thermo Fisher Scientific	Cat#12634010
B27 supplement	Thermo Fisher Scientific	Cat#17504044
FGF 7	PEPROTECH	Cat#100-19
FGF 10	PEPROTECH	Cat#100-26
Noggin	PEPROTECH	Cat#120-10C
EGF	PEPROTECH	Cat#100-15
N-Acetylcysteine	Sigma-Aldrich	Cat#A9165
Nicotinamide	Sigma-Aldrich	Cat#N0636
SB431542	Calbiochem	Cat#616461
CHIR99021	TOCRIS	Cat#4423
SB202190	Sigma-Aldrich	Cat#S7067
A83-01	TOCRIS	Cat#2939
Primocin	Invivogen	Cat#ant-pm-1
HEPES	GIBCO	Cat#15140-122

(*Continued on next page*)

Continued		
REAGENT or RESOURCE	SOURCE	IDENTIFIER
Penicillin / Streptomycin	GIBCO	Cat#15630-080
Glutamax-I	GIBCO	Cat#35050-061
Amphotericin B	Sigma-Aldrich	Cat#A2942
Gentamicin	Sigma-Aldrich	Cat#G1397
Lysotracker Deep Red	Invitrogen	Cat#L12492
KaryoMAX™ Colcemid™ Solution in PBS	GIBCO	Cat#15212012
EMbed812	Electron Microscopy Science	Cat#EMS14120
Glutaraldehyde	Sigma-Aldrich	Cat#G5882
4% Paraformaldehyde	Biosesang	Cat#PC2031-050-00
Antifade mounting medium	VECTASHIELD	Cat#H-1000-10
Rapiclear®	SUNJin Lab	Cat#RC152001
FSC22 Frozen section	Leica	Cat#3801480
Triton X-100	Sigma-Aldrich	Cat#T9284-100ML
Normal donkey serum	Jackson immuno	Cat#NC9624464
Sucrose	Sigma-Aldrich	Cat#S0389-500G
Bovine Serum Albumin solution	Sigma-Aldrich	Cat#AB412-100ML
TrypLE Select	GIBCO	Cat#12563-029
TRIzol	Thermo Fisher Scientific	Cat#15596026
Critical Commercial Assays		
C-chip Neubauer improved	iNCYTO	Cat#DHC-01
SuperScript IV	Thermo Fisher Scientific	Cat#18091050
Power SYBR Green PCR Master Mix	Thermo Fisher Scientific	Cat#4367659
RNeasy Plus Mini Kit	QIAGEN	Cat#74134
QIAamp Viral RNA Mini Kit	QIAGEN	Cat#52906
QIAquick PCR Purification Kit	QIAGEN	Cat#28106
pGEN-T Easy Vector Systems	Promega	Cat#A1360
RiboMAX Large Scale RNA Production Systems with T7	Promega	Cat#P1300
NucleoSpin RNA, Mini kit for RNA purification	MACHEREY-NAGEL	Cat#740955.50
Chromium Single cell 3′ GEM, Library & Gel Bead kit v3	10X Genomics	Cat#PN-1000075
Truseq Stranded Total RNA Gold kit	Illumina	Cat#20020599
CellTiter-Glo 3D Cell Viability Assay	Promega	Cat#G9681
LDH-Glo Cytotoxicity Assay	Promega	Cat#J2380
Deposited Data		
h3AC and h3BC bulk RNA sequencing data	This paper	EGA: EGAS00001004508
Vero cell bulk RNA sequencing	This paper	GEO: GSE159316
h3AC single cell RNA sequencing data	This paper	EGA: EGAS00001004508
h3AC and h3BC transmission electron microscopy images	This paper	EMPIAR-10533
Supplemental information of this study	This paper	https://doi.org/10.17632/hzbrzdkcfr.1
Experimental Models: Cell Lines		
Monkey: Vero cell (female)	Kim et al., 2020b	RRID:CVCL_0059
Human: 293T-HA-Rspo1-Fc cell	Trevigen	Cat#3710-000-01
Oligonucleotides		
See Table S7. List of quantitative PCR primers		N/A

(*Continued on next page*)

Continued

REAGENT or RESOURCE	SOURCE	IDENTIFIER
Software and Algorithms		
FACSDiva software version(ver. 6.1.3)	BD Biosciences	N/A
FlowJo software	Tree Star, Inc	N/A
R (ver. 3.6.0)	Comprehensive R Archive Network	https://cran.r-project.org
Python (ver. 2.7.16)	Python Software Foundation	https://www.python.org/
Cell Ranger (ver. 3.1.0)	10X Genomics	Ver. 3.1.0; RRID:SCR_017344
Subset-bam	10X Genomics	https://github.com/10XGenomics/subset-bam
Seurat (v3)	Stuart et al., 2019	https://satijalab.org/seurat; RRID:SCR_016341
SC3	Kiselev et al., 2019	https://github.com/hemberg-lab/SC3; RRID:SCR_015953
Enhanced Volcano	Blighe et al., 2018	https://github.com/kevinblighe/EnhancedVolcano
ComplexHeatmap	Gu et al., 2016	https://github.com/jokergoo/ComplexHeatmap
Samtools (ver. 1.9)	Kiselev et al., 2019; Li et al., 2009	http://www.htslib.org; RRID:SCR_002105
Varscan2 (ver. 2.4.2)	Koboldt et al., 2012	http://dkoboldt.github.io/varscan/; RRID:SCR_006849
Strelka2 (ver. 2.9.2)	Saunders et al., 2012	https://sites.google.com/site/strelkasomaticvariantcaller/home; RRID:SCR_005109
Integrated Genomics Viewer	Robinson et al., 2011	http://software.broadinstitute.org/software/igv/
STAR (ver. 2.6.1)	Dobin et al., 2013	https://github.com/alexdobin/STAR; RRID:SCR_015899
RSEM (ver. 1.3.1)	Li and Dewey, 2011	https://github.com/deweylab/RSEM; RRID:SCR_013027
DESeq2	Love et al., 2014	http://bioconductor.org/packages/devel/bioc/vignettes/DESeq2/inst/doc/DESeq2.html; RRID:SCR_015687
Enrichr	Kuleshov et al., 2016	https://maayanlab.cloud/Enrichr/
ZEN	ZEISS	Ver. 2.3
Tecnai Microscope control software	FEI	Ver. 4.17 SP1
SoftMax Pro software	Molecular Devices	N/A
Cytovision	Leica	Ver 7.4
Other		
Nunc Lab-Tek II Chamber Slide System	Thermo Fisher Scientific	Cat#154534
SPL Collagen Type I Coated Ware	SPL	Cat#30208
Monkey reference genome, ChlSab1.1	Ensembl release 100	ftp://ftp.ensembl.org/pub/release-100/fasta/chlorocebus_sabaeus/dna/Chlorocebus_sabaeus.ChlSab1.1.dna.toplevel.fa.gz
SARS-CoV-2 reference sequence, NC_045512.2	NCBI Reference Sequence	https://www.ncbi.nlm.nih.gov/nuccore/NC_045512.2?report=fasta
Human reference genome, GRCh38.p13	Ensembl release 100	ftp://ftp.ensembl.org/pub/release-100/fasta/homo_sapiens/dna/Homo_sapiens.GRCh38.dna.primary_assembly.fa.gz
SARS-CoV-2 uninfected and infected human lung bulk RNA sequencing data	Blanco-Melo et al., 2020	GEO: GSE147507
SARS-CoV-2 uninfected and infected 2D cell line bulk RNA sequencing data	Emanuel et al., 2020a	GEO: GSE148729

RESOURCE AVAILABILITY

Lead Contact

Further information and requests for resources and reagents should be directed to and will be fulfilled by the Lead Contact, Young Seok Ju (ysju@kaist.ac.kr).

Materials Availability

All 3D models generated in this study are available from the Lead Contact with a completed Materials Transfer Agreement.

Data and Code Availability

Processed single cell RNA sequencing data including Seurat objects, cellular metadata, and counts/UMI tables are available on Synapse (accession syn22146555). Scripts reproducing the single cell RNA sequencing analysis are deposited on GitHub (https://github.com/ju-lab/SARS-CoV-2_alveolar_organoids). Bulk RNA and single cell RNA sequencing datasets are uploaded on the European Genome-Phenome Archive (EGA) with accession ID EGAS00001004508 for human-derived data and the NCBI Gene Expression Omnibus (GEO) GSE159316 for Vero cell data. Transmission electron microscopy images (n > 300) are uploaded in EM Public Image Archive (EMPIAR) with an accession ID EMPIAR-10533.

EXPERIMENT MODEL AND SUBJECT DETAILS

Human Tissues

For the establishment of human lung 3D-culture models, human distal lung parenchymal tissues from deidentified lungs not required for transplantation were obtained from adult donors with no background lung pathologies from Papworth Hospital Research Tissue Bank (T02233), and Addenbrookes Hospital (Cambridge University NHS foundations trust) under the collaboration of Cambridge Biorepository for Translational Medicine (CBTM) project. Appropriate Human Tissue Act (HTA) guidance was followed; For the viral infection and following analysis, human lung tissues were obtained from patients undergoing lobectomy surgery at Seoul National University Hospital (SNUH) with written informed consent from approval of the ethical committee (approval no. C-1809-137-975). hAT2 3D cultures for infection studies were established from normal counterpart tissues in lung cancer patients or an idiopathic pulmonary fibrosis (IPF) patient. Overall, h3ACs at passage 0-3 from a total of 11 donors and h3BCs at passage 2 from 3 donors were used in our infection experiments (Table S6).

Virus particle preparation for infection

SARS-CoV-2 virus strain is BetaCov/Korea/KCDC03/2020 (Kim et al., 2020b). The patient (KCDC03) was diagnosed with COVID-19 on January 26, 2020, after traveling to Wuhan, China. The virus was also sequenced in the previous paper (Kim et al., 2020a). For virus replication, Vero cells were infected with MOI of 0.01 and grown under DMEM with 2% FBS, 1% P/S for 48 hours at 37°C 5% CO_2 (hereafter, the media is termed as infection media). Media was centrifuged with 2500rpm for 25min, and supernatant without cell debris was stocked at -80°C with 4×10^6 pfu / ml.

METHOD DETAILS

Human lung tissue dissociation and flow cytometry

Distal lung parenchymal tissue was processed as soon as possible in order to minimize cell yield loss and maintain cell viability. Briefly, fresh tissue was washed in cold PBS and minced into small (1 mm) pieces with a scalpel, followed by further dissociation using pre-warmed digestion buffer containing 2U/ mL Dispase II (Sigma-Aldrich, CORNING), 1 mg/mL Dispase/Collagenase (Sigma-Aldrich) and 0.1 mg/mL DNase I (Sigma-Aldrich) in PBS at 37°C for 1 hr with agitation. Tissue cell suspensions were filtered through a 100 μm cell strainer into a 50 mL falcon tube to remove cell debris, and washed with 10 mL of DMEM (GIBCO, Thermo Fisher Scientific). Cells were centrifuged at 350 g for 10 min, supernatant carefully aspirated, and cell pellet resuspended in 5 mL red blood cell lysis buffer for 5 min at room temperature (RT). The reaction was quenched using 5 mL of DMEM, and the entire 10 mL of cell suspension was transferred to a 15 mL falcon tube, followed by 10 min centrifugation at 350 g. Supernatant was removed, and the cell pellet was resuspended in 10% FBS in PBS (PF10 buffer) for counting. Cells were prepared for flow cytometry with primary antibodies CD31-APC (Biolegend, 303116), CD45-APC (Biolegend, 368512), EpCAM-FITC (Biolegend, 324204) and HTII-280-IgM (Terrace Biotech, TB-27AHT2-280) at 1:40 per 4 million cells for 30 min on ice. Following two washes with cold PF10 buffer and centrifugation at 350 g for 5 min, cells were stained with secondary PE goat anti-mouse IgM (eBioscience, 12-5790-81) for HTII-280 at 1:100. Stained cells were washed with PF10 buffer, and counted using a hemocytometer to assess dilution required for final volume. Cells were diluted at a concentration of 30 million cells/ mL and filtered through a 35 μm cell strainer into polypropylene FACS tubes. Cell sorting was performed on an Aria III fusion (UK; BD Biosciences) or an Aria II (South Korea; BD Biosciences) using a 100 μm nozzle (UK) or 85 μm nozzle, and data were analyzed with FlowJo software (UK; Tree Star, Inc.) or FACS DIVA (South Korea, BD Biosciences).

In vitro 3D culture and passage

Freshly isolated HTII-280$^+$ and HTII-280$^-$ cells derived from CD31$^-$CD45$^-$EpCAM$^+$ cells of human distal lungs were resuspended in base medium (Advanced DMEM/F12 (Thermo Fisher Scientific) supplemented with 10 mM HEPES (GIBCO), 1 U/mL Penicillin/Streptomycin (GIBCO), 1 mM *N*-Acetylcysteine (Sigma-Aldrich), and 10 mM Nicotinamide (Sigma-Aldrich)). Growth factor-reduced (GFR) Matrigel (CORNING) was added to the cell suspension at a ratio of 1:1, and 100 μL of suspension was added to a 24-well transwell insert with a 0.4 μm pore (CORNING) so that there were approximately 10 × 10^4 cells per insert. GFR-Matrigel was allowed to solidify for 1 hr at 37°C, after which 500 μL of pre-warmed alveolar media (base medium supplemented with 1 x B27 (Thermo Fisher Scientific), 10% R-SPONDIN-1 (Cambridge Stem Cell Institute tissue culture core facility, manually produced), 50 ng/ml human EGF (PEPROTECH), 100 ng/ml human FGF7/KGF (PEPROTECH),100 ng/ml human FGF10 (PEPROTECH), 100 ng/ml NOGGIN (PEPROTECH),10 μM SB431542 (TOCRIS) and 3 μM CHIR99021 (TOCRIS)) was added to each lower chamber. For assessment of the effect of Wnt activity on hAT2 culture ability, primary cultures were also established without CHIR99021. Cultures were maintained under standard cell culture conditions (37°C, 5% CO_2), with media changes every 2-3 days. Y-27632 (10 μM, Sigma-Aldrich) was added for the first 48 hr of culture to promote cell survival. To avoid the growth of fungal and bacterial infection, 250 ng/mL Amphotericin B and 50 μg/mL gentamicin were added to culture medium for 5 days. For culture in 48-well plates, 5 × 10^3 cells were resuspended in 100% GFR-Matrigel, and allowed to solidify in a 20 μL droplet per well at 37°C for 20 min, followed by submersion in 250 μL of pre-warmed medium. Cellular structures with a surface area of more than 500 μm at day 14 of culture were counted at day 14 to assess colony forming efficiency, and were either fixed and stained at day 21 for analysis, or enzymatically dissociated into single cells for further culture without sorting. Cells in 3D cultures were passaged at different days depending on size, with culture days varying from 21-35 days. For passaging, Matrigel was disrupted by incubation with dispase (Sigma-Aldrich) at 37°C for 45 min, followed by single cell-dissociation through addition of TrypLE (GIBCO) for 5 min at 37°C. The reaction was quenched with base medium, and cells were centrifuged at 350 x g for 5 min. Cells were resuspended in fresh GFR-Matrigel at a ratio of 5 × 10^3 (48-well plates) or 10 × 10^4 (24-well transwell inserts) cells as before. Bronchial cells were passaged every 21-28 days due to accelerated growth compared with hAT2 cells, and were cultured in previously reported medium conditions (Sachs et al., 2019) with the following concentration/factor edits; 100 ng/ml human FGF10, 10% R-SPONDIN-1, 10 μM SB431542 (instead of A83-01).

Karyotype of *in vitro* 6 months grown h3ACs

Cultured h3ACs for 6 months were retrieved from Matrigel using Cell Recovery Solution (Corning). h3ACs were dissociated into single cells using Accustase (STEMCELL Technologies) at 37°C for 5-10 minutes. After washing with PBS once, h3ACs were incubated with colcemide (GIBCO) at 37°C for 45 minutes. After washing with PBS once, h3AC cells were incubated with KCl to enlarge the cells. After fixation with acetone, cells were stained with Giemsa. Karyotypes were observed using Cytovision (Leica).

Assessment of lung lineage transcripts by qRT-PCR

Freshly sorted HTII-280$^+$ and HTII-280$^-$ cells were lysed with TRIzol, and RNA was extracted. RNA was reverse transcribed using SuperScript IV (Thermo Fisher Scientific), and were assessed using the following TaqMan probes; SFTPC (Hs00951326_g1), TP63 (Hs01114115_m1), SCGB1A1 (Hs00171092_m1) (Table S7).

Differentiation of hAT2 cells into hAT1 cells in 2D culture

h3ACs were dissociated into single cells with Accutase (STEMCELL Technologies) at 37°C for 5-10 minutes. Cells were washed with PBS and then seeded in Lab-tek 8 well slide glass (Thermo Fisher Scientific) with 25,000 cells per well or seeded in Collagen I coated 8 well slide glass (SPL). The cells were grown under 10% human serum (Sigma), 1% P/S in DMEM. After 4 days incubation, cells were allowed to attach to slides and differentiate.

SARS-CoV-2 infection of 3D culture cells and Vero cells

For 3D culture cells, cells were recovered from Matrigel with Cell Recovery Solution (CORNING). 3D culture cells were sheared with 1000 p pipette tips and incubated with Accutase (STEMCELL Technologies) at 37°C for 5-10 minutes. After washing, the number of 3D culture cells was calculated by hemocytometer C-chip (iNCYTO), and the appropriate amount of SARS-CoV-2 virus particles was prepared from a stock vial. 3D culture cells were resuspended with each media and were infected with virus multiplicity of infection (MOI) of 1.0 or 0.1 for 2 hr at 37°C 5% CO_2.

After 2 hr incubation, 3D culture cells were washed twice with Advanced DMEM/F12 with 1 U/ml Penicillin/Streptomycin, 10 mM HEPES, and 1% Glutamax (v/v) (hereafter referred to as ADF+++) and embedded in 50 μl of GFR-Matrigel (CORNING) in 24-well plate. Each well contained at least 10,000 cells. To release viral particles from the infected cells, Matrigel was physically disrupted, and cells were lysed by freezing at −80°C and thawing 3 times. Live virus titers were determined by plaque assay and viral RNA titer was calculated using qRT-PCR.

For Vero cell infection, Vero cells were prepared in DMEM with 10% FBS and 1% Penicillin/Streptomycin (GIBCO). At the day of SARS-CoV-2 infection, media was aspirated from 6-well plates, and SARS-CoV-2 viral particles with MOI of 0.01 in 500ul of DMEM with 2% FBS and 1% Penicillin/Streptomycin (GIBCO) were added to Vero cells on the 6-well plates for 1 hr at 37°C 5% CO_2. After

1 hr, infection media was aspirated, and cells were washed with cold PBS twice. Then, 2 mL of DMEM with 2% FBS and 1% p/s solution was added to each 6-well. All work was performed in a Class II Biosafety Cabinet under BSL-3 conditions at Korea Center for Disease Control (KCDC).

Viral RNA extraction

For viral RNA extraction in the infected cells, cells were retrieved from Matrigel using 500 ul of Cell Recovery Solution (Corning), and were lysed by repetitive freezing at −80°C and thawing 3 times. Then, QIAamp Viral RNA Mini Kit (Quiagen) was used to obtain SARS-CoV-2 viral RNA according to the manufacturer's instructions. For collection of viral RNAs from culture media, 140 ul of the culture media was obtained followed by QIAamp Viral RNA Mini Kit (QIAGEN).

Cellular total RNA extraction

For h3ACs and h3BCs, cells were retrieved from Matrigel using Cell Recovery Solution (45 minutes incubation at 4°C; Corning). For Vero cells, cells were detached using TrypLE Select (5 minutes incubation at 37°C; GIBCO). Cell pellets were obtained after centrifugation of retrieved cells at 300 g for 3-5 minutes at 4°C. Total RNA was extracted using RNeasy Plus Mini Kit (QIAGEN).

Viral RNA copy number calculation with qRT-PCR

Purified viral RNAs were reverse-transcribed using SuperScript IV (Thermo Fisher Scientific). Viral *N* gene with 2019-nCoV_N3-F and 2019-nCoV_N3-R probes (CDC) was targeted for qRT-PCR (Table S7). Each RNA sample was measured three times. Viral RNA copy number was estimated using the standard curve of virus copy number estimated by Ct value of the qRT-PCR results.

The viral copy number standard curve was generated as described below. Positive viral RNA template was reverse-transcribed, and cDNA was amplified with the CDC designed N gene primers and cloned into pGEM-T Easy vector (Promega, USA). The resultant plasmid DNA was linearized with PstI restriction enzyme and purified with a QIAquick PCR Purification Kit (QIAGEN). Purified template was *in vitro* transcribed by RiboMAX Large Scale RNA Production System with T7 RNA polymerase (Promega). RNA transcript was further purified with the NuceloSpin RNA Mini kit (MACHEREY-NAGEL) and quantified with spectrophotometer at 260 nm. The quantified RNA was serially diluted and each diluted sample was reverse transcribed to measure Ct value. Then, the standard curve for viral copy number with Ct values was depicted using the Ct values with corresponding RNA doses.

Validation of upregulated genes with qRT-PCR

To validate upregulated interferon genes (*INFB1*, *INFL1*, and *INFL2/3*) and ISGs (*IFI44L*, *IFI6*, *IFIT1*, and *MX1*), infected h3ACs were harvested at 0 and 3 dpi. Total cellular RNA was extracted as described above. From equal quantity of RNA (50 ng), cDNA was generated using SuperScript III Reverse Transcriptase kit (Invitrogen) as described previously (Pfaffl, 2001) and stored at −20°C until use. Real time PCR assay (qRT-PCR) was conducted by the ABI applied biosystems using Power SYBR Green PCR Master Mix (Applied Biosystems) (Table S7). The relative amounts of cytokine mRNA present (normalized with GAPDH) was determined by $2^{-\Delta\Delta Ct}$ method.

Live virus titer calculation with plaque assay

To prepare viral specimens released from infected h3AC and h3BC cells, Matrigel was disrupted by repetitive pipetting and cells were lysed by −80°C freezing and thawing once. In the case of Vero cells, culture media were directly used as viral specimens to be used in plaque assays. To perform plaque assays, newly seeded Vero cells in 12-well plates were prepared. Test specimens derived from h3ACs, h3BCs, and Vero cells were serially diluted by a scale of $10^0 - 10^5$. Then, 250ul of each diluted specimen was dispensed into the designated well, and incubated for 1hr at 37°C, 5% CO_2. After 1 hr, the diluted specimens were aspirated. Vero cells were washed with PBS two times, then agar and Modified Eagle's Medium (Thermo Fisher Scientific) were poured into each well. After agar mixture was solidified, the mixtures were fixed with 4% PFA for 3 days, and stained with crystal violet (Sigma-Aldrich). From the number of colonies, live virus titers were calculated.

Viability and cytotoxicity test

To measure the viability and cytotoxicity in h3ACs and Vero cells after SARS-CoV-2 infection with MOI of 1.0 and 0.01, respectively, infected h3ACs were embedded in Matrigel with 10,000 cells per a well. CellTiter-Glo 3D Cell Viability Assay (Promega) was used to test viability according to the manufacturer's instructions. Briefly, 250 ul of CellTiter-Glo 3D reagent was added to each well in 24-well plates (200ul media with 50ul Matrigel, and Matrigel was disrupted. The mixture of the reagent and media was stirred up, then dispensed into luminescence plates. After 25 mins of incubation, we recorded luminescent intensity using Spectramax L (Molecular Devices). Three biological replicates were performed for each time point.

To measure lactate dehydrogenase levels released from ruptured cells, LDH Glo Cytotoxicity Assay (Promega) was used according to the manufacturer's instructions. Briefly, Triton X-100 (Sigma-Aldrich) was added to 100ul of culture media (0.2% Triton X-100) to inactivate live SARS-CoV-2 virus. Then, storage buffer (Tris-HCl pH 200mM pH7.3, 10% glycerol, and 1% BSA) was added to dilute the media as indicated by the manufacturer's protocol. Diluted media was incubated with the same amount of LDH Detection Reagent (Promega) for 1 hr. After 1 hr incubation, the intensity of luminescence was measured by Spectramax L (Molecular Devices).

Immunofluorescence staining of paraffin-embedded h3ACs and Lysotracker

Control h3ACs were fixed and embedded in a paraffin block (Lee et al., 2014). Pre-cut 7 μM paraffin sections were de-waxed and re-hydrated (sequential immersion in xylene, 100% EtOH, 90% EtOH, 75% EtOH, distilled water) and either stained with hematoxylin and eosin (H&E) or immunostained. For antigen retrieval, slides were submerged into pre-heated citrate antigen retrieval buffer (10 mM sodium citrate, pH 6.0) and allowed to boil for 15 min. Slides were cooled in a buffer for 20 min, washed in running water for 3 min, and permeabilized with 0.3% Triton-X in PBS for 15 min. Following permeabilization, sections of 3D models were blocked for 1 hr in 5% normal donkey serum in PBS at RT, and incubated with primary antibody mixtures overnight at 4°C at the following dilutions; rabbit pro-SFTPC (1:500, Merck Millipore, Ab3786), mouse anti-HTII-280 (1:500, Terrace Biotech, TB-27AHT2-280), rat anti-SCGB1A1 (1:200, R&D systems, MAB4218), rabbit anti-KRT5 (1:500, Biolegend, 905501), Rabbit anti-ABCA3 (1:300, Seven Hills Bioreagents, WRAB-ABCA3), mouse anti-TP63 (1:500, abcam, ab735), and rabbit anti-HOPX (1:200, Santa Cruz Biotechnologies, sc-30216), and rabbit anti-SCRIB (1:100, GeneTex, GTX107692). Antibodies were removed with three PBS washes, and samples were incubated with Alexa Fluor-couple secondary antibodies (1:1000, Jackson ImmunoResearch Laboratories) for 1 hr at RT. Following PBS washes, nuclei were stained with DAPI for 5 min, slides mounted with Rapiclear (SUNjin lab), and sealed with clear nail polish. For Lysotracker staining of lysosomes, live h3ACs in 48-well plates were incubated *in situ* with 50 ng/μL of Lysotracker (Invitrogen, L12492), diluted in pre-warmed expansion medium, for 30 min at 37°C. Lysotracker was removed, and Matrigel suspension was carefully washed for 5 min in PBS, followed by addition of fresh, pre-warmed expansion medium. Cells were protected from light and either imaged immediately using an EVOS cell imaging system, or analyzed by FACS on an Aria III fusion (BD Biosciences) and FlowJo software (Tree Star, Inc.)

Immunofluorescence staining of infected h3ACs with cryo-section

Both uninfected and infected h3ACs were fixed in 4% paraformaldehyde (PFA) for 3 hr at 4°C, and then dehydrated in PBS with 30% sucrose (v/v) (Sigma-Aldrich). The h3ACs were embedded with optimal cutting temperature (OCT) compound (Leica) and cut into 7-10 μm thick sections. The sections were blocked with PBS with 5% normal donkey serum and 1% Triton X-100 (Sigma-Aldrich). The sections were incubated with primary antibodies overnight at 4°C, then washed, and incubated with host matched Alexa Fluor-couple secondary antibodies (1:1000, Jackson ImmunoResearch Laboratories) for 1.5 hr at RT. Following DAPI incubation, slides were mounted. E-cadherin (1:300, R&D SYSTEMS, AF748), NP (1:200, Sino Biological, 40143-MM05), NP (1:200, Sino Biological, 40143-T62), dsRNA (1:2, SCICONS, 10030005), ACE2 (1:400, abcam, ab15348), TMPRSS2 (1:400, Santa Cruz, 515727), pro-SFTPC (1:400, abcam, ab90716), MX1 (1:400, GeneTex, GTX110256), and HTII-280 (1:50, Terrace Biotech, TB-27AHT2) were used.

Immunofluorescence staining of whole-mount h3ACs

To check polarity of h3ACs, we conducted Z stack whole mount imunofluorescnce. H3ACs were seeded with the Phenol-free Matrigel (Corning) in the 8-well chamber slides (Thremo Fisher Scientific). Each well was fixed with 4% PFA (Biosesang) at 4°C for 45 min and washed with PBS for 5 min 2 times. Organoids were blocked with PBS with 1% BSA (Sigma-Aldrich) and 0.1% Triton X-100 (Sigma-Aldrich). The sections were incubated with primary antibodies overnight at 4°C, then washed, and incubated with host matched Alexa Fluor-couple secondary antibodies (1:1000, Jackson ImmunoResearch Laboratories) for 1.5 hr at RT. Alexa 647 Phalloidin (1:100) were incubated for 1hr at RT. Following DAPI incubation, slides were mounted. Crb3 (1:400, NOVUS BIOLOGICALS, NBP1-81185), HTII-280 (1:200, Terrace Biotech, TB-27AHT2) were used.

Immunofluorescence staining of hAT1-like cells

Attached hAT1-like cells were fixed with 4% PFA at 4°C for 3 hr. After cells were blocked in PBS with 5% normal donkey serum and 1% Triton X-100 (Sigma-Aldrich), sections were incubated with primary antibodies overnight at 4°C, then washed, and incubated with host matched Alexa Fluor-couple secondary antibodies (1:1000, Jackson ImmunoResearch Laboratories) (1:1000) for 1.5 hr at RT. Following DAPI incubation, slides were mounted. AGER (1:400, R&D SYSTEMS, AF1145), Aquaporin5 (1:200, abcam, ab92320) pro-SFTPC (1:400, abcam, ab90716) were used.

Transmission electron microscopy

Uninfected h3ACs and infected h3ACs at 2 dpi were fixed with 2.5% glutaraldehyde in 0.1 M PBS for overnight incubation at 4°C (Fujii et al., 2018). The h3ACs were washed with PBS and post-fixed with 2% osmium tetroxide for 1.5 hr. The fixed samples were dehydrated in graded ethanol, substituted with propylene oxide, and finally embedded in EMbed-812 resin (EMS). Polymerization was performed at 60°C for 24 hr. Ultrathin (100 nm) sections were prepared using an ultramicrotome (Leica, EM UC7). Images were captured with a transmission electron microscope (FEI Tecnai G2 spirit TWIN, eagle 4K CCD camera) at 120kV acceleration voltage. All work was carried out in EM & Histology Core Facility, at BioMedical Research Center, KAIST. For control EM image, data were stitched manually (Figure S4A).

Bulk RNA sequencing and data processing

Total RNA sequencing library was constructed using Truseq Stranded Total RNA Gold kit (Illumina) according to the manufacturer's protocol followed by sequencing with 2 × 100 bp using Hiseq 2500. Fastq files were aligned to GRCh38 (human cells) with SARS-CoV-2 virus sequence (NC 045512.2 from NCBI) or ChlSab1.1 (Vero cells) with SARS-CoV-2 virus sequence, yeast *ENO2* cDNA sequence (SGD: YHR174W), and human ribosomal DNA complete repeat unit sequence (U13369.1)(Kim et al., 2020a) using

STAR v2.6.1d (Dobin et al., 2013) and normalized counts of total RNA expression were calculated using RSEM v1.3.1 (Li and Dewey, 2011).

The top 100 genes with highest variations (standard deviations / mean of TPM values of each gene) in h3ACs were demonstrated as heatmap (Figures 5A and S5E) using R package "ComplexHeatmap."Enriched pathways were acquired from Enrichr website with input of the top 100 variable genes (Kuleshov et al., 2016).

Previously published bulk RNA sequencing data (fastq files) were obtained from the NCBI Gene Expression Omnibus (GEO) server under the accession number GSE147507 for COVID-19 patients and healthy donors and GSE148729 for 2D cell lines including mock and SARS-CoV-2 treatment of Calu-3, Caco-2, and NCI-H1299 at 24 hpi (Blanco-Melo et al., 2020; Emanuel et al., 2020a). The fastq files were processed as mentioned above.

Fold changes and adjusted *p-value*s of DEGs were calculated using R package "DEseq2" (Love et al., 2014). The results were demonstrated as volcano plots using R package "EnhancedVolcano" (Blighe et al., 2018). Differentially upregulated genes were selected when fold change and adjusted *p-value* of each DEG were more than 1.5 and less than 10^{-6}, respectively, in SARS-CoV-2 infected samples compared to uninfected samples (Figures S5D and S5H; Table S2). To investigate single-base substitutions and small indels, we used union sets of variants called by Strelka2 (Saunders et al., 2012) and Varscan2 (Koboldt et al., 2012) Then, in-house script was used to filter false-positive calls, and we manually curated the variant positions using Integrative Genomics Viewer (Robinson et al., 2011).

Single-cell RNA sequencing and data processing

Fastq files were aligned and UMI counts were calculated using Cell Ranger software (3.1.0) provided by the manufacturer (10X Genomics). The 10X gene count data includes 19,941 human genes and SARS-CoV-2 genome as an extra gene.

Normalization was done using a log-normalization method (same as 'NormalizeData from 'Seurat' R package) controlling the total human gene UMI count (after scaling) at 10k. After scaling, each count is log2-transformed after added by 1.

From the initial 30,027 cells with reported UMI counts across five experimental conditions, we filtered out cells with > 25% mitochondria RNA percent (n = 2,479), pathologic cell clusters characterized with KRT5-/KRT17+ (n = 13,175) (Adams et al., 2020; Habermann et al., 2020), and contaminated cells (n = 199). The remaining 14,174 cells constitutes the core dataset, and used for graphical visualization as well as clustering analyses.

Using 'Seurat V3′ package (Stuart et al., 2019), 3,000 variable features were selected, principal components (PCs) were constructed, and UMAP dimension reduction was performed based on 30 PCs. Then, unsupervised clustering analysis was carried out by constructing a shared nearest neighbor (SNN) graph (using 'FindNeighbors' function with parameters of 'dims = 1:20') and identifying clusters of cells (using 'FindClusters' function with 'resolution' parameter of 0.1). Using 'SC3′ package (Kiselev et al., 2019), additional clustering exercise was carried out and marker genes were highlighted. Specifically, 'SC3′ clusters were constructed at 'k = 9' parameter setting. Then, marker genes most representing each cluster were found and ranked by internal 'auroc' metric. The marker genes with the associated auroc > 0.85 (default) were visualized. Using 150 variable genes calculated by 'SC3′ package, enriched pathways in SARS-CoV-2 infected cells were obtained from Enrichr website (Kuleshov et al., 2016).

For investigating C > U at the 23,707 position of SARS-CoV-2 transcripts in each cell, subset-bam tool (10X Genomics) was used to get an individual bam file per each cell. Samtools (Li et al., 2009) was used to call the substitution. In house python and R scripts were used for more downstream analyses.

Statistical inference on the effective number of viral entry

To assess whether hAT2 cells tend to be infected by a single viral particle or multiple particles, we employed a likelihood approach. As a proof-of-concept, we assumed only two scenarios exist, one supporting a single viral entry and the other supporting double viral entry, then aimed to estimate the proportion of cells with a single viral particle (w). Out data consists of the observed reference (n^{r}_{cell}) and alternative (n^{v}_{cell}) read counts for each of the 547 cells reporting at least one read at the mutation site of NC_045512.2:23,707. Assuming a sequencing error rate (ε) of 0.1%, which will cover any Illumina sequencing errors or misalignment, the likelihood of data given the weight supporting a single virus scenario (w) was computed as follows.

$$L(w) = \prod_{cell=1}^{547} L_{cell}(w)$$

$$L_{cell}(w) = w \times P(Data_{cell}|\text{single entry}) + (1-w) \times P(Data_{cell}|\text{double entry})$$

$$P(Data_{cell}|\text{single entry}) = P(G=R) \times P(n^{r}_{cell}, n^{v}_{cell}|vaf_{cell} = \varepsilon)$$

$$+ P(G=V) \times P(n^{r}_{cell}, n^{v}_{cell}|\, vaf_{cell} = 1-\varepsilon)$$

$$P(Data_{cell}|\text{double entry}) = P(G = RR) \times P\left(n^r_{cell},\ n^v_{cell}|vaf_{cell} = \varepsilon\right)$$

$$+\ P(G = RV) \times P\left(n^r_{cell},\ n^v_{cell}|\ vaf_{cell} = 0.5\right)$$

$$+\ P(G = VV) \times P\left(n^r_{cell},\ n^v_{cell}|vaf_{cell} = 1 - \varepsilon\right)$$

G stands for the viral genotype for each cell, and R and V denote for the reference (wild-type) and variant (mutant) alleles in the genotype, respectively. vaf_{cell} refers to the true mutant allele frequency within an infected cell given the genotype after accounting for the sequencing error rate.

For both single- and double-entry scenarios, the prior for each genotype was computed using the mutant allele frequency estimated from the whole cell population ($vaf_{pop.}$; 4.3% = 42 variant reads out of 986 reads). For example, the prior of having the genotype with two mutant alleles in a double-entry scenario is computed as follows:

$$P\ (G = VV) =\ vaf^2_{pop}$$

Within a cell, the probability of observing the number of reference and variant reads given the mutant allele frequency, $P(n^r_{cell},\ n^v_{cell}\ |vaf_{cell})$, was computed using a binomial distribution.

QUANTIFICATION AND STATISTICAL ANALYSIS

To quantify folded and cystic 3D structures of h3ACs, we at least counted 50 organoids for each donor, and the total number of donors is 3. For quantification of colony forming efficiency (CFE) for h3AC, CFE was calculated from total 3 donors at each passage (Data is represented as mean ± SEM). In Plaque assay for live virus titer, the number of viruses was counted from two technical replicates for each designated time points (Data is represented as mean ± SEM). In qPCR for virus RNA titer and interferon induced gene validation, 3 technical replicates were used (Data is represented as mean ± SEM). To compare AT2 marker gene's expression, qPCR was conducted with 2 technical replicates (Data is represented as mean ± SEM). Viability and LDH cytotoxicity assay were replicated with 3 biological replicates (Data is represented as mean ± SEM). All the immunofluorescence images were taken at least 5 different biological replicates and representative image was used. To compare MX1 intensity in control (n = 11) and infected (n = 13) h3ACs, Wilcoxon rank sum test was used. For transmission electron microscopy, more than 10 biological replicates were taken. For karyotyping, 6 normal cell karyotypes were observed and representative image was used. RNA sequencing statistical analysis was written in "RNA sequencing and data processing" session in METHOD DETAILS. Single cell RNA sequencing statistical analysis was written in "Single cell RNA sequencing and data processing" session in Method Details.

Cell Stem Cell, Volume *27*

Supplemental Information

Three-Dimensional Human Alveolar Stem Cell Culture Models Reveal Infection Response to SARS-CoV-2

Jeonghwan Youk, Taewoo Kim, Kelly V. Evans, Young-Il Jeong, Yongsuk Hur, Seon Pyo Hong, Je Hyoung Kim, Kijong Yi, Su Yeon Kim, Kwon Joong Na, Thomas Bleazard, Ho Min Kim, Mick Fellows, Krishnaa T. Mahbubani, Kourosh Saeb-Parsy, Seon Young Kim, Young Tae Kim, Gou Young Koh, Byeong-Sun Choi, Young Seok Ju, and Joo-Hyeon Lee

Figure S1, related to Figure 1.

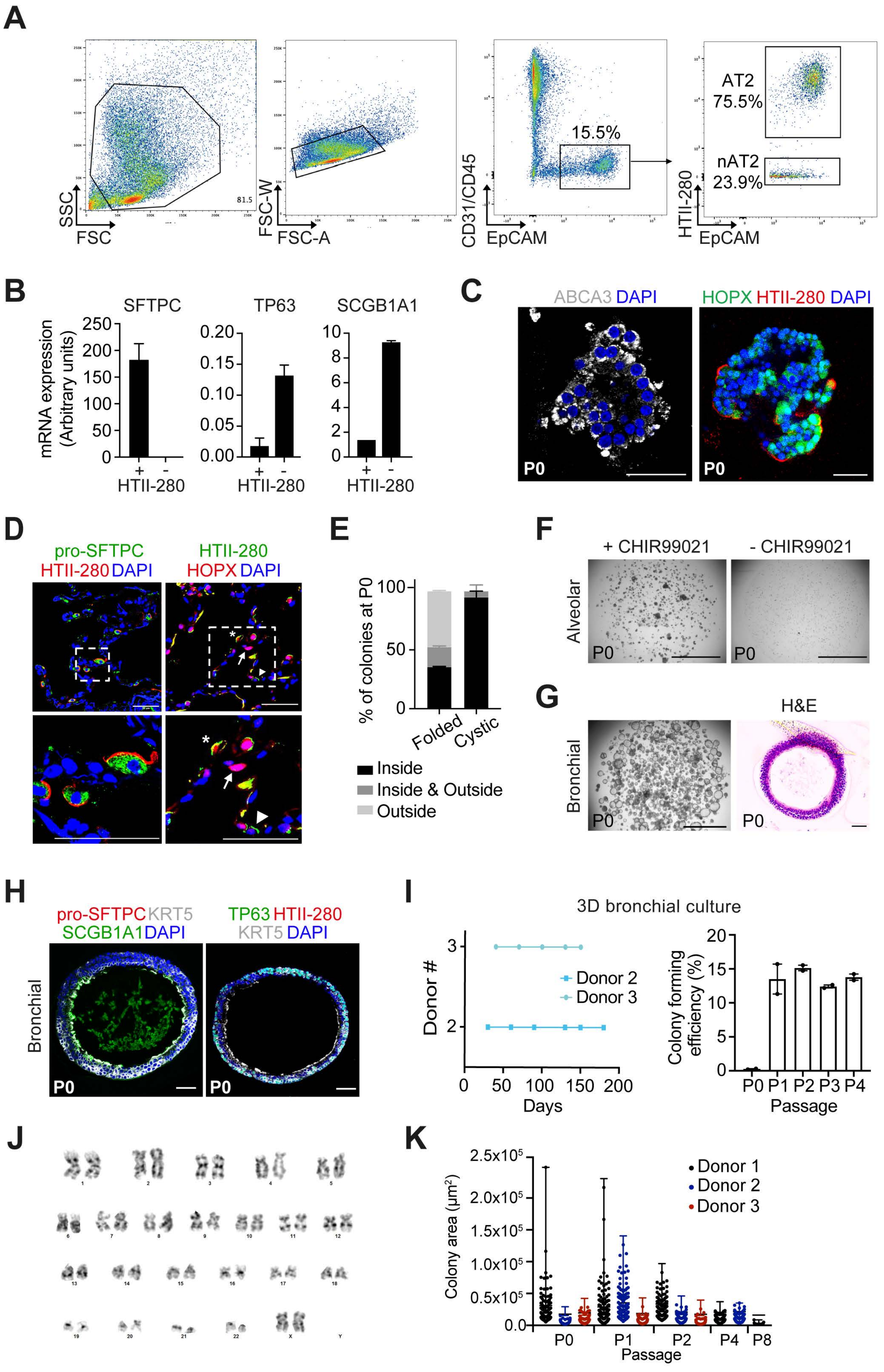

Figure S1. Establishment and characterization of three-dimensional culture of human airway and alveolar type 2 cells. Related to Figure 1.

(A) Representative flow cytometry analysis plot for isolation of hAT2 cells (CD31-CD45-EPCAM+HTII-280+) and non-hAT2 cells (nAT2, CD31-CD45-EPCAM+HTII-280-) from human distal lung parenchymal tissues.

(B) Quantitative PCR (qPCR) analysis of isolated primary human lung epithelial cells. HTII-280+ cells express much higher levels of the AT2 cell marker SFTPC, while HTII-280- cells express virtually no SFTPC and instead express higher levels of the airway markers TP63 (basal) and SCGB1A1 (secretory). Data is the mean ± SEM of arbitrary mRNA expression (n=2, technical replicates).

(C) Representative immunofluorescence (IF) images of primary hAT2 cultures expressing alveolar lineage markers. Left; ABCA3 (for hAT2, white). Right; HOPX (for hAT1-like, green), HTII-280 (for hAT2, red), and DAPI (blue). Scale bar, 50 μm.

(D) IF staining of human distal parenchymal regions of healthy human donor lungs for pro-SFTPC (green, left) and HTII-280 (red, left; green, right), and HOPX (red, right). DAPI (blue). Images are representative of individual donors (n = 3). Scale bar, 50 μm.

(E) Quantification of the folded and cystic 3D structures expressing HTII-280 in inside or/and outside of hAT2 cells in h3ACs (P0). Data is presented as mean ± SEM for 2 individual donor samples (n = 67 for donor 1, n = 50 for donor 2). n=total number of colonies scored.

(F) Representative brightfield images of primary hAT2-derived 3D alveolar cultures (h3ACs) at day 14 established in complete medium (left) or complete medium without CHIR99021 (right). Scale bar, 2000 μm. Images are representative of individual donors (n = 3). P0; passage 0.

(G) Representative brightfield and hematoxylin and eosin (H&E) images of primary HTII-280- derived 3D bronchial (airway) cultures (h3BCs) following 14 days of culture in human airway medium (Sachs et al., 2019). Scale bar, 2000 μm. P0; passage 0.

(H) IF images of h3BCs following 21 days of primary culture in (F). Cultures express airway lineage markers SCGB1A1 (for secretory, green; left), KRT5 (basal, white; left and right), and TP63 (basal, green; right), but not alveolar markers; pro-SFTPC (for hAT2, red, left), HTII-280 (for hAT2, red, right), and DAPI (blue). Scale bar, 50 μm. P0; passage 0.

(I) Serial passage of h3BCs via single cell dissociation at various time points depending on growth from 2 different donors (left). Each point represents a single passage for two separate donor samples (n= at least 3 technical replicates per donor). Quantification of colony forming efficiency for h3BCs at day 14 of culture up to 5 total passages (right). Data are presented as mean ± SEM for n = 2 biological samples. Each point represents the average of 3 technical replicates calculated for each biological sample.

(J) A representative karyotype image of h3ACs with 6 months in vitro culture (P5). No chromosomal aberrations were detected (n = 6, the number of examined hAT2 cells which are arrested in G2/M phase).

(K) Quantification of colony surface area during long-term serial culture of h3ACs. Areas are presented as the mean ± SEM of 300 colonies per passage across 3 technical replicates for 3 donor samples, with colonies consisting of a surface area of more than 1600 μm^2 being included in the analysis. Due to the low number of colonies at P8, only 100 colonies from 2 technical replicates were analyzed. (For each passage, n = 300 for donor 1, n = 300 for donor 2, n = 300 for donor 3 [P0-P4], n = 100 for donor 3 [P8]). n = total number of colonies scored.

Figure S2, related to Figure 2.

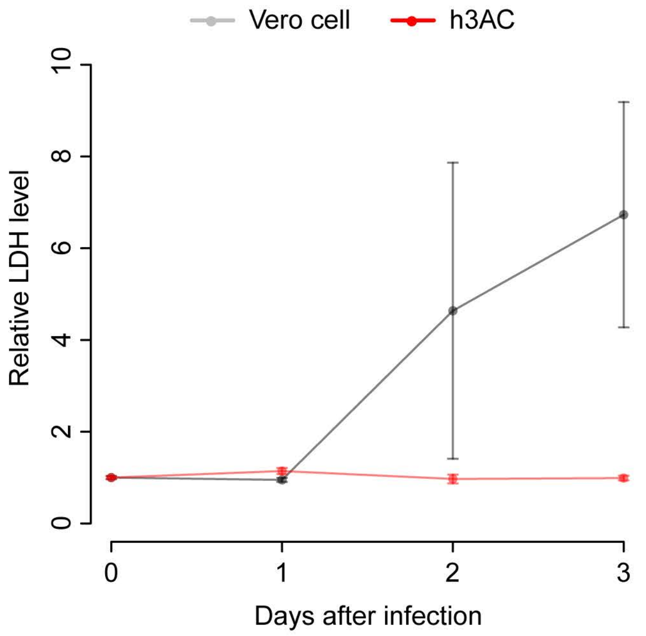

Figure S2. Lactate dehydrogenase cytotoxicity assay of human 3D alveolar cultures and Vero cells. Related to Figure 2.
Lactate dehydrogenase (LDH) is released into culture media when plasma membrane of cells is damaged. The cytotoxicity of SARS-CoV-2 infected Vero cells remarkably increases after 2 dpi, whereas that of SARS-CoV-2 infected h3ACs does not change significantly. Data is presented as mean ± SEM. n = 3 for biological replicates at each time point. h3ACs at passage 0 from 1 donor are used in the experiment.

Figure S3, related to Figure 3.

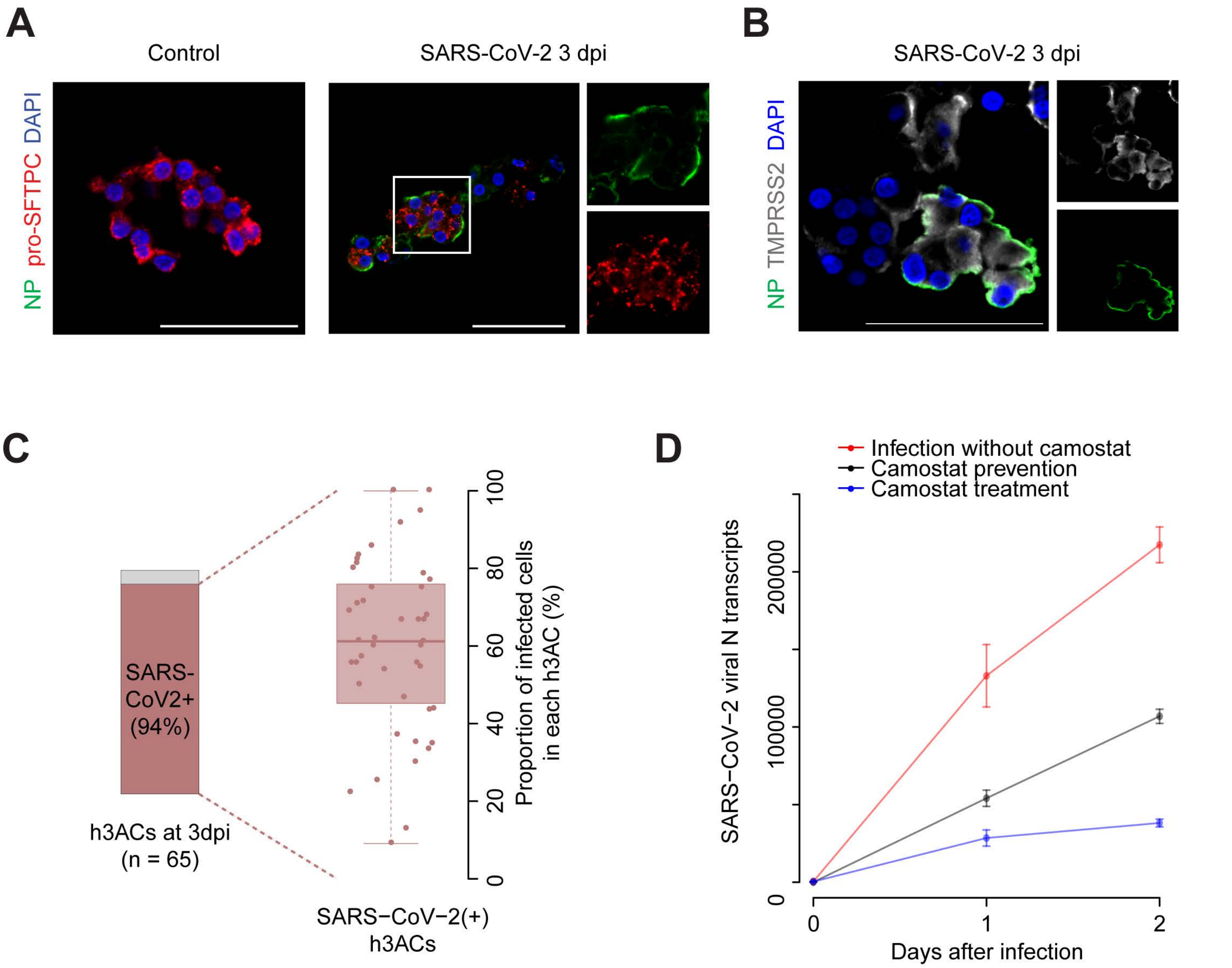

Figure S3. Confocal imaging analysis of infected h3ACs. Related to Figure 3.
(A) Representative IF images of infected h3ACs co-stained with SARS-CoV-2 nucleoprotein (NP, green) and pro-SFTPC (red) at 3 dpi (right). A representative IF image of uninfected control h3ACs is shown (left). Punctated patterns of pro-SFTPC are shown in magnified images (bottom right). Images are representatives of n ≥ 5 replicates of infected h3ACs (P2-3 from 3 donors). Scale bar, 50 μm.
(B) A representative IF image of infected h3ACs co-stained with SARS-CoV-2 nucleoprotein (NP, green) and TMPRSS2 (white) at 3 dpi. Images are representatives of 3 replicates of infected h3ACs (P2 from 2 donors). Scale bar, 50 μm.
(C) Proportion of h3ACs harboring hAT2 cells with viral components at 3 dpi (n = 65 h3ACs from P2-3 of 3 donors, left). The proportion of infected cells in the individual h3AC is depicted in the box plots (right).
(D) Camostat mesylate, a TMPRSS2 inhibitor, partially decreases the infectivity of SARS-CoV-2 in h3ACs. Both prevention (2 hours incubation with camostat mesylate before SARS-CoV-2 infection) and treatment (camostat mesylate treatment 2 hours after SARS-CoV-2 infection) are effective to reduce the burden of SARS-CoV-2 N gene transcripts. n = 3 for technical replicates at each time point. h3ACs with passage 2 from 1 donor are used.

Figure S4, related to Figure 4.

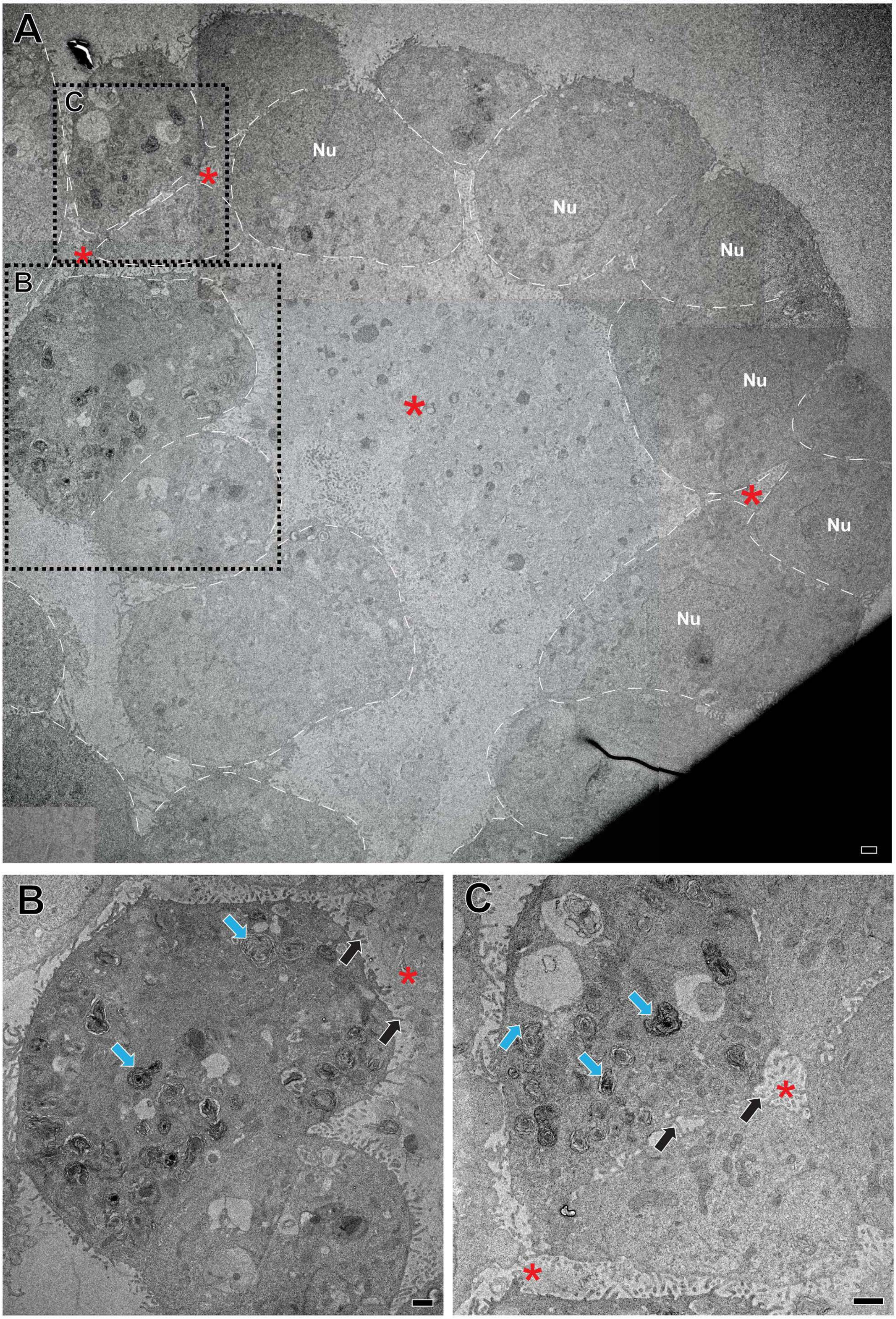

Figure S4. Transmission electron microscopy imaging of uninfected h3ACs. Related to Figure 4.
(A) A representative image of an uninfected h3AC (n = 10 h3ACs from 1 donor; P2). hAT2 cell membrane is delineated with white dashed line. Lumen is shown with a red asterisk. Nu: Nucleus. Data were manually stitched.
(B) A normal hAT2 cell showing multiple lamellar bodies (skyblue arrows). Microvilli in the luminal space of the h3AC are indicated by black arrow. No viral particle or pathologic large vacuoles are observed in the normal hAT2 cell.
(C) Another normal hAT2 cell. Multiple lamellar bodies are also shown in the hAT2 cell (skyblue arrow). Black arrow, microvilli in the lumen; red asterisk, lumen of the h3AC. All scale bars, 1 μm.

Figure S5, related to Figure 5.

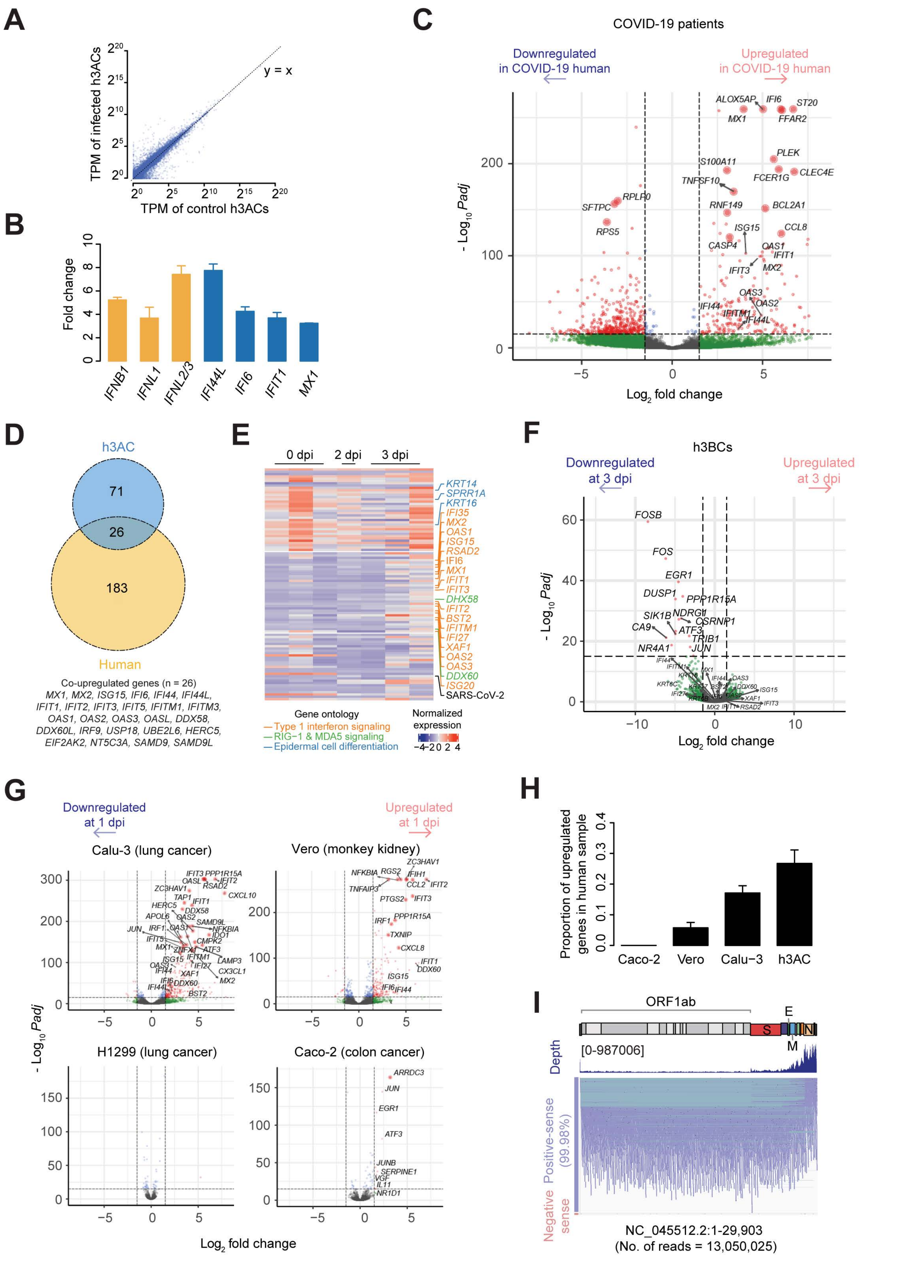

Figure S5. Transcriptome changes of the infected h3ACs. Related to Figure 5.
(A) Correlation of TPM level of each gene in h3ACs at 0 and 3 dpi. Each gene is depicted as a blue dot.
(B) qPCR validation for seven upregulated genes at 3 dpi identified by RNA sequencing. The seven genes include interferon genes (*IFNB1, IFNL1, and INFL2/3*) and ISGs (*IFI44L, IFI6, IFIT1, and MX1*). n = 3 technical replicates for each gene.
(C) Gene expression difference between healthy and SARS-CoV-2 infected human lung tissues (n = 8 from two donors for each group).
(D) A Venn diagram of upregulated DEGs in COVID-19 patients and SARS-CoV-2 infected h3ACs. Among the 97 DEGs, 26.7% of the DEGs, including 15 ISGs, are also increased in human COVID-19 patients.
(E) Heatmap of the most variable 100 genes derived from h3ACs (**Figure 5A, Table S1**) in SARS-CoV-2 infected h3BCs. Normalization of gene expression was calculated using all transcriptome data from 14 samples of h3ACs and h3BCs.
(F) Volcano plot for showing DEGs in h3BCs at 0 and 3 dpi. Expression levels of ISGs were not changed in h3BCs.
(G) Volcano plots for showing DEGs in 2D cell lines such as Calu-3, Vero, NCI-H1299, and Caco-2 cell lines. All DEGs are calculated between 0 and 1 dpi. Expression changes of ISGs are observed only in Calu-3.
(H) The proportion of upregulated DEGs found in COVID-19 patients in the upregulated DEGs of each model.
(I) Positive-sense viral RNAs are dominant (99.98%) over negative-sense viral RNAs in infected h3ACs at 1 dpi.

Figure S6, related to Figures 6 and 7.

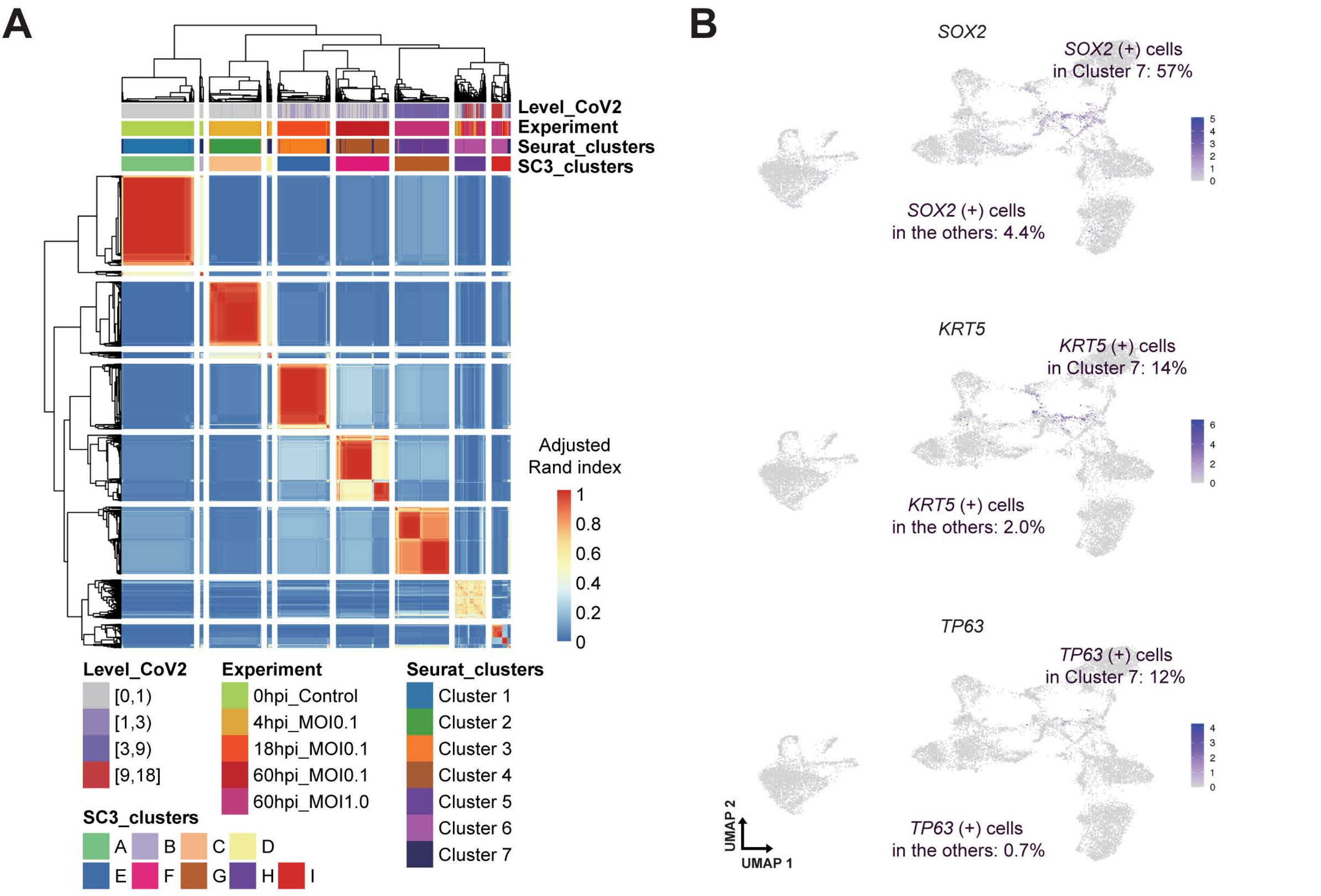

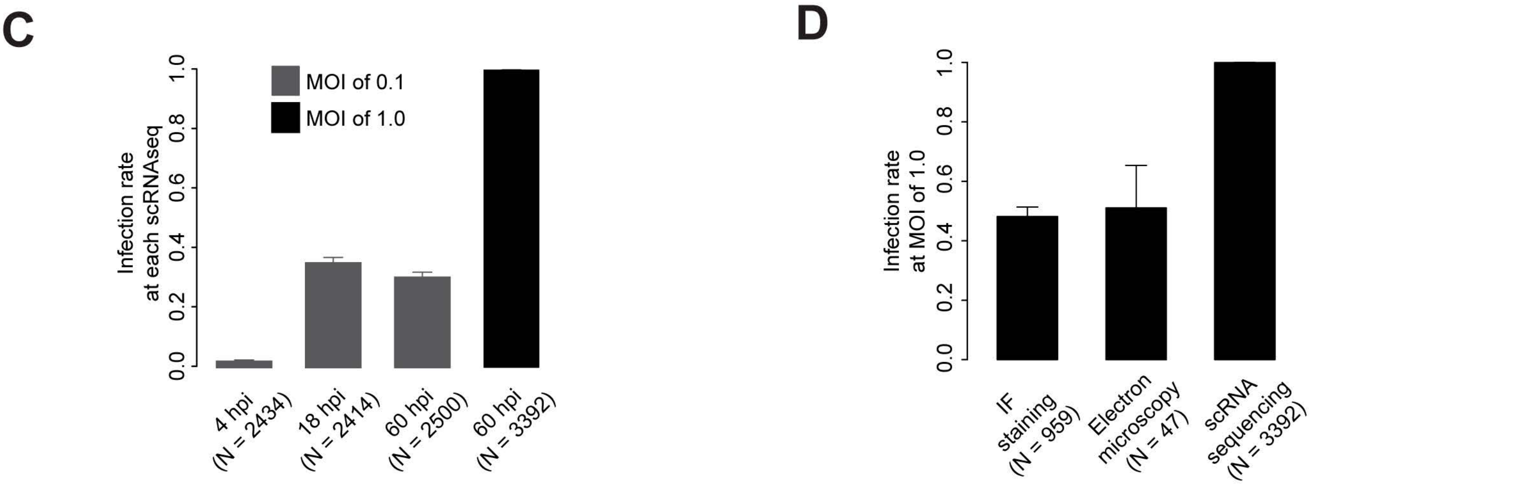

E

Significantly enriched GO biological process

	Gene Ontology Biological Process	Adj. *p-value*
1	Type I interferon signaling pathway (GO:0060337)	1.1×10^{-31}
2	Cellular response to type I interferon (GO:0071357)	2.3×10^{-31}
3	Cytokine-mediated signaling pathway (GO:0019221)	1.4×10^{-17}
4	Negative regulation of viral genome replication (GO:0045071)	5.0×10^{-14}
5	Negative regulation of viral life cycle (GO:1903901)	6.9×10^{-13}

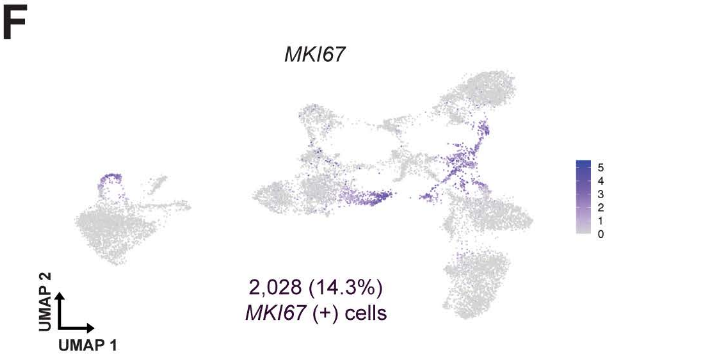

Figure S6. Single-cell transcriptome profiling of infected h3ACs. Related to Figures 6 and 7.
(A) Heatmap of unsupervised clustering of the single-cell RNA sequencing datasets using SC3 packages. The resulting SC3 clusters are in concordance with the Seurat clusters. Level_CoV2: log_2 values of SARS-CoV-2 UMI counts per 10k human UMI counts.
(B) In Cluster 7, the expression level of some airway markers, such as *SOX2, KRT5, TP63,* and *KRT5* are more expressed than other clusters, whereas the expression level of *SFTPB* and *NKX2-1*, hAT2 markers (**Figure 6E**), are reduced.
(C) The proportion of infected cells (≥1 viral UMI counts per 10k human UMI counts) in each experimental condition of single cell transcriptome. In experimental conditions with MOI of 0.1, more than 30% of cells are infected in 18 hpi and 60 hpi, while only 1.9% of cells are infected in 4 hpi. In contrast to MOI of 0.1, most cells (99.9%) at 60 hpi with MOI of 1.0 are infected. Error bars represent 95% of confidence intervals estimated by binomial distribution.
(D) The proportion of infected cells measured by NP+ cells (48.1%) in IF images at 3 dpi (N = 959, total counted number of cells), cells harboring viral particles (51.1%) in electron microscopic images at 2 dpi (N = 47), and cells with ≥1 viral UMI counts per 10k human UMI counts (99.9%) in infected h3ACS with MOI of 1.0 at 3 dpi (N = 3,392). Given the difference of detection methods (RNA or protein), the sensitivity of SARS-CoV-2 infected cells are different among techniques. Error bars represent 95% of confidence intervals estimated by binomial distribution.
(E) Top five significantly enriched gene ontology (GO) biological processes using top 150 variable genes (**Table S5**) selected from single cell transcriptome data. Pathways related to innate cellular responses to viral infection are significantly enriched.
(F) MKI67, a proliferation marker, is expressed not only in uninfected h3ACs, but also in infected h3ACs, which indicates that infected hAT2 cells still have the ability to proliferate.

Supplementary Tables

Table S6. Clinical characteristics of tissue donors. Related to Figures 1 and 2.

Patient ID	Hospital	Sex	Age	Diagnosis	Smoking status	Pack-year	Smoking_quit
1	Seoul National University Hospital	F	64	Lung adenocarcinoma	Never smoker	NA	NA
2	Seoul National University Hospital	F	41	Pulmonary sequestration	Never smoker	NA	NA
3	Seoul National University Hospital	M	57	Lung adenocarcinoma	Ex smoker	8	20 years ago
4	Seoul National University Hospital	M	61	Lung adenocarcinoma	Current smoker	25	0
5	Seoul National University Hospital	M	78	Lung adenocarcinoma	Never smoker	NA	NA
6	Seoul National University Hospital	F	54	Lung adenocarcinoma	Never smoker	NA	NA
7	Seoul National University Hospital	F	48	Lung adenocarcinoma	Never smoker	NA	NA
8	Seoul National University Hospital	F	68	Lung adenocarcinoma	Never smoker	NA	NA
9	Seoul National University Hospital	M	53	Lung adenocarcinoma	ex-smoker	10	14 years ago
10	Seoul National University Hospital	M	83	Lung squamous cell carcinoma	ex-smoker	10	45 years ago
11	Seoul National University Hospital	M	60	Lung adenocarcinoma	ex-smoker	10	25 years ago
12	Addenbrooks Hospital	F	52	Donation after brainstem death	Never smoker	NA	NA
13	Papworth Hospital Research Tissue Bank	F	74	Lung adenocarcinoma	Never smoker	NA	NA
14	Addenbrooks Hospital	F	61	Donation after brainstem death	Never smoker	NA	NA

NA: Not Applicable

Table S7. List of quantitative PCR primers. Related to STAR Methods.

REAGENTS	SOURCE	IDENTIFIER
2019-nCoV_N3 Forward: GGGAGCCTTGAATACACCAAAA	CDC	N/A
2019-nCoV_N3 Reverse: TGTAGCACGATTGCAGCATTG	CDC	N/A
IFI6 Forward: TGATGAGCTGGTCTGCGATCCT	This paper	N/A
IFI6 Reverse: GTAGCCCATCAGGGCACCAATA	This paper	N/A
IFIT1 Forward: GCCTTGCTGAAGTGTGGAGGAA	This paper	N/A
IFIT1 Reverse: ATCCAGGCGATAGGCAGAGATC	This paper	N/A
MX1 Forward: GGCTGTTTACCAGACTCCGACA	This paper	N/A
MX1 Reverse: CACAAAGCCTGGCAGCTCTCTA	This paper	N/A
IFI44L Forward: TGCACTGAGGCAGATGCTGCG	This paper	N/A
IFI44L Reverse: TCATTGCGGCACACCAGTACAG	This paper	N/A
IFNB1 Forward: CTTGGATTCCTACAAAGAAGCAGC	This paper	N/A
IFNB1 Reverse: TCCTCCTTCTGGAACTGCTGCA	This paper	N/A
IFNL1 Forward: GTTCAAATCTCTGTCACCAC	This paper	N/A
IFNL1 Reverse: TTCAGCTTGAGTGACTCTTC	This paper	N/A
IFNL2/3 Forward: GCCAAAGATGCCTTAGAAGAG	This paper	N/A
IFNL2/3 Reverse: CAGAACCTTCAGCGTCAGG	This paper	N/A
TaqMan probe : *SFTPC* (Hs00951326_g1)	Thermo Fischer Scientific	Cat#4331182
TaqMan probe : *TP63* (Hs01114115_m1)	Thermo Fischer Scientific	Cat#4331182
TaqMan probe : *SCGB1A1* (Hs00171092_m1)	Thermo Fischer Scientific	Cat#4331182
